# Personalized brain circuit scores identify clinically distinct biotypes in depression and anxiety

**DOI:** 10.1038/s41591-024-03057-9

**Published:** 2024-06-17

**Authors:** Leonardo Tozzi, Xue Zhang, Adam Pines, Alisa M. Olmsted, Emily S. Zhai, Esther T. Anene, Megan Chesnut, Bailey Holt-Gosselin, Sarah Chang, Patrick C. Stetz, Carolina A. Ramirez, Laura M. Hack, Mayuresh S. Korgaonkar, Max Wintermark, Ian H. Gotlib, Jun Ma, Leanne M. Williams

**Affiliations:** 1 Department of Psychiatry and Behavioral Sciences, Stanford University School of Medicine, Stanford, CA USA; 2 Sierra-Pacific Mental Illness Research, Education and Clinical Center, Veterans Affairs Palo Alto Health Care System, Palo Alto, CA USA; 3 Department of Counseling and Clinical Psychology, Teacher’s College, Columbia University, New York, NY USA; 4 Interdepartmental Neuroscience Graduate Program, Yale University School of Medicine, New Haven, CT USA; 5 Semel Institute for Neuroscience and Human Behavior, University of California, Los Angeles, CA USA; 6 Center for Intelligent Imaging, University of California San Francisco, San Francisco, CA USA; 7 Brain Dynamics Centre, Westmead Institute for Medical Research, University of Sydney, Westmead, New South Wales Australia; 8 Department of Radiology, Westmead Hospital, Western Sydney Local Health District, Westmead, New South Wales Australia; 9 Department of Neuroradiology, the University of Texas MD Anderson Center, Houston, TX USA; 10 Department of Psychology, Stanford University, Stanford, CA USA; 11 Department of Medicine, College of Medicine, University of Illinois Chicago, Chicago, IL USA

**Keywords:** Depression, Anxiety

## Abstract

There is an urgent need to derive quantitative measures based on coherent neurobiological dysfunctions or ‘biotypes’ to enable stratification of patients with depression and anxiety. We used task-free and task-evoked data from a standardized functional magnetic resonance imaging protocol conducted across multiple studies in patients with depression and anxiety when treatment free (*n* = 801) and after randomization to pharmacotherapy or behavioral therapy (*n* = 250). From these patients, we derived personalized and interpretable scores of brain circuit dysfunction grounded in a theoretical taxonomy. Participants were subdivided into six biotypes defined by distinct profiles of intrinsic task-free functional connectivity within the default mode, salience and frontoparietal attention circuits, and of activation and connectivity within frontal and subcortical regions elicited by emotional and cognitive tasks. The six biotypes showed consistency with our theoretical taxonomy and were distinguished by symptoms, behavioral performance on general and emotional cognitive computerized tests, and response to pharmacotherapy as well as behavioral therapy. Our results provide a new, theory-driven, clinically validated and interpretable quantitative method to parse the biological heterogeneity of depression and anxiety. Thus, they represent a promising approach to advance precision clinical care in psychiatry.

## Abstract (web summary)

Personalized brain circuit measures quantified using a new imaging technology in 801 patients with depression and anxiety identify six biotypes with unique symptoms, behaviors and responses to different types of treatment.

## Main

Depression and associated anxiety disorders are an important global public health burden^1^, the treatment of which has been hindered by etiological and phenotypic heterogeneity. The current psychiatric diagnostic system assigns a single label to syndromes that may involve the dysfunction of multiple and overlapping neurobiological processes which, in turn, would probably each require a different treatment. This is evident from the fact that more than a third of patients diagnosed with major depressive disorder, and approximately half of patients diagnosed with generalized anxiety disorder, do not respond to first-line treatment^2,3^. Unlike the ‘one-size-fits-all’ approach, a precision medicine approach to care requires standardized metrics that are personalized for individual patients and are interpretable to clinicians. However, the promise of this approach is currently limited by a lack of personalized and interpretable measures for quantifying neurobiological dysfunctions in patients with depression and associated anxiety disorders. We believe that such measures should help to elucidate the underlying neurobiological dysfunctions within a neuroscientific theoretical framework, rather than remain an algorithmic black box. Using these measures, patients could be stratified prospectively into subgroups that share similar neurobiological dysfunctions, or ‘biotypes’, each of which would possibly implicate a different set of treatment approaches or a different treatment trajectory.

Efforts to characterize biotypes of depressed and anxious patients with similar brain circuit dysfunctions have typically used task-free functional magnetic resonance imaging (fMRI)^4–7^. For example, one pioneering study has found biotypes characterized by aberrant connectivity in frontostriatal and limbic networks that respond differently to repetitive transcranial magnetic stimulation (TMS)^4^. Other researchers have found biotypes characterized by hyper- and hypoconnectivity of the default mode network^5^, biotypes that distinguish comorbid anxiety within the context of depression^6^ and biotypes that are associated with a poorer response to standard antidepressants^7^.

Nevertheless, we lack evidence about biotypes in depression and anxiety that are based on the participant-level quantification of measures derived from task-evoked imaging modalities. Patients with depression and anxiety exhibit dysfunction in the activity and connectivity of brain circuits in response to specific probes of general and emotional cognition. In other words, in depression and anxiety, the brain continually and flexibly engages different circuits under task-evoked and task-free conditions. Therefore, both sources of information may be useful in delineating biotypes and biotype-guided treatments. This is analogous to cardiac imaging being collected during both rest and task conditions in which the activity of the heart is elicited (for example, stress tests) to enable precise diagnoses and treatment plans, a necessity given the complexity of this organ and its functions^8^. Indeed, clinical trials have found that measures derived from task-based fMRI often predict response in depression treatment (for example, refs. ^9–12^) and have recently been the biomarker of choice for new pharmacotherapy development (for example, ref. ^13^).

Foundational studies using whole-brain, task-free connectivity biomarkers have often taken an unsupervised whole-brain approach that uses thousands of features for biotyping. However, we posit that clinical translation requires a theoretically informed approach that relies on a well-defined, tractable set of inputs. Such an approach also addresses the potential for obtaining overly optimistic results (overfitting) when thousands of inputs are used in a fully unsupervised manner—an issue that has been raised in the field^14^ (but see ref. ^15^, which addresses overfitting^11^).

Finally, previous studies have assessed the ability of biotypes to predict response to a single treatment (for example, TMS^4^ or antidepressants^7^), rather than comparing responses across different classes of treatments. To maximize the translational value of biotypes, the optimal treatment for each biotype should eventually be determined by comparing how different biotypes respond when receiving the same treatment.

In the present study, we demonstrate a new approach to generating biotypes of depression and anxiety based on task-evoked and task-free imaging data, quantified at the individual patient level and evaluated in the context of transdiagnostic symptoms, behaviors and outcomes with multiple types of treatments. Our approach relies on a standardized circuit quantification system that enables us to compute a manageable number of task-evoked and task-free measures of circuit function on an individual participant basis. These measures are firmly grounded in a theoretical synthesis of functional brain imaging studies that implicate dysfunction across large-scale circuits in the clinical features of depression and anxiety^16,17^. Thus, our theoretically driven approach provides unique insights that may have been missed by previous studies that either relied only on task-free data or mined large numbers of features using exploratory data analysis techniques. In our sample of 801 participants with depression and anxiety (95% of whom were unmedicated), the use of the same fMRI sequences, symptoms and behavioral measures enabled us to clinically validate theory-driven biotypes and demonstrate that they differ in symptom profiles and performance on general and emotional, cognitive, computerized behavioral tests. Furthermore, a substantial portion of the participants were enrolled into randomized clinical trials of antidepressants or behavioral therapy, which enabled us to demonstrate that our biotypes differ in their outcomes across multiple treatments.

## Results

### Personalized brain circuit scores define six biotypes

We began by implementing a new standardized image-processing procedure called ‘the Stanford Et Cere Image Processing System’ which quantified task-free and task-evoked brain circuit function at the level of the individual participants (Methods). We applied this procedure to a baseline dataset that consisted of brain scans acquired from both task-free and task conditions, utilizing identical scanning protocols, from 801 participants with depression and related anxiety disorders, as well as 137 healthy controls (Table 1 and Supplementary Table 1). At the time of baseline scanning, 95% of participants were not receiving any antidepressant treatments and none of the participants was diagnosed with a substance-dependent disorder. We used the same image-processing procedure in a treatment dataset consisting of 250 participants who were reassessed after completing treatment trials. During these trials, the participants were randomly assigned to receive one of three commonly prescribed antidepressant medications (escitalopram, sertraline or venlafaxine extended release (XR)^18^ (*n* = 164)) or an established behavioral intervention that integrated problem-solving with behavioral activation, compared with treatment as usual^19^ (*n* = 86) (Supplementary Tables 1 and 2).

**Table 1:** Demographics and diagnostic features of the sample used in the analyses

Feature	Clinical	Controls
Number	801	137
Sex		
Female, *n* (%)	461 (58)	67 (49)
Male, *n* (%)	329 (41)	70 (51)
Other, *n* (%)	11 (1)	0 (0)
Age (years), mean (s.d.)	34.24 (13.40)	32.10 (12.57)
Race		
American Indian/Alaska Native, *n* (%)	3	0 (0%)
Asian, *n* (%)	181 (23)	29 (21)
Black/African American, *n* (%)	16 (2)	1 (1)
Hawaiian/Pacific Islander, *n* (%)	1 (0)	0 (0)
More than one race, *n* (%)	31 (4)	4 (3)
Other, *n* (%)	103 (13)	6 (4)
White, *n* (%)	462 (58)	97 (71)
Treated at baseline, *n* (%)	40 (5)	0 (0)
Treatment arm		
Escitalopram, *n* (%)	46 (12)	0 (0)
Sertraline, *n* (%)	55 (11)	0 (0)
Venlafaxine, *n* (%)	50 (10)	0 (0)
I-CARE, *n* (%)	46 (9)	0 (0)
U-CARE, *n* (%)	40 (8)	0 (0)
Diagnoses		
Major depressive disorder, *n* (%)	375 (48)	0 (0)
Generalized anxiety disorder, *n* (%)	192 (28)	0 (0)
Panic disorder, *n* (%)	75 (10)	0 (0)
Social anxiety disorder, *n* (%)	179 (26)	0 (0)
Obsessive–compulsive disorder, *n* (%)	47 (7)	0 (0)
Post-traumatic stress disorder, *n* (%)	37 (5)	0 (0)
Comorbidity (2+ diagnoses)	221 (28)	0 (0)

For detailed information on the individual datasets used, see Supplementary Tables 1 and 2. *Diagnostic and Statistical Manual of Mental Disorders*, 4th edn, text revision (DSM-IV-TR) (RAD)^31^, DSM-5 (HCP-DES)^32^ or DSM-IV (iSPOT-D)^33^ criteria for major depressive disorder, anxiety disorder, post-traumatic stress disorder or obsessive–compulsive disorder were ascertained by a psychiatrist, general practitioner or research personnel using the structured interview, the Mini-International Neuropsychiatric Interview (MINI)^34^. In the ENGAGE sample, patients were considered eligible if they scored ≥10 on the Patient Health Questionnaire 9 (PHQ-9), a threshold with 88% specificity for major depressive disorder^35^, and had a qualifying BMI at study screening. Comorbidities were ascertained from electronic health records.

**Fig. 1:**
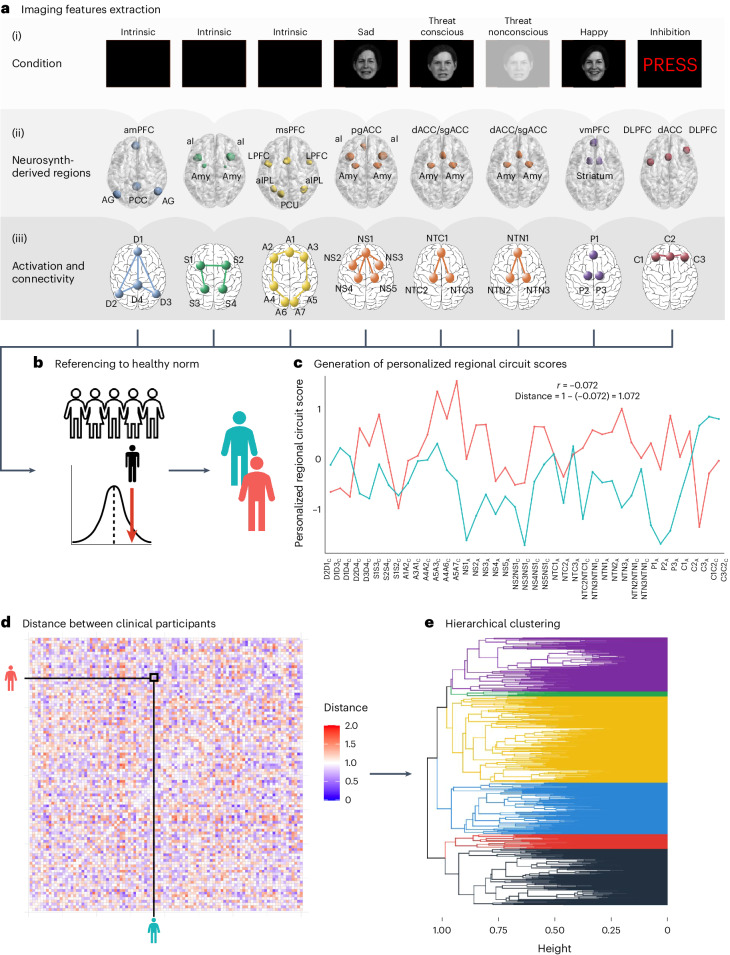
Overview of the participant-level image-processing and analysis pipeline. **a**, Measures of task-based activation and functional connectivity and task-free connectivity derived from regions belonging to six circuits for which we have established relevance to depression and anxiety. (i) Default mode (D), salience (S) and attention (A) circuits were derived from the task-free periods of the fMRI. The Negative and Positive (P) circuits were engaged by a facial expressions task. In particular, the Negative circuit was engaged in Threat Conscious (NTC), Threat Non-conscious (NTN) and Sad (NS) conditions. The cognitive control circuit (C) was engaged by a Go–NoGo task. (ii) We defined the regions of interest comprising each circuit from the meta-analytic platform Neurosynth and refined them based on quality control, a set of psychometric criteria and whether they were implicated in depression and anxiety. (iii) We extracted functional connectivity between circuit regions for task-free circuits, and activation and connectivity of regions for task-engaged circuits (regions shown as sphere, connectivity shown as lines). **b**, We then expressed these measures as s.d. values compared with healthy participants to obtain personalized regional circuit scores for each individual. See Supplementary Table 18 for the full list of scores. **c**, We computed the distance between each pair of individuals as 1 − the correlation of their regional circuit scores. **d**, We show the distance matrix between the first 100 participants as a heatmap for illustrative purposes. **e**, We then used the distances obtained as input for a hierarchical clustering analysis. The individuals depicted have given permission to be included in published facial emotion stimulus sets^36,37^. AG, angular gyrus; aI, anterior insula; aIPL, anterior inferior parietal lobule; amPFC, anterior medial prefrontal cortex; Amy, amygdala; dACC, dorsal anterior cingulate cortex; DLPFC, dorsolateral prefrontal cortex; LPFC, lateral prefrontal cortex; msPFC, medial superior prefrontal cortex; PCC, posterior cingulate cortex; PCU, precuneus; pgACC, pregenual anterior cingulate cortex; sgACC, subgenual anterior cingulate cortex; vmPFC, venteromedial prefrontal cortex.

Using our image-processing system, we obtained 41 measures of activation and connectivity of 6 brain circuits of interest for each participant^20^. We have previously shown that these circuit measures satisfy psychometric criteria for construct validation, internal consistency and generalizability^20^. A unique feature of our image-processing system is that quantified circuit measures are expressed in terms of s.d. units from the mean of a healthy reference sample, and thus are interpretable for each individual. We refer to the resulting measures as ‘regional circuit scores’ (Fig. 1 and see Supplementary Methods for details).

**Fig. 2:**
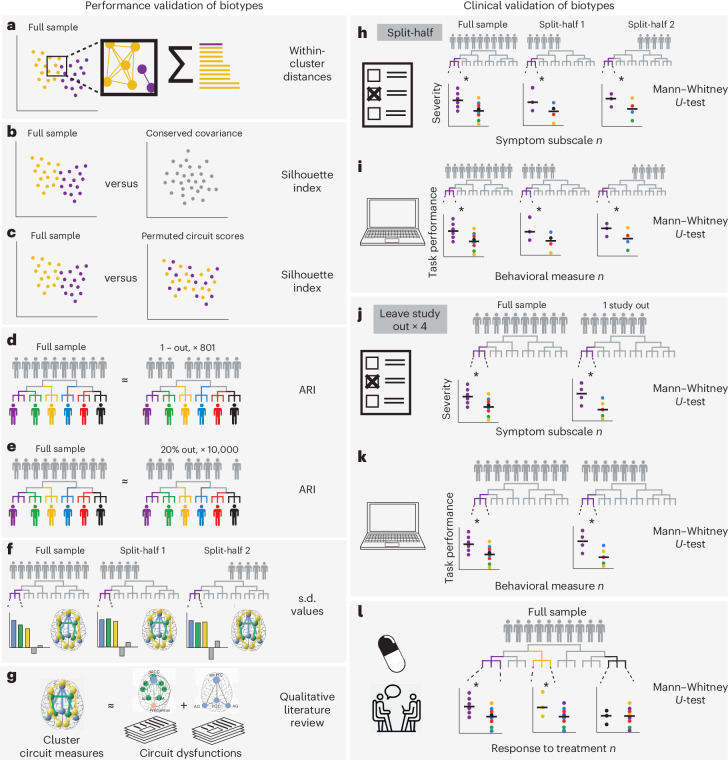
Overview of biotype validation. **a**, We selected candidate biotype solutions selected based on the sum of within-cluster distances. **b**, We evaluated the silhouette index of our solutions relative to a null multinormal distribution with conserved covariance between individuals^14^. **c**, We compared the silhouette index of our solutions relative to a solution using permuted participant labels, such that participant–brain correspondence was broken. **d**,**e**, We repeated our clustering approach leaving one participant out, 801× (**d**), as well as leaving out 20% of participants, 10,000× (**e**). In each iteration, we subsequently evaluated the overlap between participant biotype assignment in our original solution and each iterative solution by calculating the ARI. **f**, We evaluated the circuit measurements associated with each biotype across our original dataset and in two random halves of our original dataset separately. Circuit measurements that were consistently >0.5 s.d. from the mean across all these three samples were considered to be stable. **g**, We referenced the profile of circuit dysfunction to those found in the literature. **h**,**i**, To establish the clinical validity of our biotypes, we evaluated the cluster-specific differences in reported symptoms (**h**) and performances in a computerized cognitive battery (**i**). After establishing these differences in the full sample, we evaluated the stability of these symptom and behavioral profiles across two random half-splits of our data, deriving, each time, biotypes from the first half and assigning participants in the second half to a biotype derived from the first. We also followed the same procedure in a leave-study-out framework, leaving one of four of our studies out in each iteration. **j**,**k**, We subsequently evaluated the stability of biotype-specific symptom (**j**) and cognitive (**k**) differences relative to out-of-biotype participants in each iteration. We considered a difference to be stable when it was statistically significant in the whole sample and in each of the two random half-splits or in each of the two splits of a leave-study-out iteration. **l**, To evaluate the clinical utility of our cluster biotypes, we tested for differential symptom severity of each biotype to multiple depression treatments. Plots in this figure are only for illustrating the steps of our analysis.

To generate biotypes based on regional circuit scores of clinical participants, we used these scores as inputs for a hierarchical clustering algorithm (Fig. 1 and Methods). We generated solutions for 2–15 clusters and evaluated them as shown in Fig. 2.

### Biotype validation

We validated our biotypes using six convergent sources of evidence: the elbow method (Fig. 2a); two procedures proposed by Dinga et al.^14^ to evaluate the evidence for biotypes of depression and anxiety (simulation-based significance testing of the silhouette index (Fig. 2b) and stability using leave-one-out, and leave-20%-out crossvalidation (Fig. 2d,e)); an additional permutation-based significance testing of the silhouette index (Fig. 2c); split-half reliability of the cluster profiles (Fig. 2f); and the match of the solution to a theoretical framework of circuit dysfunction in depression and anxiety supported by previous brain imaging research^17^ (Fig. 2g).

The elbow method showed an elbow at five clusters and another, smaller elbow, at nine clusters, which suggested that the optimal solution lay between these two values (Supplementary Fig. 1). Simulation-based significance testing of the silhouette index showed that solutions with five or more clusters had a silhouette index that was significantly higher than that obtained by clustering data from a multivariate normal distribution (all *P* < 0.05; Supplementary Fig. 2) and significantly higher than that obtained by a permutation of the circuit scores across participants (*P* < 0.05; Supplementary Fig. 3). Assessment of cluster stability using crossvalidation showed that all solutions had good stability (adjusted Rand index (ARI) > 0.75 for leave-one-out and ARI > 0.28 for leave-20%-out) (Supplementary Fig. 4).

Across all validation analyses, six emerged as a viable number of clusters. The silhouette index tests comparing the data with data from a multivariate normal distribution and with a permutation of the circuit scores across participants were significant for this solution (mean silhouette = 0.065, *P* = 0.016 and *P* < 0.0001, respectively) and crossvalidation showed that it had good stability (leave-study-out ARI = 0.80 and leave-20%-out ARI = 0.35). Also, in the six-cluster solution, a cluster emerged that was characterized by reduced task-evoked activation during cognitive control, which we had specifically hypothesized^16,17^.

**Fig. 3:**
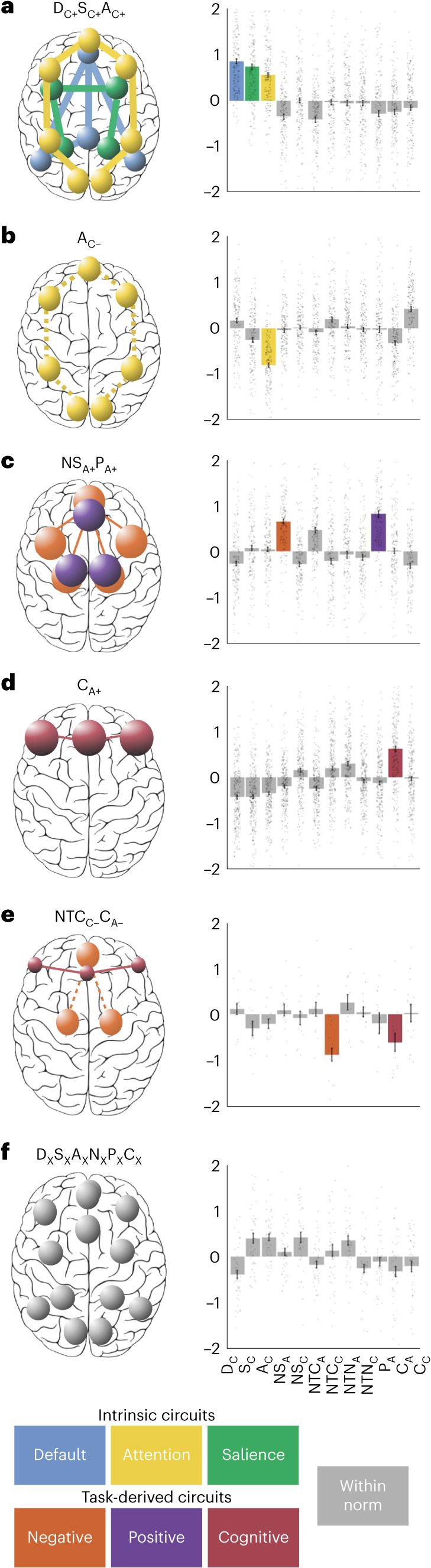
Clustering of regional brain circuit scores identifies six biotypes of depression and anxiety. **a**–**f**, Schematic circuit images illustrating the profile of circuit dysfunction defining each biotype (biotypes are labeled **a**–**f**). Circuits are distinguished by colors that correspond to the circuit measure inputs (Fig. 1c). Spheres represent the regions within each biotype-defining circuit and the size of the spheres represents the magnitude of activation deviation from the healthy reference (small spheres, activation ≤0.5 s.d. below the healthy reference; large spheres, activation ≥0.5 s.d. above the healthy reference). The thickness of lines between the spheres denotes a connectivity deviation (dashed lines, decreased connectivity ≤0.5 s.d. below the healthy reference; thick lines, increased connectivity ≥0.5 s.d. above the healthy reference). Column plots display the average activity across regions that define each circuit or the average connectivity between regions that define each circuit. A visualization of each regional circuit score by biotype is in Supplementary Fig. 5. In bar plots, we highlight circuits that showed a mean difference of at least 0.50 s.d. below or above the healthy reference. We named each biotype according to the features that differentiated it from the healthy reference. Each circuit is indicated with a letter, the distinguishing circuit feature is indicated as a subscript and the direction of dysfunction is indicated by + or −. The subscript x indicates that the sixth biotype is not differentiated by a prominent circuit dysfunction. Besides this nomenclature, we suggest a short description for each biotype, which connects them with our theoretically synthesized biotypes: D_C+_S_C+_A_C+_, default with salience and attention hyperconnectivity (*n* = 169 participants); A_C−_, attention hypoconnectivity (*n* = 161 participants); NS_A+_P_A+_, sad-elicited negative affect with positive affect hyperactivation (*n* = 154 participants); C_A+_, cognitive control hyperactivation (*n* = 258 participants); NTC_C−_C_A−_, cognitive control hypoactivation with conscious threat-elicited negative affect hypoconnectivity (*n* = 15 participants); and D_X_S_X_A_X_N_X_P_X_C_X_, intact activation and connectivity (*n* = 44 participants).

The six resulting biotypes were distinguished by specific profiles of both task-free and task-evoked activity and/or connectivity, relative both to each other and to our healthy reference sample. To assign a name to these distinctive circuit profiles, we determined which circuit features, activity or connectivity were distinguished by a difference of at least 0.50 s.d. in magnitude away from the healthy reference sample. The distinct activity and connectivity profiles of each biotype are illustrated using a circuit schematic and numerical plot in Fig. 3 with further details illustrated in bar plots in Supplementary Fig. 5. We named each biotype according to the circuits and circuit features that specifically differentiated them at this threshold relative to each other and to the healthy reference sample. We used the following nomenclature (each circuit is indicated with a letter): D, default mode; S, salience; A, attention; NS, negative affect circuit evoked by sad stimuli; NTC, negative affect circuit evoked by conscious threat stimuli; NTN, negative affect circuit evoked by nonconscious threat stimuli; P, positive affect circuit; C, cognitive circuit. The distinguishing circuit feature is indicated as a subscript: C, connectivity; A, activity, and the direction of dysfunction is indicated by + or −. These distinct profiles were also replicated when conducting the clustering procedure on a random half of the data and assigning participants in the second independent half of the data to each cluster (Supplementary Fig. 6).

Biotype D_C+_S_C+_A_C+_ (*n* = 169) was distinguished by relative intrinsic hyperconnectivity within the default mode circuit, as well as in the task-free salience and attention circuits (Fig. 3a). In contrast, biotype A_C−_ (*n* = 161) was distinguished by a relative reduction in intrinsic connectivity specific to the attention circuit (Fig. 3b). Biotype NS_A+_P_A+_ (*n* = 154) was characterized by heightened activity during conscious emotion processing, specifically within the negative affect circuit evoked by sad stimuli and within the positive affect circuit evoked by happy stimuli (Fig. 3c). Biotype C_A+_ (*n* = 258) was distinguished specifically by increased activity within the cognitive control circuit during the inhibition of NoGo stimuli (Fig. 3d). Biotype NTC_C-_C_A−_ (*n* = 15) was a smaller cluster differentiated by a relative loss of functional connectivity within the negative affect circuit during the conscious processing of threat faces, as well as by reduced (rather than heightened) activity within the cognitive control circuit during the inhibition of NoGo stimuli (Fig. 3e). Biotype D_X_S_X_A_X_N_X_P_X_C_X_ (*n* = 44) was not differentiated by a substantial circuit dysfunction relative to other biotypes or to the healthy norm; we indicated this by using the subscript x instead of + or − (Fig. 3f).

These distinct biotype circuit profiles were not explained by differences in scanners, because we removed scanner effects from our data using ComBat (Methods) and verified that the distribution of biotypes did not differ across scanners (*χ*^2^ = 12.773, two-sided *P* = 0.237).

### Biotypes differ on symptoms, behavior and treatment response

**Fig. 4:**
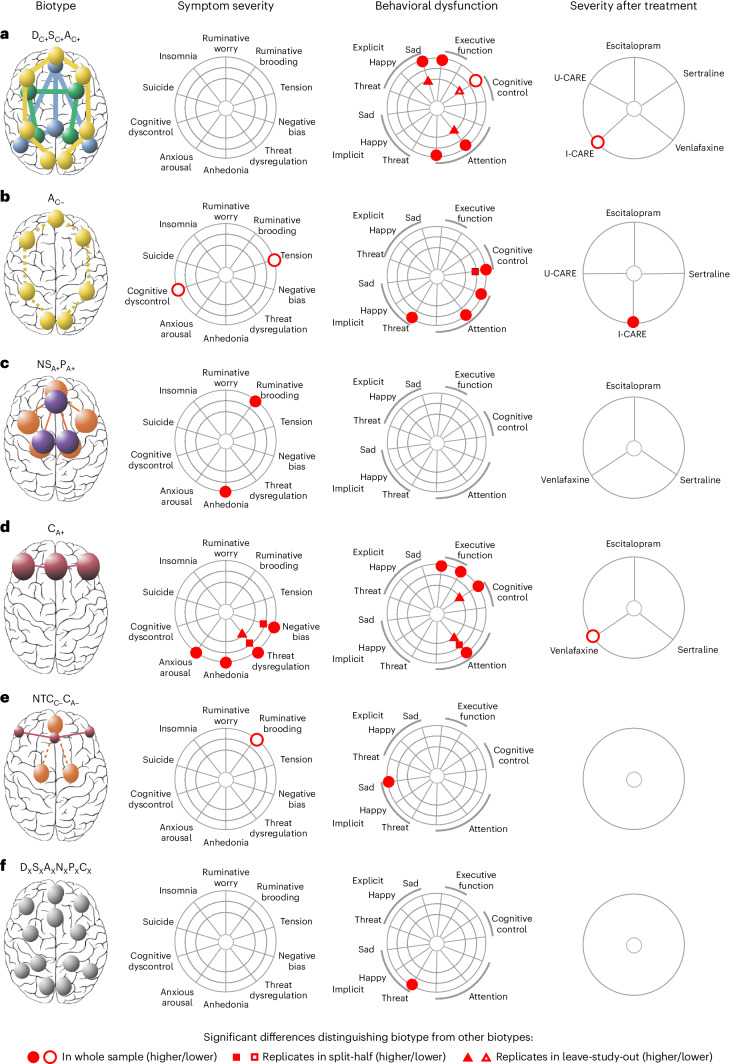
Summary results of clinical features distinguishing each biotype from the other biotypes. **a**–**f**, Circuit biotypes are visualized using circuit schematics on the left (biotypes are labeled **a**–**f**). We first compared these circuit biotypes on symptoms of depression and related anxiety (column ‘Symptom severity’). Next, we compared biotypes on behavioral performance on general and emotional cognitive tests relevant to social and occupational function (column ‘Behavioral dysfunction’). We compared biotypes on severity after treatment with one of three antidepressant pharmacotherapies (escitalopram, sertraline or venlafaxine XR), a behavioral problem-solving therapy (I-CARE) or usual care (U-CARE) (column ‘Severity after treatment’). To facilitate comparison across units of analysis, all measures were scaled between 0 and 1 so that 0 would represent minimum severity/dysfunction and 1 maximum severity/dysfunction. The column ‘Severity after treatment’ shows differences in symptom severity posttreatment (that is, lower values correspond to better treatment response). Comparisons on severity after treatment were conducted only for biotype/treatment combinations having *n* ≥ 5, so only those are shown. We used the biotype nomenclature used previously. The subscript x indicates that the sixth biotype is not differentiated by a prominent circuit dysfunction relative to other biotypes. Besides this nomenclature, we suggest a short plain-English description for each biotype (in quotes), which connects them with our theoretically synthesized biotypes (as shown in Fig. 3).

To further characterize the clinical phenotypes distinguished by each circuit biotype, we evaluated the biotype profiles on three different domains of clinically meaningful measures (Fig. 4): severity of symptoms, performance on general and emotional cognitive tests and differential treatment response. We highlight that the circuit biotypes derived from clustering were differentiated using only circuit inputs assessed independently from these domains of clinical information such that symptoms, performance and treatment response represented external validation measures.

We first asked whether the biotypes were distinguished by the severity of symptoms of depression and anxiety. To address this question, we used Mann–Whitney *U*-tests to compare the symptom severity of each biotype to the median symptom severity of all clinical participants not in the biotype (Supplementary Fig. 10 and Supplementary Tables 3 and 4). For insomnia and suicidality, these comparisons were conducted using *χ*^2^ tests instead (Supplementary Fig. 11 and Supplementary Table 5). We considered significant tests for which *P* < 0.05. We then replicated significant findings in split-half and leave-study-out analyses (Fig. 2h,j).

Second, we assessed whether biotypes are distinguished by performance on a computerized battery of general and emotional cognitive tests relevant to daily social and occupational function. We conducted these analyses as described above for symptoms (Supplementary Fig. 12 and Supplementary Tables 6 and 7). We then replicated significant findings in split-half and leave-study-out analyses (Fig. 2i,k).

Third, we assessed whether the biotypes predicted differential treatment response to one of the three pharmacotherapies or to behavioral therapy versus usual care. We conducted these analyses as described above for symptoms and behavior (Fig. 2l, Supplementary Fig. 13 and Supplementary Tables 8–10).

Biotype D_C+_S_C+_A_C+_, characterized by task-free circuit hyperconnectivity, had slowed behavioral responses in identifying sad faces (effect size (ES) = 0.289, *P* = 0.001, confidence interval (CI) = (−0.072, 0.289), replicated in leave-study-out), increased errors in an executive function task (ES = 0.175, *P* = 0.044, CI = (9−0.182, 0.166)), fewer commission errors in a cognitive control task (ES = −0.275, *P* = 0.002, CI = (−0.505, −0.217), replicated in leave-study-out) slowed responses to target stimuli in a sustained attention task (ES = 0.336, *P* = 0.0001, CI = (0.714, 1.099)) (see Fig. 4a and Supplementary Figs. 10–12 for detailed visualization and Supplementary Tables 3–7 for comparisons). The biotype D_C+_S_C+_A_C+_ responded better to I-CARE compared with other biotypes (ES = −0.612, *P* = 0.037, CI = (0.137, 0.306), responders = 42%, remitters = 25%) (Fig. 4a, Supplementary Fig. 13 and Supplementary Tables 8–10).

Biotype A_C-_, characterized by task-free attention circuit hypoconnectivity, had relatively less severe tension (ES = −0.196, *P* = 0.049, CI = (11.5, 15)), but was also differentiated by relatively lower cognitive dyscontrol (ES = −0.305, *P* = 0.006, CI = (15.5; 17.5)). In computerized tests, A_C−_ was distinguished by faster responses to target Go stimuli on the Go–NoGo task, (ES = −0.383, *P* = 6.20 × 10^−6^, CI = (0.180, 0.510), replicated in split-half), more commission and omission errors on the sustained attention task (ES = 0.300, *P* = 0.0004, CI = (−0.302, −0.019); ES = 0.198, *P* = 0.020, CI = (−0.308, −0.010)) and faster responses to priming by implicit threat stimuli (ES = −0.256, *P* = 0.002, CI = (−0.111, 0.112)) (see Fig. 4b and Supplementary Figs. 10–12 for detailed visualization and Supplementary Tables 3–7 for comparisons). The A_C−_ biotype had comparatively worse response to I-CARE (ES = 0.593, *P* = 0.002, CI = (0.219; 0.350), responders = 26%, remitters = 22%) (Fig. 4a, Supplementary Fig. 13 and Supplementary Tables 8–10).

Biotype NS_A+_P_A+_, distinguished by circuit hyperactivation during conscious emotion processing, was distinguished by more severe anhedonia (ES = 0.343, *P* = 0.014, CI = (2, 4.5)) and ruminative brooding (ES = 0.294, *P* = 0.036, CI = (55.5, 63)) (Fig. 4c; see Supplementary Figs. 10–12 for detailed visualization and Supplementary Tables 3–7 for comparisons).

Biotype C_A+_, distinguished by heightened activity within the cognitive control circuit, had more severe anhedonia than other biotypes (ES = 0.295, *P* = 0.015, CI = (2, 3.5)), more anxious arousal (ES = 0.218, *P* = 0.003, CI = (15.5, 17.5)), more negative bias (ES = 0.188, *P* = 0.003, CI = (15, 18.5), replicated in split-half) and more threat dysregulation (ES = 0.317, *P* = 5.07 × 10^−7^, CI = [7.5, 9], replicated in split-half and leave-study-out). Behaviorally, C_A+_ had more errors and completion time in the executive function task (ES = 0.164, *P* = 0.017, CI = (−0.268, −0.027) and ES = 0.152, *P* = 0.027, CI = (−0.164, 0.090)), more commission errors in the Go–NoGo task (ES = 0.158, *P* = 0.022, CI = (−0.201, 0.035), replicated in split-half) and more omission errors to target stimuli on the sustained attention task (ES = 0.275, *P* = 6.46 × 10^−5^, CI = (−0.045, 0.170), replicated in split-half and leave-study-out) (Fig. 4c; see Supplementary Figs. 10–12 for detailed visualization and Supplementary Tables 3–7 for comparisons). This biotype showed a better response to venlafaxine compared with the others (ES = −0.426, *P* = 0.034, CI = (0.132, 0.226), responders = 64%, remitters = 40%) (Fig. 4c, Supplementary Fig. 13 and Supplementary Tables 8–10**)**.

Biotype NTC_C-_C_A-_, differentiated by loss of functional connectivity within the negative affect circuit during the conscious processing of threat faces, as well as reduced activity within the cognitive control circuit, had less ruminative brooding compared with the other biotypes (ES = −0.902, *P* = 0.036, CI = (46, 5)), as well as faster reaction times to implicit sad faces (ES = −0.669, *P* = 0.024, CI = (−1.316, −0.315)) (Fig. 4d; see Supplementary Figs. 10–12 for detailed visualization and Supplementary Tables 3–7 for comparisons).

Biotype D_X_S_X_A_X_N_X_P_X_C_X_ was not differentiated by a prominent circuit dysfunction relative to other biotypes or the healthy norm; however, it was distinguished by slower reaction times to implicit threat priming (ES = 0.516, *P* = 0.001, CI = (0.254, 0.611)) (Fig. 4e; see Supplementary Figs. 10–12 for detailed visualization and Supplementary Tables 3–7 for comparisons).

Finally, we also considered the demographic factors of age and biological sex. The biotypes did not differ in sex distribution (*χ*^2^ = 12.643, *P* = 0.244) and only the A_C−_ biotype was, on average, slightly older than the other biotypes; importantly, however, participants in this biotype were still within the young to mid-adult age range (mean age: 39.69 years, s.d. = 15.739, *F* = 8.761, *P* = 4.21 × 10^−8^). Biotypes were also represented differently between datasets, which we expected given the clinical differences between the participants enrolled into each study (*χ*^2^ = 161.37, *P* = 2.2 × 10^−16^) (Supplementary Table 11).

As a context for the above evaluation of how biotypes were distinguished by symptoms, performance and treatment response, we evaluated the correlations between circuit scores and these external measures in the full sample across clusters combined (Supplementary Figs. 7–9). When thresholded with the false discovery rate correction for all pairwise correlations, we observed significant associations between circuit scores and 21% of the symptom measures, 10% of the performance measures and 31% of the treatment response measures.

### Biotypes are transdiagnostic

The distinct clinical and treatment profiles that distinguish the six biotypes indicate that these circuit-derived biotypes dissect the heterogeneity of the traditional diagnostic classification of depression. We next asked whether biotypes transcend diagnostic classifications across the diagnoses that are related to and comorbid with depression. Our sample was composed of participants who met traditional diagnostic criteria for major depressive disorder (*n* = 375), generalized anxiety disorder (*n* = 192), panic disorder (*n* = 75), social anxiety disorder (*n* = 179), obsessive–compulsive disorder (*n* = 47) and post-traumatic stress disorder (*n* = 37). Several participants also met criteria for more than one diagnosis (*n* = 221) (Table 1).

**Fig. 5:**
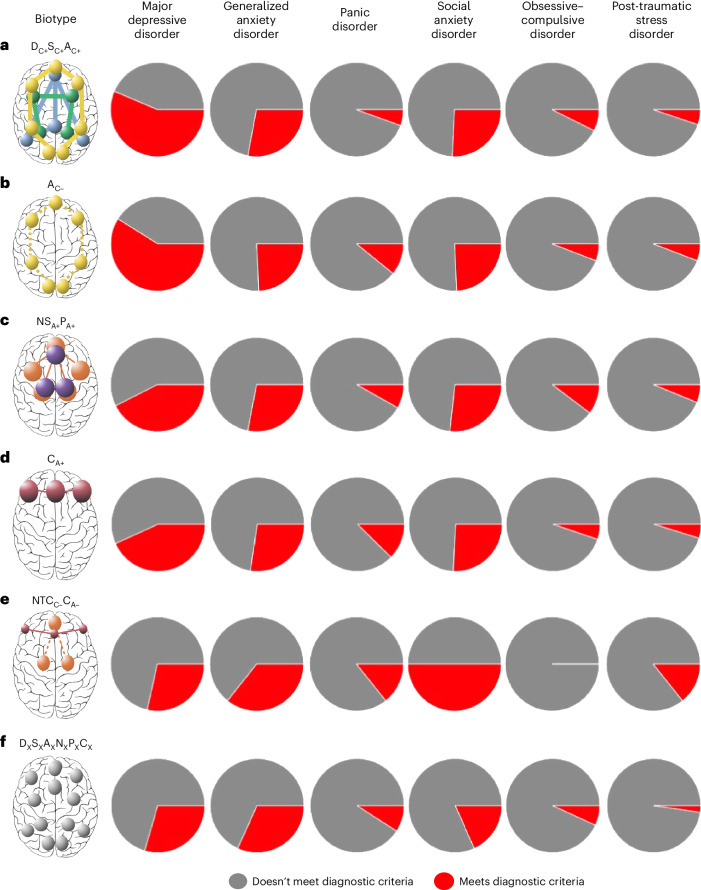
Frequency of diagnoses across biotypes. We show the proportion of participants in each biotype who meet diagnostic criteria for major depressive disorder, generalized anxiety disorder, panic disorder, social anxiety disorder, obsessive–compulsive disorder and post-traumatic stress disorder (biotypes are labeled **a**–**f**). *χ*^2^ tests revealed that the frequency of major depressive disorder was significantly different across biotypes (two-sided *χ*^2^ = 24.235, *P* = 0.0002). We used the same biotype nomenclature as previously. The subscript x indicates that the sixth biotype is not differentiated by a prominent circuit dysfunction relative to other biotypes. Besides this nomenclature, we suggest a short plain-English description for each biotype (in quotes), which connects them with our theoretically synthesized biotypes, again as expressed in the legend to Fig. 3.

The only diagnosis with a different frequency across biotypes was current major depressive disorder (*χ*^2^ = 24.235, two-sided *P* = 0.0002). In particular, the A_C−_ biotype had the highest proportion of participants with current major depressive disorder and the D_X_S_X_A_X_N_X_P_X_C_X_ cluster had the lowest proportion (Fig. 5 and Supplementary Table 12).

### Brain circuit scores outperform other features for biotyping

To compare prior approaches for biotyping with ours, we repeated our analysis using three competing alternative feature sets, each used in a recent paper reporting the identification of biotypes of depression using resting state fMRI. We then evaluated the results with the same criteria that we used for our own features (Fig. 2). Our findings show that our feature set is the only one that outperforms the null hypothesis of no clusters based on simulating data from a multinormal distribution with the same covariance as the original data (*P* = 0.016). In direct statistical comparisons of clustering performance between feature sets used as inputs, our combination of task and task-free regional circuit scores outperformed whole-brain connectomes (silhouette difference = −0.026, *P*_resample_ = 0.049, *P*_permute_ < 0.0001) and default mode network resting state connectivity (silhouette difference = −0.012, *P*_resample_ = 0.256, *P*_permute_ < 0.0001), but not connectivity of a network centered on the angular gyrus (silhouette difference = 0.155, *P*_resample_ = 1, *P*_permute_ = 1). The other feature sets also yielded associations among various metrics of biotypes, symptoms, behavioral performance and treatment response (Supplementary Tables 13 and 14).

To assess the impact of including task fMRI measures in addition to task-free brain circuit scores only, we also evaluated, in the same way, the results obtained using only our task-free brain circuit scores as input. To do so we showed that limiting the analysis to task-free brain circuit scores generated results that did not outperform the null hypothesis of no clusters based on simulating data from a multinormal distribution with the same covariance as the original data. Task-based brain circuit scores were also necessary to obtain symptom differences that generalize across random split-halves and behavior differences that generalize across the leave-study-out splits, depending on the number of clusters chosen (Supplementary Table 15).

## Discussion

To enable more precise diagnosis and selection of the best treatment for each individual, we need to dissect the heterogeneity of depression and anxiety. The dominant ‘one-size-fits-all’ diagnostic approach in psychiatry leads to cycling through treatment options by trial and error, which is lengthy, expensive and frustrating, with 30–40% of patients not achieving remission after trying one treatment^21^.

In the present study, we focus on the conceptualization of depression and anxiety as disorders of brain circuit function^22^. Using clustering and a new imaging system for the standardized quantification of circuit dysfunction at the level of the individual, we characterized six biotypes of depression and anxiety defined by specific profiles of dysfunction within both task-free and task-evoked brain circuits. These biotypes were validated using several procedures including simulations, crossvalidation and replication in held-out data. We found that the biotypes were distinguished by symptoms and behavioral performance on general and emotional cognitive tests that were not used as inputs in the clustering procedure. Importantly, some of these associations were replicated in split-half and leave-study-out procedures. We also showed that the six biotypes cut across the diagnostic boundaries of depression, anxiety and related comorbid disorders. Importantly for clinical translation, these biotypes predict response to different pharmacological and behavioral interventions.

We believe that this is the first identification of brain-derived biotypes that uses standardized personalized quantification of both task-free and task-evoked brain circuit dysfunctions and assesses response of the biotypes across different types of treatment. Rather than pursuing a fully data-driven approach, we integrated an unsupervised clustering analysis with a theoretical framework suitable for interpretability (Supplementary Table 16). We did this to minimize the possibility of overfitting and to generate solutions suited to the prospective selection of patients by biotype for future precision psychiatry trials. In this hybrid approach, each biotype was typified by a specific circuit dysfunction relative to a healthy norm, which mapped on to a unique transdiagnostic clinical phenotype.

Although our identification of six biotypes is one of many possible solutions to disentangling heterogeneity, these biotypes indicate that there may be multiple neural pathways that result in the clinical manifestation of depression and anxiety. By combining imaging data with clinical symptoms and behavior, we delineated clinical patterns that are consistent with the putative function of the circuits underlying each biotype. Importantly, although some biotypes were characterized exclusively by alterations in task-free intrinsic connectivity, others were characterized by alterations in task-evoked changes in activity and connectivity.

In the task-free state, D_C+_S_C+_A_C+_ was distinguished by hyperconnectivity of the default mode circuit, coupled with hyperconnectivity of both salience and attention circuits, correlating clinically with slowed emotional and attentional responses, replicated in split-half analyses. Although previous studies have reported circuit alterations in each of these circuits in depression and anxiety, our findings indicate that the D_C+_S_C+_A_C+_ biotype exhibits a combination of these alterations. In line with our theoretical taxonomy, the A_C+_ biotype demonstrated hypoconnectivity rather than hyperconnectivity within the frontoparietal attention circuit. This pattern corresponded to a clinical profile of lapses in concentration and impulsivity, replicated in split-half analyses.

Under task conditions, the NS_A+_P_A+_ biotype displayed heightened activation within subcortical and cortical brain regions associated with processing both sad and positive emotions. Clinically, this biotype also exhibited prominent anhedonia. This profile corresponds with previous findings of heightened activity in the medial prefrontal cortex in response to happy faces, which has been linked to levels of anhedonia^23,24^ and is consistent with our theoretical taxonomy. Increased activation of the amygdala is a common observation in depression in response to negative emotion^25,26^. Notably, biotype NS_A+_P_A+_ exhibits concurrent hyperactivation of the ventral striatum, which may indicate a negative bias alongside anhedonia^17^.

Two additional biotypes displayed contrasting dysfunctions within the cognitive control circuit. Biotype NTC_C−_C_A−_ exhibited reduced activation during a cognitive control task and decreased connectivity in processing threat consciously. These characteristics suggest impaired cognitive control which is also crucial for regulating emotions. In contrast, C_A+_ showed increased activation of the cognitive control circuit. This was associated with threat-related symptoms, negative bias and poorer cognitive control, as well as working memory performance, confirmed by both split-half and leave-study-out analyses. The replication of biotype C_A+_ reinforces its inclusion as an exploratory biotype in our theoretical taxonomy. Although early evidence suggested that heightened cognitive control activity might be compensatory and not necessarily linked to behavioral deficits^27^, our findings indicate that it is associated with specific cognitive–behavioral impairments. These findings highlight the importance of including task fMRI measures in future precision psychiatry studies and the value of using multimodal approaches to achieve more precise diagnoses in depression^28^.

Our approach enabled us to compare the efficacy of different treatments for each biotype to advance neurobiologically informed precision psychiatry. Collecting identical imaging and clinical measures across patients and treatments enabled us to compare the response of each biotype for three antidepressants, a behavioral intervention and treatment as usual. By doing so, we found that the D_C+_S_C+_A_C+_ biotype, characterized by hyperconnectivity of the default mode and other task-free circuits, was associated with a better response to behavioral treatment compared with the other biotypes. On the other hand, the biotype characterized by reduced attention circuit connectivity (A_C−_), had a worse response to behavioral treatment. Finally, biotype C_A+_, characterized by hyperactivation of the cognitive control circuit, had a better response to venlafaxine.

We delineated and validated biotypes using a small number of theoretically motivated features. By integrating theoretically grounded, task-evoked and task-free measures, our analysis provides unique insights that are complementary to those of foundational large studies that have analyzed task-free data using whole-brain techniques^4,15^. Nevertheless, as this is the first demonstration, to our knowledge, of a participant-level approach to cluster-derived biotyping using a small number of task-evoked and task-free features, our results should be interpreted with caution. Future studies are needed to investigate these biotypes in new datasets and to prospectively assign participants to treatment based on their biotypes. Also, we acknowledge that obtaining task fMRI measures can be more burdensome than collecting task-free measures only. We compared our results with results obtained using task-free data only and found that including both task and task-free data provided the best validation results, especially in beyond-chance clustering of subjects in feature space. In direct statistical comparisons of clustering performance, our combination of task and task-free regional circuit scores outperformed whole-brain connectomes, default mode network task-free connectivity and task-free regional circuit scores alone, but not connectivity of a network centered on the angular gyrus; however, the last approach did not provide generalizable symptom differences between clusters. Alternative feature sets also yielded several reproducible associations among clusters, symptoms and behavioral performance, consistent with the previous literature. This demonstrates that our approach, although potentially advantageous, does not negate the potential of other feature selection processes for depression biotyping. Future biotyping studies with both task-based and task-free data should consider comparing the performance of each.

Some strengths of our sample are that it represents the entire spectrum of depression and anxiety severity, is almost completely unmedicated (95%) and is recruited from a variety of settings. The sample also features common comorbidities that are often exclusion criteria. However, by including such a diverse population, we potentially reduce our ability to detect additional biotypes that might be more specific to certain clinical settings. It is also possible that some biotypes reflect contributions from comorbidities, which warrants replication in larger transdiagnostic samples. Another possibility is that biotypes are at least partially driven by differences in demographics between datasets. It would not be surprising, for example, if certain age groups belonged more to biotypes characterized by specific brain and clinical dysfunctions, because psychiatric symptoms, treatment response and brain biology all vary with age. We used identical imaging measures to evaluate biotypes across multiple treatments. However, some treatment groups within a biotype were small and could be unduly influenced by comorbidities or treatment design factors; therefore, it is important that the generalizability of our findings be tested by future large treatment studies. We also acknowledge that our imaging measures use a specific set of fMRI tasks that are not widely available. Future replications of our approach will be facilitated by the fact that our tasks are relatively short and easy to implement, as demonstrated by their adoption for large clinical trials such as iSPOT-D, ENGAGE and a recent trial using TMS in treatment-resistant depression^29^. Future studies could also evaluate whether similar clusters can be derived from different tasks that tap into similar domains and compare the results with ours. Our large sample allowed us to evaluate the generalizability of symptom and behavioral differences in split-half and leave-study-out validations. However, the number of participants of clinical trials was too small to perform such analyses for treatment response (*n* < 10 for 90% of comparisons; Supplementary Table 8). Future studies should apply our approach to clinical trial data to verify these findings, which should be interpreted prudently until they can be validated in new samples. Finally, the symptom differences between biotypes that we detected were mostly small, with effect sizes ranging from 0.08 to 0.90. The small size of these differences might be a reason why most comparisons did not reach statistical significance when splitting the dataset in two random halves or by study and analyzing each split independently. Small effect sizes in the association between imaging and symptom variables are common^30^, highlighting the need for consistent measures across studies and for finer-grained clinical measures. In the present study, we show the utility of combining four studies using standardized measures. We recommend interpreting the clinical results that did not survive our validation analyses with caution, but the present study is nevertheless a foundation to further test these results.

In conclusion, we leveraged personalized regional dysfunction scores grounded in a theoretical taxonomy of brain dysfunction in mood and anxiety disorders to identify six biotypes in a large transdiagnostic sample of unmedicated individuals with depression and anxiety. These biotypes differed significantly in symptom profiles, performance on behavioral testing and responses to multiple treatments. Our results validate a new theory-driven method for depression biotyping as well as a promising approach to advancing precision clinical care in psychiatry.

## Methods

### Samples

Data were obtained from four studies: International Study to Predict Optimized Treatment in Depression (iSPOT-D^18^, https://clinicaltrials.gov/ct2/show/NCT00693849), Research on Anxiety and Depression study (RAD^38^), Human Connectome Project for Disordered Emotional States (HCP-DES^39^) and Engaging self-regulation targets to understand the mechanisms of behavior change and improve mood and weight outcome (ENGAGE^40^, https://clinicaltrials.gov/ct2/show/NCT02246413). Clinical participants from these studies (*n* = 801) represented the full spectrum of severity of depression and anxiety disorders (see Table 1 and Supplementary Table 1 for details). Healthy controls (iSPOT-D, *n* = 67; HCP-DES, *n* = 70) were used as a reference group for building regional circuit scores from the imaging data (see below). Of the 801 clinical participants, 250 completed randomized controlled trials of either antidepressant pharmacotherapy for major depressive disorder (*n* = 164)^18^ or behavioral intervention for clinically substantial depressive symptoms and obesity (*n* = 86)^40^ (see Supplementary Table 2 for more details).

All participants provided written informed consent. Procedures were approved by the Stanford University Institutional Review Board (IRB, protocol nos. 27937 and 41837) or the Western Sydney Area Health Service Human Research Ethics Committee.

### MRI acquisition and preprocessing

Details of MRI sequences, fMRI tasks, MRI data quantification and quality control are given in Supplementary Methods.

#### Acquisition

Participants underwent the Stanford Et Cere Image Processing System protocol, which probes six brain circuits: default mode circuit, salience circuit, attention circuit, negative affect circuit, positive affect circuit and cognitive control circuit^17,20^. The Facial Expressions of Emotion Tasks probed the positive and negative affect circuits and a Go–NoGo task probed the cognitive control circuit. We derived measures of task-free function of the default mode, attention and salience circuits from the task data^41,42^. Task-free measures were independent of those obtained from the task conditions (Supplementary Fig. 14).

#### Preprocessing

The MRI data were preprocessed using fMRIprep^43^. We discarded scans if they contained incidental findings, major artifacts or signal dropouts or had >25% of volumes containing significant frame-wise displacement. An experienced rater (L.T.) also visually checked each scan, leading to the exclusion of 32 participants. Scans removed owing to excessive motion were: Go–NoGo task = 38, Conscious Facial Expressions of Emotion Task = 92, Non-conscious Facial Expressions of Emotion Task = 76 and task free = 51 (see Supplementary Table 17 for the number of scans passing criteria).

### Derivation of regional circuit scores

A summary of how regional circuit scores were obtained is given in the following sections (Fig. 1; see Supplementary Methods for details). We previously demonstrated that this system produces valid and clinically useful individual circuit clinical scores^20^.

#### Extraction of imaging features of interest

The regions of interest within six circuits of interest were defined from the meta-analytic platform Neurosynth^44^ (see Supplementary Table 18 for search terms and coordinates) and refined by removing regions that did not pass quality control or psychometric criteria. Of the remaining regions, we only retained 29 regions implicated in our theoretical synthesis of dysfunctions in depression and anxiety^20,38^. From these regions, we derived 41 features of activation, task-based and task-free connectivity for subsequent analyses^20^ (see Supplementary Table 18 and Supplementary Tables S5A and S5B in ref. ^20^ for details on the regions of interest and features). Our focus on regions defined from theory, meta-analyses and anatomy can lead to reliable and reproducible imaging measures. For example, activations within anatomically defined regions of interest have acceptable-to-high within-participant reliability^45^, as does connectivity within established brain networks^46^.

All following analyses used RStudio 2022.07.2, R v.4.1.3. Code for these analyses and the regions of interest to derive our imaging features are at https://github.com/leotozzi88/cluster_study_2023.

#### Imputation of missing values

As a result of missing scans and quality control, some regional circuit scores could not be computed for some participants: 7.57% for the default, salience and attention scores, 9.38% for the negative affect sad scores, 9.38% for the negative affect threat conscious scores, 6.72% for the negative affect threat nonconscious scores, 4.05% for the cognitive control scores and 9.38% for the positive affect scores. We imputed these values separately for each scanner by using multiple imputation by chained equations with random forests (R package miceRanger), using one iteration of a predictive mean matching model with the imaging features as the input.

#### Correction for scanner effects

We removed the potential confounding effect of between-scanner variability using ComBat^47–49^, an established method that uses an empirical Bayesian framework to remove batch effects.

#### Referencing to a healthy norm

All imaging features of the clinical participants were expressed in s.d. units relative to the mean and s.d. of healthy controls. These values are henceforth referred to as ‘regional circuit scores’ and represent the amount of dysfunction of each component of each circuit. Subsequent analyses were conducted on the regional circuit scores of the clinical participants only.

### Symptom measures

We used self-reported questionnaires to operationalize: ruminative worry (Penn State Worry Questionnaire—Abbreviated total^50^); ruminative brooding (Ruminative Response Scale total^51^); anxious arousal (Mood and Anxiety Questionnaire general distress subscale^52^); negative bias (Depression Anxiety and Stress Scale (DASS) depression subscale); threat dysregulation (DASS anxiety subscale); anhedonia (Snaith–Hamilton Pleasure Scale total^53^); cognitive dyscontrol (Barratt Impulsiveness Scale attentional impulsiveness subscale^54^); tension (DASS stress subscale); insomnia (Quick Inventory of Depressive Symptomatology—Self-Report Revised (QIDS-SR) sum of items 1–3 (ref. ^55^)); and suicidality (QIDS-SR item 12). In iSPOT-D, we used the Hamilton Depression Rating Scale (HDRS) total score as a measure of depression severity^56^ and, in ENGAGE, we used the Symptom Checklist 20 Depression Scale (SCL-20)^57^. See Supplementary Table 19 for the participants in each sample available for each measure.

### Clinical diagnoses

DSM-IV-TR (RAD), DSM-5 (HCP-DES) or DSM-IV (iSPOT-D) criteria for major depressive disorder, anxiety disorder, post-traumatic stress disorder or obsessive–compulsive disorder were ascertained by a psychiatrist, general practitioner or researcher using the structured MINI^34^. In ENGAGE, patients were considered eligible if they scored ≥10 on the PHQ-9, a threshold with 88% specificity for major depressive disorder^35^, and had a qualifying body mass index (BMI). Comorbidities were ascertained from electronic health records.

### Behavioral performance measures

Cognitive performance was assessed using WebNeuro^37,58,59^. We focused on the tests for which our regional circuit scores have been shown to predict performance^20^: sustained attention (omission errors, commission errors and reaction times in a continuous performance test); executive function (errors and completion time of a maze test); cognitive control (commission errors and reaction times in a Go–NoGo test); explicit emotion identification (reaction time to identify happy, sad, fearful and angry faces); and implicit priming bias by emotion (difference in reaction time in a face identification task when primed implicitly by happy, sad, fearful and angry faces compared with neutral faces). For analyses, we used the test performance referenced to an age-matched norm generated by WebNeuro (*z*-scores). See Supplementary Table 19 for the number of participants in each sample available for each measure.

### Treatment

In iSPOT-D, participants were randomized to one of three treatments: escitalopram (selective serotonin reuptake inhibitor (SSRI)), sertraline (SSRI) or venlafaxine XR (selective serotonin–norepinephrine reuptake inhibitor (SNRI))^18^. In ENGAGE, participants were randomized to either a behavioral intervention combining problem-solving, behavioral activation and weight loss (Integrated Coaching for Better Mood and Weight, I-CARE) or usual care (U-CARE)^19,40^. No treatment was administered in HCP-DES and RAD, so these studies were not considered in the treatment analyses.

### Identification of depression biotypes

To identify biotypes within our clinical participants, we used hierarchical clustering of their 41 regional circuit scores. We selected the optimal number of clusters using six convergent sources of evidence: the elbow method; two procedures proposed by Dinga et al.^14^ to evaluate biotypes of depression (simulation-based significance testing of the silhouette index and stability using crossvalidation); permutation-based significance testing of the silhouette index; split-half reliability of the cluster profiles; and the match of the solution to a theoretical framework^17^ (Fig. 2).

#### Hierarchical clustering

For each pair of clinical participants, we first computed the correlation coefficient of their 41 imaging-derived regional circuit scores (Fig. 1). Then, we computed the dissimilarity between each pair of clinical participants as 1 − this correlation (see ref. ^60^ for a similar approach). We used the between-individual dissimilarity matrix as input to hierarchical clustering using the average as agglomeration method.

#### Elbow method

The first source of evidence that we used to choose the optimal number of clusters was the elbow method, based on a plot showing the within-cluster sum of distances between participants for solutions between 2 and 15 clusters (Fig. 2a).

#### Simulation-based significance testing of silhouette

We tested the probability of our observed average silhouette index occurring under the null hypothesis of no clusters (that is, of the data coming from a multinormal distribution)^14^. For clusters between 2 and 15, we conducted 10,000 simulation runs, in which we drew 801 participants from a multinormal distribution that had the same mean and covariance for each regional circuit score as our data. These simulated participants were then used as input in hierarchical clustering, as described above, and the average silhouette index across participants was calculated. Thus, we obtained null distributions for these average silhouette indices. Finally, we calculated the proportion of average silhouette indices generated under the null that were greater than the one we obtained from our data (*P* value). We considered statistically significant solutions with numbers of clusters for which *P* < 0.05 (Fig. 2b).

#### Permutation-based significance testing of silhouette

For each number of clusters between 2 and 15, we shuffled each brain circuit score across subjects 10,000×, then repeated the hierarchical clustering as described above and calculated the average silhouette index. Thus, we obtained null distributions for these average silhouette indices. Finally, we calculated the proportion of average silhouette indices generated under the null that were greater than the one we obtained from our data (*P* value). We considered statistically significant solutions with numbers of clusters for which *P* < 0.05 (Fig. 2c).

#### Assessment of cluster stability using crossvalidation

To evaluate whether the clustering was stable under small perturbations to the data^14^, we repeated the clustering procedure 801×, each time with one participant left out. For each run and for each solution between 2 and 15 clusters, we calculated the similarity of the new cluster assignments to the original ones using the ARI (Fig. 2d). We then repeated this procedure while holding out 20% of the sample instead of one participant (Fig. 2e).

#### Matching of clusters to a theoretical framework

We identified the primary circuit dysfunction of each cluster by averaging the values of regional circuit scores by circuit and modality (task-based activity, task-based connectivity, task-free connectivity) and identifying the measures that showed a >0.5 s.d. absolute mean difference compared with the healthy norm. We then compared the profile of circuit dysfunction of each cluster with those hypothesized in a theoretical framework of circuit dysfunction in depression and anxiety^16,17^.

#### Split-half replication of cluster profiles

First, we split our dataset into two random samples of equal size. Then, we ran our clustering procedure on the first half-split. Then, we assigned each participant in the second split to one of the clusters obtained in the first half-split. To do so, we computed the mean circuit scores across all participants belonging to each cluster in the first half-split. Then, we calculated Pearson’s correlation coefficient between each participant’s brain circuit scores and these cluster-averaged scores. Each out-of-sample participant was assigned to the cluster for which this correlation was highest. Finally, we identified the primary circuit dysfunctions of each cluster in each split as described above (>0.5 s.d. absolute mean difference compared with the healthy reference data) and examined whether they replicated the circuit profiles found in the whole sample visually and by computing Pearson’s correlation coefficient of the mean profile dysfunction profile of each cluster between splits (Fig. 2f).

### Clinical characterization of biotypes

We characterized our final clustering solution by using external clinical measures independent of cluster inputs: symptoms, clinical diagnoses, performance on behavioral tests and treatment response. Importantly, we also replicated our findings in split-half and leave-study-out analyses (Fig. 2g–l).

#### Comparison of symptoms between biotypes

For each symptom, we compared the median severity of participants in each biotype to the median severity of participants who were not in the biotype using Wilcoxon’s tests. As insomnia and suicidality were assessed using only three and one QIDS-SR items, respectively, we used a *χ*^2^ test to compare the fraction of participants in the biotype who endorsed any of the items (total value >0) compared with participants who were not in the biotype. For Wilcoxon’s tests, we calculated the effect size *r* as the *z* statistic divided by the square root of the sample size and we considered significant tests for which *P* < 0.05 (Fig. 2h,j).

#### Comparison of behavioral performance between biotypes

For each of our behavioral performance measures, we compared the median performance of participants in each biotype with the median performance of participants who were not in the biotype using Wilcoxon’s tests. We calculated the effect size *r* as the *z* statistic divided by the square root of the sample size and we considered significant tests for which *P* < 0.05 (Fig. 2i,k).

#### Comparison of treatment response between biotypes

To obtain a comparable measure of symptom severity across our clinical trial datasets, we first scaled the measures of total HDRS scores (collected in iSPOT-D) and SCL-20 scores (collected in ENGAGE) between 0 and 1 based on the minimum and maximum values of each scale. We defined response as a decrease of at least 50% of symptom severity from baseline to follow-up and remission as follow-up HDRS ≤ 7 or SCL-20 ≤ 0.5. Then, for each treatment modality and each biotype, the severity of symptoms after treatment of participants in the biotype was compared with the median symptom severity of clinical participants not in the biotype using Wilcoxon’s tests. For these tests, we excluded biotypes in which only five or fewer participants received a treatment. We calculated the effect size *r* as the *z* statistic divided by the square root of the sample size and considered significant tests for which *P* < 0.05. (Fig. 2l).

#### Split-half replication of clinical associations

We replicated the significant comparisons of behavior and symptoms between biotypes found in the complete sample by splitting the sample into two random halves, repeating the clustering procedure on the first half and then assigning participants in the second half to the clusters obtained in the first half, as described above. We then conducted Wilcoxon’s tests as described above in each split and considered a result replicable if it was significant both in the original sample and in each of the split-half samples (for the second split, we conducted a confirmatory one-sided test).

#### Leave-study-out replication of clinical associations

For each of the four studies included in our dataset, we replicated the significant comparisons of behavior and symptoms between biotypes by splitting the sample into two subsets: one containing the participants who were not from that study and one containing participants from that study. Then, we repeated the clustering procedure on the first split and assigned participants in the second subset to the clusters obtained in the first split, as described above. We then conducted Wilcoxon’s tests as described above and considered a result replicable if it was significant in each of the two splits when holding out at least one study (for the second split, we conducted a confirmatory one-sided test).

#### Comparison of diagnoses between biotypes

To evaluate whether the clusters reflected traditional diagnostic categories, we used *χ*^2^ tests to compare the proportion of clinical participants in each biotype who met criteria for major depressive disorder, generalized anxiety disorder, obsessive–compulsive disorder, post-traumatic stress disorder, panic disorder or social phobia.

#### Comparison of covariates of no interest between biotypes

To verify that biotypes were not driven by scanner effects, we used *χ*^2^ tests to evaluate whether the proportion of participants in each cluster was different across scanners. Similarly, we used *χ*^2^ tests to examine the effects of gender and dataset and a one-way analysis of variance (ANOVA) to test whether different biotypes had different age distributions.

#### Comparison of brain circuit scores to other biotyping inputs

We selected three alternative feature sets, each used in a recent paper identifying biotypes of depression using resting state fMRI (to our knowledge, no prior publication has used task fMRI): whole-brain functional connectivity from the Power atlas^4^; functional connectivity in the default mode network^5^; and a functional connectivity of the angular gyrus^7^. We evaluated these features using the same criteria that we used for our own: (1) solution outperforms null hypothesis of no clusters (simulated data); (2) solution outperforms null hypothesis of no clusters (permuted data); (3) ARI (leave-one-out mean); (4) ARI (leave-20%-out mean); (5) generalizable cluster profiles across random split-half; (6) generalizable symptom differences across random split-half; (7) generalizable behavior differences across random split-half; (8) generalizable symptom differences across leave-study-out; (9) generalizable behavior differences across leave-study-out; and (10) biotypes differ in treatment response. For each of the alternative sets of features, we evaluated the number of clusters reported in the original paper and six clusters (the number that we chose in our analysis). We also conducted two statistical tests comparing clustering performance using our features with other features. First, a resampling test: we sampled 80% of participants, used each set of features to cluster their data and computed the corresponding average silhouette index over 10,000 iterations. For each set of alternative features, we considered as *P*_resample_ the fraction of samplings in which the silhouette index was higher than the one obtained with our features. Then a permutation test: after clustering each of the imaging feature sets, we randomly permuted the cluster assignments 10,000× and computed a silhouette score for each. This provided us with null distributions of the silhouette index for each feature set. We then calculated the difference between the null distribution of the silhouette index obtained using our features and each of the null distributions obtained from alternative features. We considered as *P*_permute_ the proportion of permutations in which the difference between the two null distributions was greater than that between the silhouette indices of the real solutions. We considered our features to provide a better clustering when *P*_permute_ < 0.05 and *P*_resample_ < 0.05.

Finally, we compared our original results to results obtained using only our task-free brain circuit scores, choosing as the number of clusters six (the number we chose in our analysis using all features) and two (the number of clusters with task-free dysfunction identified in our analyses).

### Reporting summary

Further information on research design is available in the Nature Portfolio Reporting Summary linked to this article.

## Online content

Any methods, additional references, Nature Portfolio reporting summaries, source data, extended data, supplementary information, acknowledgements, peer review information; details of author contributions and competing interests; and statements of data and code availability are available at 10.1038/s41591-024-03057-9.

### Supplementary information

Supplementary Information Supplementary methods, figures and tables.

Reporting Summary

## Data Availability

The datasets used in this analysis were collected as part of the iSPOT-D, RAD, HCP-DES and ENGAGE studies. These datasets are available upon request from Stanford BrainNet at https://www.stanfordpmhw.com/datasets. The BRAINnet repository meets the requirements for being public but also aligns with the procedures of other official public and scientific repositories such as HCP, ABCD and NDA. This choice is in line with the FAIRness guidelines and it respects the original funding requirements, allowing for appropriate source contributions and citations. Our approach is specifically designed for scientific use, which includes limiting access to for-profit entities to comply with the original funding stipulations and participant consent. Therefore, total open access is not feasible. Our intention is to provide public access that is consistent with the consent agreements and the original funding intentions, similar to the data shared through NIH repositories. On Stanford BRAINnet, we established a data access request form that screens users, similar to other public repositories.

## Code availability

Code for the analyses and the regions of interest used to calculate the clinical circuit scores is available at https://github.com/leotozzi88/cluster_study_2023.

## References

[1] Friedrich, M. J. Depression is the leading cause of disability around the world. *JAMA* **317**, 1517 (2017). 28418490 10.1001/jama.2017.3826

[2] Ansara, E. D. Management of treatment-resistant generalized anxiety disorder. *Ment. Health Clin.* **10**, 326–334 (2020). 33224690 10.9740/mhc.2020.11.326 PMC7653736

[3] Ruberto, V. L., Jha, M. K. & Murrough, J. W. Pharmacological treatments for patients with treatment-resistant depression. *Pharmaceuticals* **13**, 116 (2020). 32512768 10.3390/ph13060116 PMC7345023

[4] Drysdale, A. T. et al. Resting-state connectivity biomarkers define neurophysiological subtypes of depression. *Nat. Med* **23**, 28–38 (2017). 27918562 10.1038/nm.4246 PMC5624035

[5] Liang, S. et al. Biotypes of major depressive disorder: neuroimaging evidence from resting-state default mode network patterns. *Neuroimage Clin.* **28**, 102514 (2020). 33396001 10.1016/j.nicl.2020.102514 PMC7724374

[6] Price, R. B., Gates, K., Kraynak, T. E., Thase, M. E. & Siegle, G. J. Data-driven subgroups in depression derived from directed functional connectivity paths at rest. *Neuropsychopharmacology* **42**, 2623–2632 (2017). 28497802 10.1038/npp.2017.97 PMC5686504

[7] Tokuda, T. et al. Identification of depression subtypes and relevant brain regions using a data-driven approach. *Sci. Rep.* **8**, 14082 (2018). 30237567 10.1038/s41598-018-32521-z PMC6148252

[8] Patel, A. R. et al. Stress cardiac magnetic resonance myocardial perfusionimaging: JACC review topic of the week. *J. Am. Coll. Cardiol.* **78**, 1655–1668 (2021). 34649703 10.1016/j.jacc.2021.08.022 PMC8530410

[9] Goldstein-Piekarski, A. N. et al. Human amygdala engagement moderated by early life stress exposure is a biobehavioral target for predicting recovery on antidepressants. *Proc. Natl Acad. Sci. USA* **113**, 11955–11960 (2016). 27791054 10.1073/pnas.1606671113 PMC5081583

[10] Nguyen, K. P. et al. Patterns of pretreatment reward task brain activation predict individual antidepressant response: key results from the EMBARC randomized clinical trial. *Biol. Psychiatry* **91**, 550–560 (2022). 34916068 10.1016/j.biopsych.2021.09.011 PMC8857018

[11] Pilmeyer, J. et al. Functional MRI in major depressive disorder: a review of findings, limitations, and future prospects. *J. Neuroimaging* **32**, 582–595 (2022). 35598083 10.1111/jon.13011 PMC9540243

[12] Tozzi, L., Goldstein-Piekarski, A. N., Korgaonkar, M. S. & Williams, L. M. Connectivity of the cognitive control network during response inhibition as a predictive and response biomarker in major depression: evidence from a randomized clinical trial. *Biol. Psychiatry* **87**, 462–472 (2020). 31601424 10.1016/j.biopsych.2019.08.005 PMC8628639

[13] Krystal, A. D. et al. A randomized proof-of-mechanism trial applying the ‘fast-fail’ approach to evaluating κ-opioid antagonism as a treatment for anhedonia. *Nat. Med.* **26**, 760–768 (2020). 32231295 10.1038/s41591-020-0806-7 PMC9949770

[14] Dinga, R. et al. Evaluating the evidence for biotypes of depression: methodological replication and extension of Drysdale et al. (2017). *Neuroimage Clin.* **22**, 101796 (2019). 30935858 10.1016/j.nicl.2019.101796 PMC6543446

[15] Grosenick, L. et al. Functional and optogenetic approaches to discovering stable subtype-specific circuit mechanisms in depression. *Biol. Psychiatry. Cogn. Neurosci. Neuroimaging* **4**, 554–566 (2019). 31176387 10.1016/j.bpsc.2019.04.013 PMC6788795

[16] Williams, L. M. Defining biotypes for depression and anxiety based on large-scale circuit dysfunction: a theoretical review of the evidence and future directions for clinical translation. *Depress. Anxiety* **34**, 9–24 (2017). 27653321 10.1002/da.22556 PMC5702265

[17] Williams, L. M. Precision psychiatry: a neural circuit taxonomy for depression and anxiety. *Lancet Psychiatry* **3**, 472–480 (2016). 27150382 10.1016/S2215-0366(15)00579-9 PMC4922884

[18] Williams, L. M. et al. International Study to Predict Optimized Treatment for Depression (iSPOT-D), a randomized clinical trial: rationale and protocol. *Trials* **12**, 4 (2011). 21208417 10.1186/1745-6215-12-4 PMC3036635

[19] Ma, J. et al. Effect of integrated behavioral weight loss treatment and problem-solving therapy on body mass index and depressive symptoms among patients with obesity and depression: the RAINBOW randomized clinical trial. *JAMA* **321**, 869–879 (2019). 30835308 10.1001/jama.2019.0557 PMC6439596

[20] Goldstein-Piekarski, A. N. et al. Mapping neural circuit biotypes to symptoms and behavioral dimensions of depression and anxiety. *Biol. Psychiatry* **91**, 561–571 (2022). 34482948 10.1016/j.biopsych.2021.06.024 PMC9511971

[21] Gaynes, B. N. et al. What did STAR*D teach us? Results from a large-scale, practical, clinical trial for patients with depression. *Pschiatr. Serv.* **60**, 1439–1445 (2009). 10.1176/ps.2009.60.11.1439 19880458

[22] Scangos, K. W., State, M. W., Miller, A. H., Baker, J. T. & Williams, L. M. New and emerging approaches to treat psychiatric disorders. *Nat. Med.* **29**, 317–333 (2023). 36797480 10.1038/s41591-022-02197-0 PMC11219030

[23] Dichter, G. S., Kozink, R. V., McClernon, F. J. & Smoski, M. J. Remitted major depression is characterized by reward network hyperactivation during reward anticipation and hypoactivation during reward outcomes. *J. Affect. Disord.* **136**, 1126–1134 (2012). 22036801 10.1016/j.jad.2011.09.048 PMC3272083

[24] Keedwell, P. A., Andrew, C., Williams, S. C. R., Brammer, M. J. & Phillips, M. L. The neural correlates of anhedonia in major depressive disorder. *Biol. Psychiatry* **58**, 843–853 (2005). 16043128 10.1016/j.biopsych.2005.05.019

[25] Groenewold, N. A., Opmeer, E. M., de Jonge, P., Aleman, A. & Costafreda, S. G. Emotional valence modulates brain functional abnormalities in depression: evidence from a meta-analysis of fMRI studies. *Neurosci. Biobehav. Rev.* **37**, 152–163 (2013). 23206667 10.1016/j.neubiorev.2012.11.015

[26] Stuhrmann, A., Suslow, T. & Dannlowski, U. Facial emotion processing in major depression: a systematic review of neuroimaging findings. *Biol. Mood Anxiety Disord.* **1**, 10 (2011). 22738433 10.1186/2045-5380-1-10 PMC3384264

[27] Matsuo, K. et al. Prefrontal hyperactivation during working memory task in untreated individuals with major depressive disorder. *Mol. Psychiatry* **12**, 158–166 (2007). 16983390 10.1038/sj.mp.4001894

[28] Cuthbert, B. N. & Kozak, M. J. Constructing constructs for psychopathology: the NIMH research domain criteria. *J. Abnorm. Psychol.* **122**, 928–937 (2013). 24016027 10.1037/a0034028

[29] Williams, L. M. et al. Identifying response and predictive biomarkers for transcranial magnetic stimulation outcomes: protocol and rationale for a mechanistic study of functional neuroimaging and behavioral biomarkers in veterans with pharmacoresistant depression. *BMC Psychiatry* **21**, 35 (2021). 33435926 10.1186/s12888-020-03030-z PMC7805238

[30] Feng, C., Thompson, W. K. & Paulus, M. P. Effect sizes of associations between neuroimaging measures and affective symptoms: a meta-analysis. *Depress. Anxiety* **39**, 19–25 (2022). 34516701 10.1002/da.23215

[31] American Psychiatric Association. *DSM-IV-TR: Diagnostic and Statistical Manual of Mental Disorders* 4th edn (2000).

[32] American Psychiatric Association. *DSM-5: Diagnostic and Statistical Manual of Mental Disorders* 5th edn (2013).

[33] American Psychiatric Association. *DSM-IV: Diagnostic and Statistical Manual of Mental Disorders* 4th edn (1994).

[34] Sheehan, D. V. et al. The Mini-International Neuropsychiatric Interview (M.I.N.I.): the development and validation of a structured diagnostic psychiatric interview for DSM-IV and ICD-10. *J. Clin. Psychiatry* **59**, 22–33 (1998). 9881538

[35] Kroenke, K., Spitzer, R. L. & Williams, J. B. W. The PHQ-9. *J. Gen. Intern. Med* **16**, 606–613 (2001). 11556941 10.1046/j.1525-1497.2001.016009606.x PMC1495268

[36] Gur, R. C. et al. A method for obtaining 3-dimensional facial expressions and its standardization for use in neurocognitive studies. *J. Neurosci. Methods* **115**, 137–143 (2002). 11992665 10.1016/S0165-0270(02)00006-7

[37] Mathersul, D. et al. Explicit identification and implicit recognition of facial emotions: II. Core domains and relationships with general cognition. *J. Clin. Exp. Neuropsychol.* **31**, 278–291 (2009). 18720178 10.1080/13803390802043619

[38] Williams, L. M. et al. Developing a clinical translational neuroscience taxonomy for anxiety and mood disorder: protocol for the baseline-follow up Research domain criteria Anxiety and Depression (‘RAD’) project. *BMC Psychiatry* **16**, 68 (2016). 26980207 10.1186/s12888-016-0771-3 PMC4793523

[39] Tozzi, L. et al. The human connectome project for disordered emotional states: protocol and rationale for a research domain criteria study of brain connectivity in young adult anxiety and depression. *NeuroImage* **214**, 116715 (2020). 32147367 10.1016/j.neuroimage.2020.116715 PMC8597395

[40] Williams, L. M. et al. The ENGAGE study: integrating neuroimaging, virtual reality and smartphone sensing to understand self-regulation for managing depression and obesity in a precision medicine model. *Behav. Res. Ther.* **101**, 58–70 (2018). 29074231 10.1016/j.brat.2017.09.012 PMC8109191

[41] Elliott, M. L. et al. General functional connectivity: shared features of resting-state and task fMRI drive reliable and heritable individual differences in functional brain networks. *NeuroImage* **189**, 516–532 (2019). 30708106 10.1016/j.neuroimage.2019.01.068 PMC6462481

[42] Korgaonkar, M. S., Ram, K., Williams, L. M., Gatt, J. M. & Grieve, S. M. Establishing the resting state default mode network derived from functional magnetic resonance imaging tasks as an endophenotype: a twins study. *Hum. Brain Mapp.* **35**, 3893–3902 (2014). 24453120 10.1002/hbm.22446 PMC6869646

[43] Esteban, O. et al. fMRIPrep: a robust preprocessing pipeline for functional MRI. *Nat. Methods* **16**, 111–116 (2019). 30532080 10.1038/s41592-018-0235-4 PMC6319393

[44] Yarkoni, T., Poldrack, R. A., Nichols, T. E., Van Essen, D. C. & Wager, T. D. Large-scale automated synthesis of human functional neuroimaging data. *Nat. Methods* **8**, 665–670 (2011). 21706013 10.1038/nmeth.1635 PMC3146590

[45] Holiga, Š. et al. Test-retest reliability of task-based and resting-state blood oxygen level dependence and cerebral blood flow measures. *PLoS ONE* **13**, e0206583 (2018). 30408072 10.1371/journal.pone.0206583 PMC6224062

[46] Tozzi, L., Fleming, S. L., Taylor, Z., Raterink, C. & Williams, L. M. Test–retest reliability of the human functional connectome over consecutive days: identifying highly reliable portions and assessing the impact of methodological choices. *Netw. Neurosci.*10.1162/netn_a_00148 (2020). 10.1162/netn_a_00148 PMC7888485 33615097

[47] Fortin, J.-P. et al. Harmonization of cortical thickness measurements across scanners and sites. *NeuroImage* **167**, 104–120 (2018). 29155184 10.1016/j.neuroimage.2017.11.024 PMC5845848

[48] Fortin, J.-P. et al. Harmonization of multi-site diffusion tensor imaging data. *NeuroImage* **161**, 149–170 (2017). 28826946 10.1016/j.neuroimage.2017.08.047 PMC5736019

[49] Johnson, W. E., Li, C. & Rabinovic, A. Adjusting batch effects in microarray expression data using empirical Bayes methods. *Biostatistics* **8**, 118–127 (2007). 16632515 10.1093/biostatistics/kxj037

[50] DeLapp, R. C., Chapman, L. K. & Williams, M. T. Psychometric properties of a brief version of the Penn State Worry Questionnaire in African Americans and European Americans. *Psychol. Assess.* **28**, 499–508 (2016). 26375429 10.1037/pas0000208

[51] Parola, N. et al. Psychometric properties of the Ruminative Response Scale-short form in a clinical sample of patients with major depressive disorder. *Patient Prefer Adherence* **11**, 929–937 (2017). 28553085 10.2147/PPA.S125730 PMC5440078

[52] Wardenaar, K. J. et al. Development and validation of a 30-item short adaptation of the Mood and Anxiety Symptoms Questionnaire (MASQ). *Psychiatry Res.* **179**, 101–106 (2010). 20472297 10.1016/j.psychres.2009.03.005

[53] Snaith, R. P. et al. A scale for the assessment of hedonic tone the Snaith–Hamilton pleasure scale. *Br. J. Psychiatry* **167**, 99–103 (1995). 7551619 10.1192/bjp.167.1.99

[54] Patton, J. H., Stanford, M. S. & Barratt, E. S. Factor structure of the Barratt impulsiveness scale. *J. Clin. Psychol.* **51**, 768–774 (1995). 8778124 10.1002/1097-4679(199511)51:6<768::AID-JCLP2270510607>3.0.CO;2-1

[55] Rush, A. J. et al. The 16-Item quick inventory of depressive symptomatology (QIDS), clinician rating (QIDS-C), and self-report (QIDS-SR): a psychometric evaluation in patients with chronic major depression. *Biol. Psychiatry* **54**, 573–583 (2003). 12946886 10.1016/S0006-3223(02)01866-8

[56] Hamilton, M. in *Assessment of Depression* (eds Sartorius, D. N. & Ban, D. T. A.) 143–152 (Springer, 1986).

[57] Derogatis, L. R., Lipman, R. S. & Covi, L. SCL-90: an outpatient psychiatric rating scale–preliminary report. *Psychopharmacol. Bull.* **9**, 13–28 (1973). 4682398

[58] Williams, L. M. et al. Explicit identification and implicit recognition of facial emotions: I. Age effects in males and females across 10 decades. *J. Clin. Exp. Neuropsychol.* **31**, 257–277 (2009). 18720177 10.1080/13803390802255635

[59] Williams, L. M. A platform for standardized, online delivered, clinically applicable neurocognitive assessment. Preprint at *bioRxiv*10.1101/2023.08.28.553107 (2023).

[60] Urchs, S. G. et al. Functional connectivity subtypes associate robustly with ASD diagnosis. *eLife* **11**, e56257 (2022). 36444973 10.7554/eLife.56257 PMC9708070

