# Supplementary material for: Personalized brain circuit scores identify clinically distinct biotypes in depression and anxiety
Source: Nat Med. 2024 Jun 17;30(7):2076–87. doi: 10.1038/s41591-024-03057-9 (PMC11271415; doi:10.1038/s41591-024-03057-9)
Supplement: Supplementary file 1 — Supplementary methods, figures and tables. [file 41591_2024_3057_MOESM1_ESM.pdf]

nature medicine

Supplementary information https://doi.org/10.1038/s41591-024-03057-9

Personalized brain circuit scores identify clinically distinct biotypes in depression and anxiety

In the format provided by the authors and unedited

TABLE OF CONTENTS

SUPPLEMENTARY METHODS 3
Experimental Conditions used to Probe Circuits of Interest 3
Imaging Acquisition 3
Image Pre-processing 4
Quality Control 4
Task-evoked activation analysis 5
Task-free analysis 5
SUPPLEMENTARY FIGURES 6
Supplementary Figure 1: Sum of distances between participants for different numbers of clusters. 6
Supplementary Figure 2: Simulation-based significance testing of the silhouette index. 7
Supplementary Figure 3: Permutation-based significance testing of the silhouette index. 8
Supplementary Figure 4: Assessment of cluster stability using cross-validation. 9
Supplementary Figure 5: Biotypes identified by hierarchical clustering 10
Supplementary Figure 6: Split-half reliability of the biotype profiles. 12
Supplementary Figure 7: Correlation between symptoms and regional circuit scores. 14
Supplementary Figure 8: Correlation between behavioral performance and regional circuit scores. 15
Supplementary Figure 9: Correlation between treatment response and regional circuit scores. 16
Supplementary Figure 10: Between biotype comparisons for each symptom domain. 17
Supplementary Figure 11: Between biotype comparisons for insomnia and suicidality. 18
Supplementary Figure 12: Between biotype comparisons of behavioral performance. 19
Supplementary Figure 13: Between biotype comparisons of treatment outcomes. 20
Supplementary Figure 14: Correlation between task-free and task regional circuit scores. 21
SUPPLEMENTARY TABLES 22
Supplementary Table 1: Demographics and diagnoses of the sample used in the cross-sectional analyses. 22
Supplementary Table 2: Demographics and diagnoses of the sample used in the treatment analyses. 23
Supplementary Table 3: Significant between biotype comparisons for each symptom domain. 24
Supplementary Table 4: Between biotype comparisons for each symptom domain. 25
Supplementary Table 5: Between biotype comparisons for insomnia and suicidality. 27
Supplementary Table 6: Significant between biotype comparisons of behavioral performance. 28
Supplementary Table 7: Between biotype comparisons of behavioral performance. 29

Supplementary Table 8: Biotype distributions for number of participants receiving each treatment. 31
Supplementary Table 9: Between biotype comparisons of treatment outcomes. 32
Supplementary Table 10: Biotype distribution of treatment response and remission rates. 33
Supplementary Table 11: Biotype distribution of number of participants by dataset. 34
Supplementary Table 12: Biotype overlap with diagnoses 35
Supplementary Table 13: Comparison of the performance of our brain circuit features to other features. 36
Supplementary Table 14. Comparison of our brain circuit features to other features using six clusters. 38
Supplementary Table 15. Comparison of our brain circuit features to resting state features only. 40
Supplementary Table 16: Cluster-derived biotypes comparison with theoretically synthesized biotypes. 42
Supplementary Table 17: Number of scans passing quality check and motion criteria before imputation. 44
Supplementary Table 18: Imaging features and brain regions. 45
Supplementary Table 19: Number of symptom and behavioral measures in the cross-sectional analyses. 49
iSPOT-D FMRIPREP PROCESSING DETAILS 51
RAD fMRIPREP PROCESSING DETAILS 55
ENGAGE fMRIPREP PROCESSING DETAILS 59
HCP-DES fMRIPREP PROCESSING DETAILS 63

SUPPLEMENTARY METHODS

Experimental Conditions used to Probe Circuits of Interest
The six circuits of interest in this study were measured from functional magnetic resonance imaging (fMRI) data using a novel standardized image processing procedure called 'the Stanford Et Cere Image Processing System'. This system allows the quantification of task-free and task-evoked brain circuit function at the level of the individual participants. Default Mode, Salience and Attention circuits were derived from the task-free periods of the fMRI sequencing protocols using a previously established procedure [1]. Negative and Positive Affect circuits were engaged by a facial expressions task, and the Cognitive Control circuit was engaged by a Go-NoGo task, the design of which was as follows:

Facial Expressions of Emotion Task
A standardized set of 3D-evoked facial expression stimuli were presented in pseudorandom order, with five repeated blocks of eight stimuli per block for sad, threat evoked by fear, threat evoked by anger, and happy, relative to neutral blocks; duration of stimulus was 500ms and the interstimulus interval was 750ms [2]. Participants were instructed to actively attend in order to answer post-scan questions about these faces, and we monitored alertness using an eye tracking system. We also presented the same stimuli nonconsciously in a backward-masking design to prevent awareness; face stimuli were presented for 10 ms followed immediately by a neutral face mask stimulus for 150 ms, and with a stimulus onset asynchrony of 1250 ms to match that of the conscious condition [3].

Go-NoGo Task
'Go' trials (the word "press" in GREEN) required participants to respond as quickly as possible, while the 'NoGo' trials ("press" in RED) required participants to withhold responses. 180 Go and 60 NoGo stimuli were presented in pseudorandom order; stimulus duration was 500 ms each with an interstimulus interval of 750 ms [4].

Imaging Acquisition
MRI data was collected using a 3.0 Tesla GE Signa HDx (Sydney), a 3.0 Tesla GE MR750 Discovery (Stanford) and a 3.0 Tesla GE UHP (Stanford) (GE Healthcare, Milwaukee, Wisconsin) using an 8-channel head coil (Sydney) and 32-channel head coil (Stanford). The two Stanford scanners used identical sequences. Head motion was restricted with foam pads and participant alertness was monitored using an eye-tracking system. Head motion was also recorded, which was later subject to quality control and potential data exclusion on the premise of excess motion.

Stanford Sequences (RAD, HCP-DES, ENGAGE)
In RAD and ENGAGE, a T1-weighted structural scan was acquired using a 3D spoiled gradient echo (SPGR) sequence normalization into standard space: TR=0.008; TE=0.003; voxel size=1x1x1mm; number of slices=176; FOV=256x256; flip angle=11°. In HCP-DES, the T1 parameters were TE = 3.548 ms; MPRAGE TR = 2.84s; FA = 8, acquisition time = 8 min and 33 sec; field of view = 256 × 256 mm; 3D matrix size = 320 × 320 × 230; slice orientation = sagittal; angulation to AC-PC line; receiver bandwidth = 31.25 kHz; fat suppression = no; motion correction = PROMO; voxel size = 0.8 mm isotropic. Blood oxygenation level-dependent contrast functional images were acquired using echo-planar T2*-weighted imaging. Each whole brain volume consisted of 45 interleaved 3mm thick axial/oblique slices (74 x 74 matrix; TR=2000ms; TE=27.5ms; voxel size=3x3x3mm; FOV=222mm; flip angle=77°). Each of the three tasks acquired 154 volumes over 5 minutes and 8 seconds.

Sydney Sequences (iSPOT-D)
The T1-weighted structural scan was acquired in the sagittal plane using a 3D spoiled gradient echo (SPGR) sequence (TR = 8.3 ms; TE = 3.2 ms; flip angle = 11 degrees; TI = 500 ms; NEX = 1; ASSSET = 1.5; matrix =

256 x 256). A total of 180 contiguous slices, each 1 mm thick, covered the whole brain with an in-plane resolution of 1 mm x 1 mm. The functional images for each task were acquired using echo planar imaging (TR = 2500 ms; TE = 27.5 ms; matrix = 64 x 64; FOV = 24 cm; flip angle = 90 degrees). Forty slices, each 3.5 mm thick, covered the whole brain in each volume. Each of the three tasks acquired 123 volumes over 5 minutes and 8 seconds.

Image Pre-processing

For functional images, the first three volumes were removed to account for magnetization transfer artifacts before pre-processing. Pre-processing was performed using fMRIPrep 20.2.1 (iSPOTD) and fMRIPrep 20.2.3 (HCP-DES, ENGAGE, RAD) [5]. For details, the standardized methodology outputs from fMRIPrep for each study can be found at the end of the Supplementary Material.

Quality Control

The quality control reports generated by fMRIPrep were visually inspected for abnormalities by an experienced rater (L.T.) and scans with incidental findings, major scanner artifacts, and signal dropouts were discarded. Scans with more than 25% of volumes that contained significant frame-wise displacement as defined by fMRIPrep were also discarded. This threshold was chosen to maximize applicability to real world, clinical settings and to be consistent with the original design of the iSPOT-D pragmatic biomarker trial.

Derivation of Regions of Interest

The derivation of regions of interest (ROIs) is described in detail in a previous publication [6].

In summary, an anatomical definition of subcortical nodes was combined with an automated meta-analysis approach to cortical nodes using neurosynth.org [7]. Neurosynth uniformity (previously called forward- inference) maps were used with a false detection rate (FDR) threshold of .01 for each circuit and defined our ROIs (see **Supplementary Table 18** for Neurosynth search terms). A set of peaks associated with each circuit's search term were then identified using AFNI's 3dExtrema function. Because some terms yielded maps with excessively large spatial extent, a restriction was imposed that each peak have a minimum z-score of 6 and each region extend no farther than 10mm from the peak. For subcortical regions, neurosynth maps were restricted by anatomically defined boundaries from the AAL atlas [8] plus an additional anatomical boundary defining the ventral striatum from the FSL atlas [9]. The Talairach atlas was used to identify the anatomical location of the peak of each region, and visual inspection of masks confirmed or adjusted these automatically derived labels.

In order to refine and maximize the quality of circuit definitions, we implemented the following steps in two healthy reference samples (see [6] for details). In the first sample, each individual's gray matter was identified by warping the output of FSL's FMRIB's Automated Segmentation Tool (FAST) to the MNI template. Each ROI was limited to gray matter only using this procedure. Using the second reference sample, we excluded ROIs with less than 50% average overlap between the original ROI and gray matter.

Next, in the first reference sample, ROIs were excluded if 95% of subjects had a temporal signal to noise ratio (tSNR) two standard deviations above the mean tSNR of a gray matter region with considerable signal drop out (peak coordinates 2, 46, -16, mean tSNR=47.03).

To further establish the internal validity of circuit definitions, the internal consistency of functional connectivity between pairs of regions was assessed, excluding region pairs for which connectivity (both task and task-free) showed stronger associations with out-of-circuit region pairs than with within-circuit region pairs in a healthy sample (see [6] for details).

For the current study, we selected the subset of regions most strongly implicated in circuit dysfunction in depression and anxiety in our theoretical synthesis [10]. These regions are the same that have been used for the derivation of circuit scores in [6].

Task-evoked activation analysis

The task-evoked analysis was conducted using SPM8 (https://www.fil.ion.ucl.ac.uk/spm/) and MATLAB version 2018b (MathWorks).

Task-evoked activation was quantified using a generalized linear model (GLM) in which task events were convolved with a canonical hemodynamic response function as implemented in SPM8. In this analysis, a 128s high pass filter was applied to the data, and six realignment parameters as well as white matter and cerebrospinal fluid signals derived by fMRIPrep were added to the design matrix as confounds. Residuals from these models were saved and used for the estimation of task-free connectivity (see below). Specific contrasts of interest were then computed for each circuit as follows: 1) negative affect circuit: sad > neutral conscious faces; 2) negative affect circuit: threat > neutral conscious faces; 3) negative affect circuit: threat > neutral non-conscious faces; 4) positive affect circuit: happy > neutral conscious faces; 5) cognitive control circuit: NoGo > Go trials. Measures of activation for each region of each circuit were obtained by extracting the average value of the contrast of interest.

To quantify task-based functional connectivity, we computed psychophysiological interactions (PPI) between pairs of regions belonging to the same circuit. For each region in each circuit (PPI seed), we calculated the first eigenvariate of that region's time series and fit a whole-brain first-level GLM as described above, which consisted of the psychological variable (task contrast of interest), physiological variable (region time course), and the interaction between psychological and physiological variables (PPI effect of interest). Then, we computed the average PPI effect of interest in specific regions belonging to the same circuit in accordance with our hypothesized model of circuit dysfunction (PPI targets) (**Figure 1**). To account for the fact that regions were used once as PPI targets and once as PPI seeds in this calculation, we averaged these results, yielding a single PPI value for each connection.

Task-free analysis

The task-free analysis was conducted using FSL version 5.0.10 [11] and MATLAB version 2018b (MathWorks).

Task-free data were derived following an established procedure [1]. First, the residuals of the task effects were saved from the GLM analysis described above. Then, these residuals were band-pass filtered between 0.08 and 0.009 Hz using FSL and concatenated across tasks. We then calculated from these data the correlation coefficient of the timeseries of each region pair belonging to the default mode, attention, and salience circuits. Finally, these values were converted to Fisher z and used as measures of task-free functional connectivity.

SUPPLEMENTARY FIGURES

Supplementary Figure 1: Sum of distances between participants for different numbers of clusters.

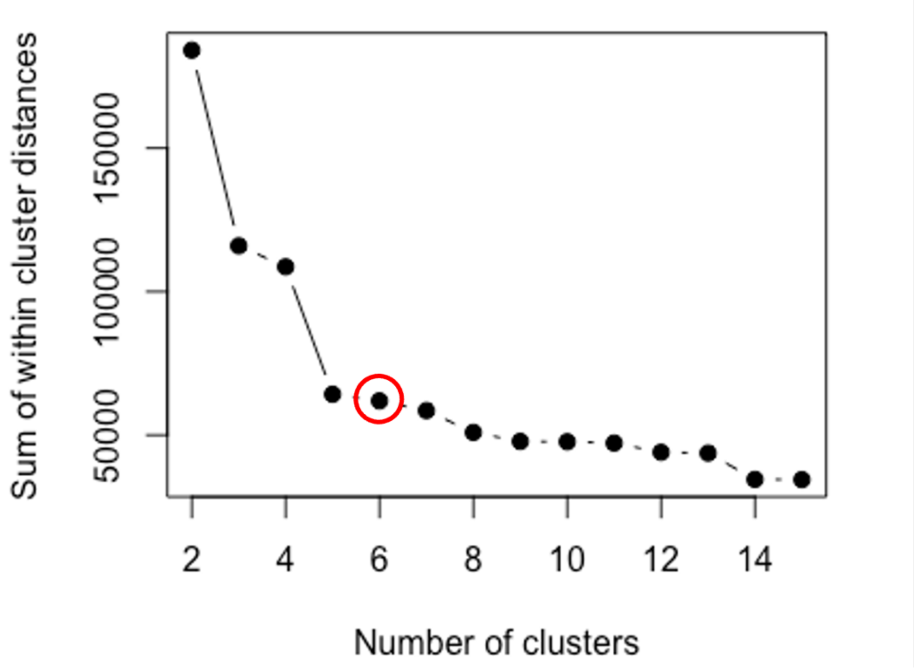

The plot showed an elbow at 5 clusters and another, smaller one at 9, which suggests that the optimal solution could lie between these two values. We selected 6 as the optimal number of clusters using four convergent sources of evidence: the elbow method, permutation-based significance testing of the silhouette index, stability using cross-validation; and the match of the solution to a theoretical framework (circled in red).

Supplementary Figure 2: Simulation-based significance testing of the silhouette index.

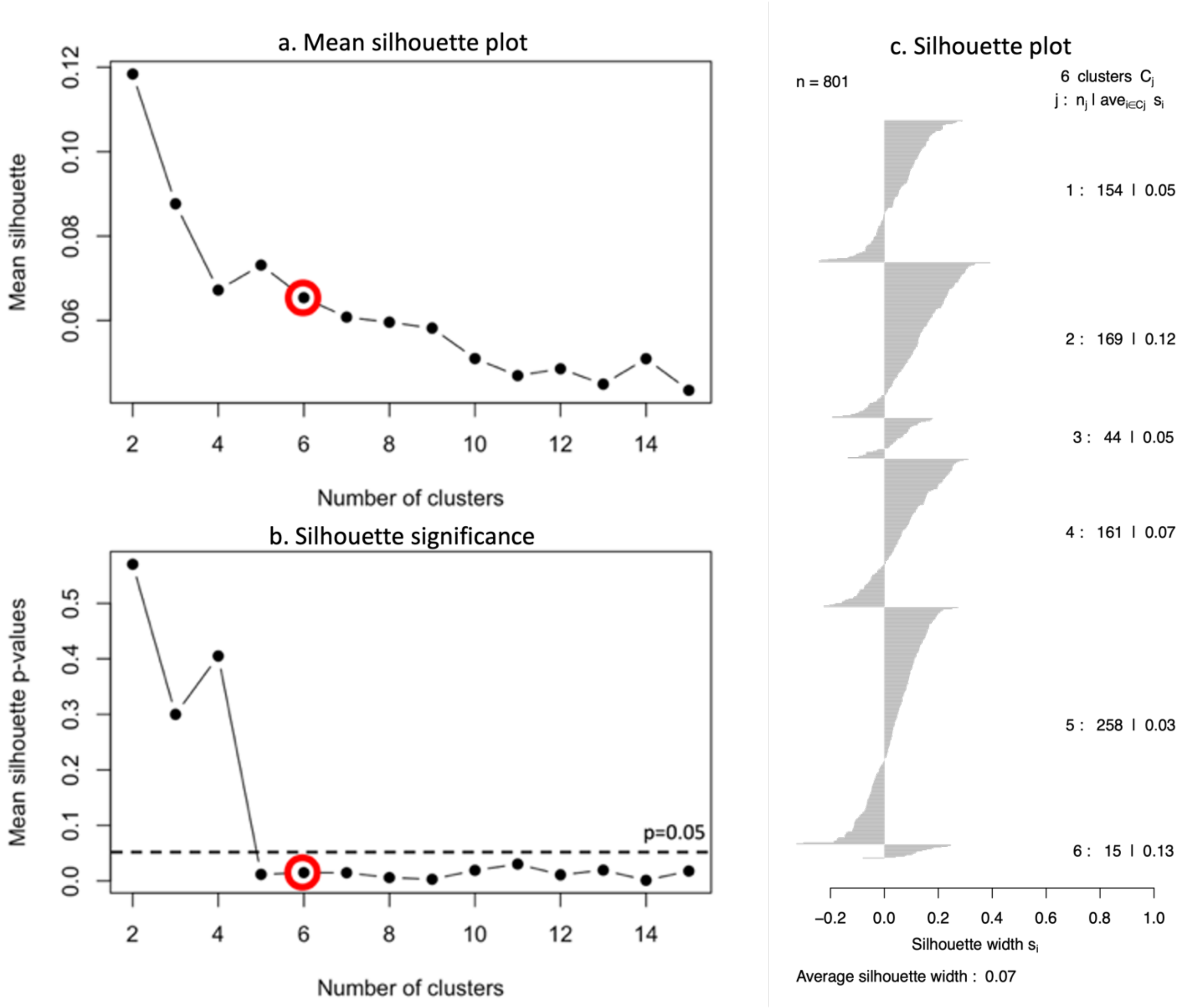

For different numbers of clusters, we show the mean silhouette (a) and its p-value defined as the fraction of mean silhouettes greater than our result obtained by clustering 10,000 synthetic datasets from a multivariate normal distribution (b). We selected 6 as the optimal number of clusters using four convergent sources of evidence: the elbow method, permutation-based significance testing of the silhouette index, stability using cross-validation; and the match of the solution to a theoretical framework (circled in red). The plot showing the silhouette values for each participant for the 6-cluster solution is shown (c).

Supplementary Figure 3: Permutation-based significance testing of the silhouette index.

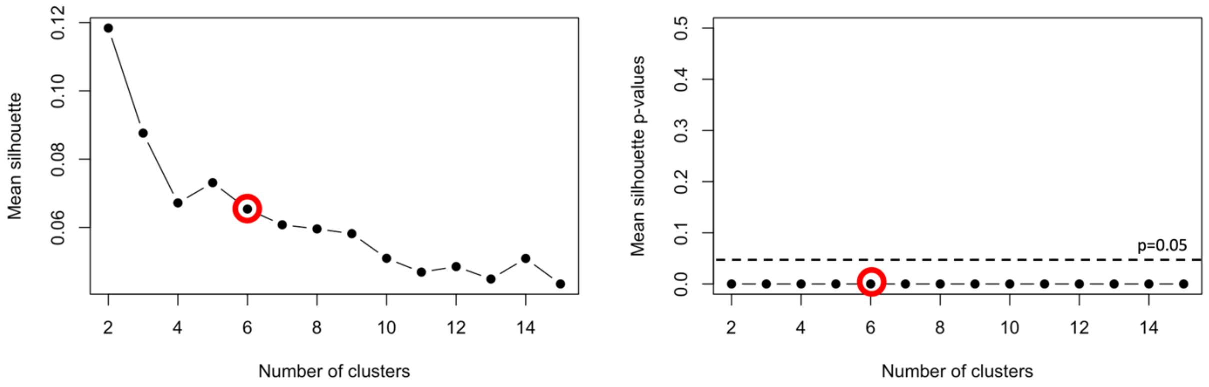

We shuffled each brain circuit score across subjects 10,000 times, then repeated the hierarchical clustering procedure and calculated the average silhouette index. Thus, we obtained null distributions for these average silhouette indexes, comprising 10,000 observations. We computed a p-value defined as the fraction of average silhouette indexes in this null distribution greater than our result. We selected 6 as the optimal number of clusters using six convergent sources of evidence: the elbow method, simulation-based significance testing of the silhouette index, permutation-based significance testing of the silhouette index, split-half reliability of the cluster profiles, stability using cross-validation; and the match of the solution to a theoretical framework (circled in red).

Supplementary Figure 4: Assessment of cluster stability using cross-validation.

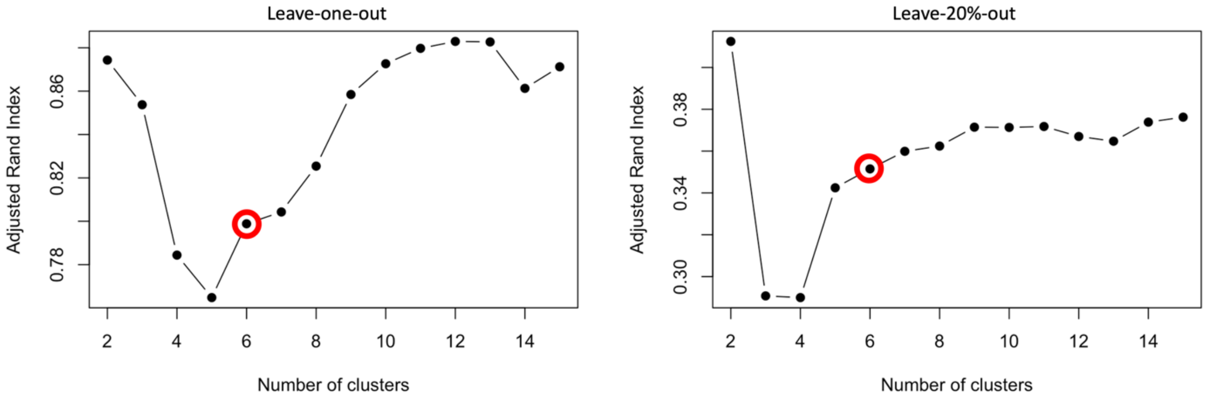

To evaluate whether the clustering assignment was stable under small perturbations to the data, we repeated the clustering procedure 801 times, each time with one participant left out (leave-one-out cross-validation, left). We also repeated the clustering procedure 801 times, each time with 20% of participants left out (leave-20%-out cross-validation, right). For each run and for each solution between 2 and 15 clusters, we calculated the similarity of the new cluster assignments to those from the original analysis using the adjusted Rand index. We selected 6 as the optimal number of clusters using six convergent sources of evidence: the elbow method, simulation-based significance testing of the silhouette index, permutation-based significance testing of the silhouette index, split-half reliability of the cluster profiles, stability using cross-validation; and the match of the solution to a theoretical framework (circled in red).

Supplementary Figure 5: Biotypes identified by hierarchical clustering.

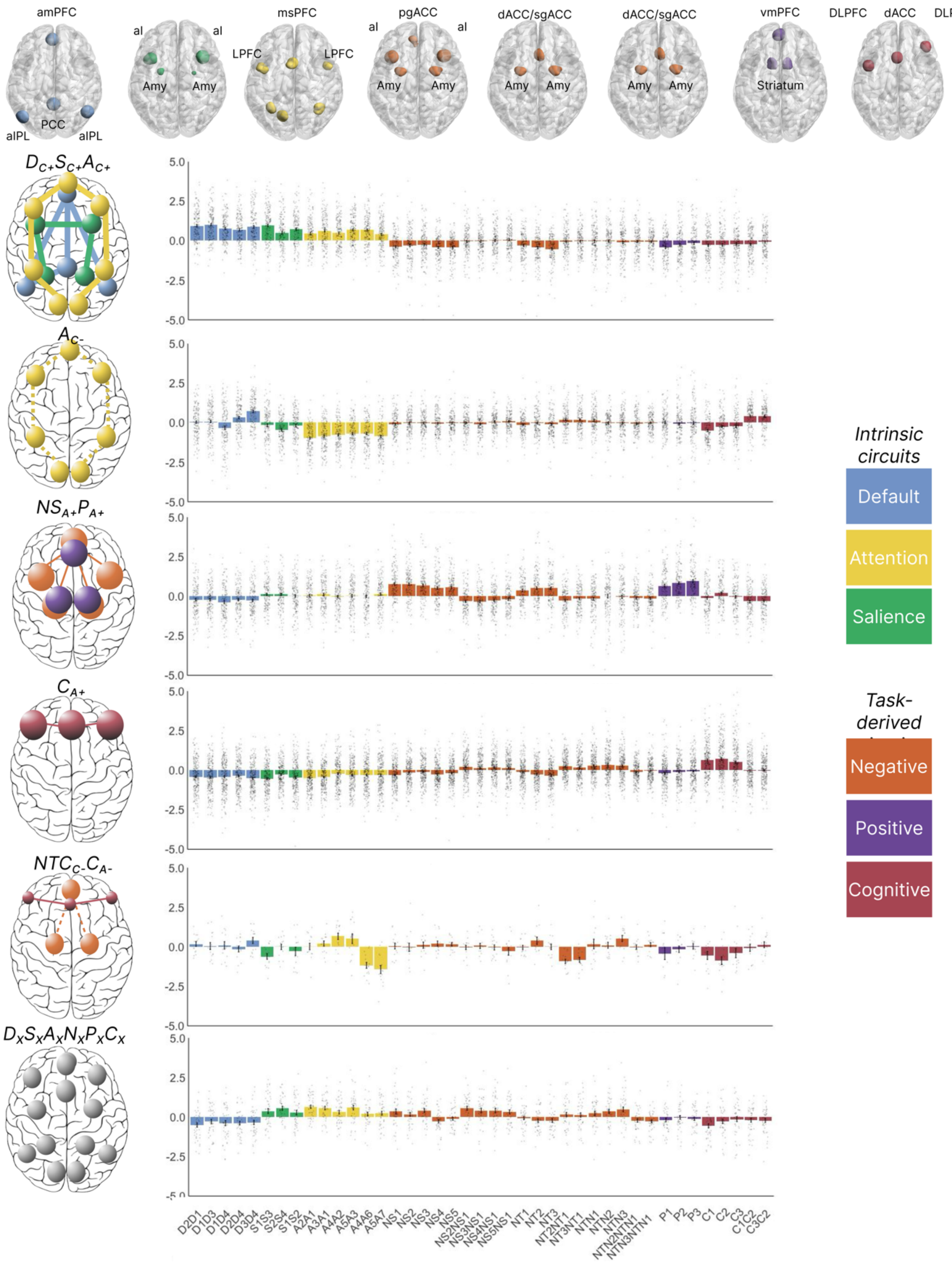

At the top, we show the regions of interest used to calculate regional circuit scores (see Supplementary Table 18 for details). Then, we show the average and standard error of regional circuit scores across participants. Colors correspond to each circuit. Measures are abbreviated as per Figure 1 in the main text. The size of spheres representing each circuit denotes an absolute activation difference of >0.50 SD compared to a healthy norm (small spheres=decreased activation, large spheres=increased activation). The thickness of lines between the spheres denotes an absolute connectivity difference of >0.50 SD compared to a healthy norm (dashed lines=decreased connectivity, thick lines=increased connectivity). We named each biotype according to the circuits and circuit features that specifically differentiated each relative to other biotypes and to the healthy reference. We used the following

nomenclature: each circuit is indicated with a letter (D = default mode, S = salience, A = attention, NS = negative affect circuit evoked by sad stimuli, NTC = negative affect circuit evoked by conscious threat stimuli, NTN = negative affect circuit evoked by non-conscious threat stimuli, P = positive circuit, C = cognitive circuit), the distinguishing circuit feature is indicated as a subscript (C = connectivity or A = activity) and the direction of dysfunction is indicated by + or -. The subscript x indicates that the sixth biotype is not differentiated by a prominent circuit dysfunction relative to other biotypes. Besides this nomenclature, we suggest a short plain-English description for each biotype (in quotes), that connects them with our theoretically synthesized biotypes: $D_{C+}S_{C+}A_{C+}$ = 'Default with salience and attention hyperconnectivity' (N=169 participants); A_{C-} = 'Attention hypoconnectivity' (N=161 participants); $NS_{A+}P_{A+}$ = 'Sad-elicited negative affect with positive affect hyperactivation'(N=154 participants); C_{A+} = 'Cognitive control hyperactivation'(N=258 participants); $NTC_{C-}C_{A-}$ = 'Cognitive control hypoactivation with conscious threat-elicited negative affect hypoconnectivity'(N=15 participants); $D_XS_XA_XN_XP_XC_X$ = 'Intact activation and connectivity' (N=44 participants). *Abbreviations:* AG=angular gyrus; aI=anterior insula; aIPL=anterior inferior parietal lobule, amPFC=anterior medial prefrontal cortex; Amy=amygdala; dACC=dorsal anterior cingulate cortex; DLPFC=dorsolateral prefrontal cortex; LPFC=lateral prefrontal cortex; msPFC=medial superior prefrontal cortex; PCC=posterior cingulate cortex; PCU=precuneus; pgACC=pregenual anterior cingulate cortex; sgACC=subgenual anterior cingulate cortex; vmPFC=ventero-medial prefrontal cortex.

Supplementary Figure 6: Split-half reliability of the biotype profiles.

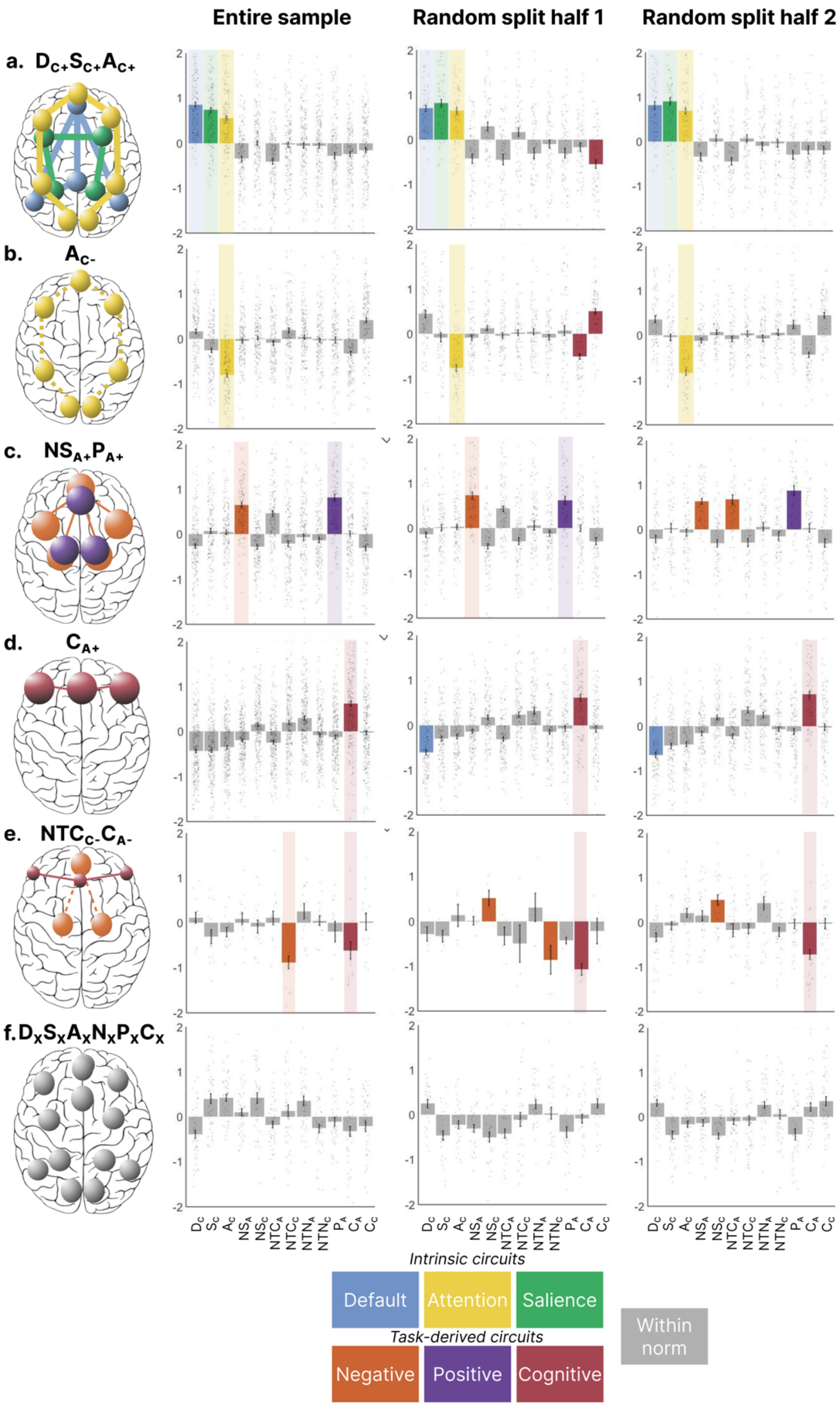

First, we split our dataset into two random samples of equal size. Then, we ran our clustering procedure on the first half-split. Then, we assigned each participant in the second split to one of the clusters obtained in the first half-split. To do so, we computed the mean circuit scores across all participants belonging to each cluster in the first half-split. Then, we calculated the Pearson correlation coefficient between each participant's brain circuit scores and these averaged scores. Each participant was assigned to the cluster for

which this correlation was highest. Finally, we identified the primary circuit dysfunctions of each cluster in each split as described above (>0.5 SD absolute mean difference compared to the healthy norm) and checked whether they replicated the ones found in the whole sample. We show the average and standard error of regional circuit scores across participants in the whole sample and in each of two random splits. All correlations of mean circuit profiles for each cluster between the two splits were significant ($D_{C+}S_{C+}A_{C+}$: r=0.95, two-sided p=1.72e-21; A_{C-} : r=0.97, two-sided p=7.08e-25; $NS_{A+}P_{A+}$: r=0.93, two-sided p=1.10e-18; C_{A+} : r=0.96, two-sided p=2.21e-23; $NTC_{C-}C_{A-}$: r=0.79, two-sided p=1.15e-09; $D_XS_XA_XN_XP_XC_X$: r=0.86, two-sided p=4.44e-13). We highlight with colored bands the primary circuit dysfunctions of the whole sample that replicated in the two half-splits (all but one). Colors correspond to each circuit. Measures are abbreviated as per Figure 1 in the main text. The size of spheres representing each circuit denotes an absolute activation difference of >0.50 SD compared to a healthy norm (small spheres=decreased activation, large spheres=increased activation). The thickness of lines between the spheres denotes an absolute connectivity difference of >0.50 SD compared to a healthy norm (dashed lines=decreased connectivity, thick lines=increased connectivity). We named each biotype according to the circuits and circuit features that specifically differentiated each relative to other biotypes and to the healthy reference. We used the following nomenclature: each circuit is indicated with a letter (D = default mode, S = salience, A = attention, NS = negative affect circuit evoked by sad stimuli, NTC = negative affect circuit evoked by conscious threat stimuli, NTN = negative affect circuit evoked by non-conscious threat stimuli, P = positive circuit, C = cognitive circuit), the distinguishing circuit feature is indicated as a subscript (C = connectivity or A = activity) and the direction of dysfunction is indicated by + or -. The subscript x indicates that the sixth biotype is not differentiated by a prominent circuit dysfunction relative to other biotypes. Besides this nomenclature, we suggest a short plain-English description for each biotype (in quotes), that connects them with our theoretically synthesized biotypes: $D_{C+}S_{C+}A_{C+}$ = 'Default with salience and attention hyperconnectivity' (N=169 participants); A_{C-} = 'Attention hypoconnectivity' (N=161 participants); $NS_{A+}P_{A+}$ = 'Sad-elicited negative affect with positive affect hyperactivation'(N=154 participants); C_{A+} = 'Cognitive control hyperactivation'(N=258 participants); $NTC_{C-}C_{A-}$ = 'Cognitive control hypoactivation with conscious threat-elicited negative affect hypoconnectivity'(N=15 participants); $D_XS_XA_XN_XP_XC_X$ = 'Intact activation and connectivity' (N=44 participants).

Supplementary Figure 7: Correlation between symptoms and regional circuit scores.

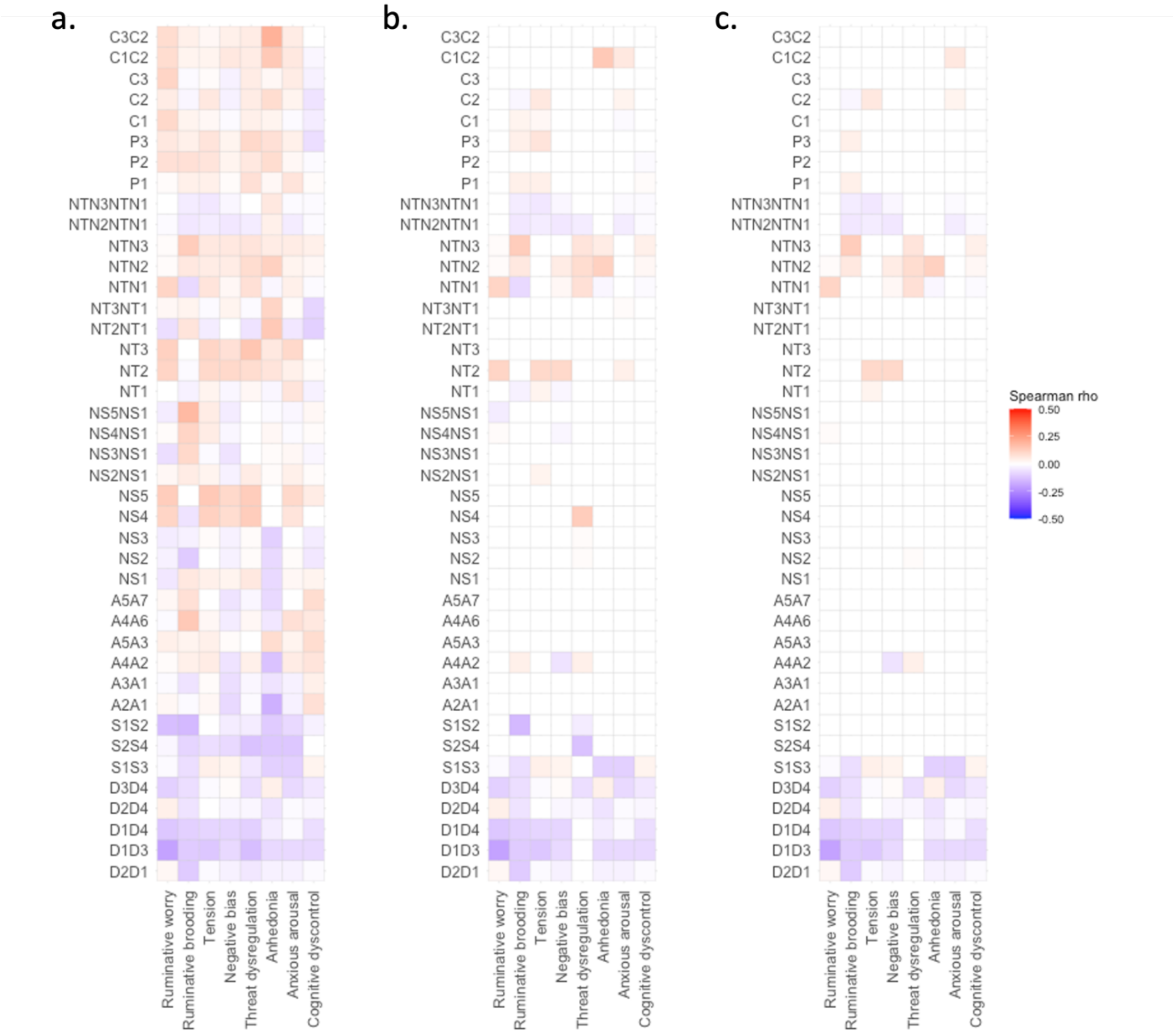

Across all clinical participants, we calculated a Spearman correlation between regional circuit scores and symptoms and show the resulting Spearman rho values as a heatmap, unthresholded (a), thresholded at two-sided p<0.05 (b) and thresholded with FDR correction at two-sided pFDR<0.05 (c). When thresholded at two-sided p<0.05, 127 (31%) correlations were significant and when thresholded with FDR correction, 86 (21%) correlations were significant. Of the correlations significant when thresholded with FDR correction 10 (2 %) correlations were of absolute magnitude rho>0.10 and the remaining 76 (19%) were of absolute magnitude rho<0.10. *Abbreviation:* FDR=false discovery rate.

Supplementary Figure 8: Correlation between behavioral performance and regional circuit scores.

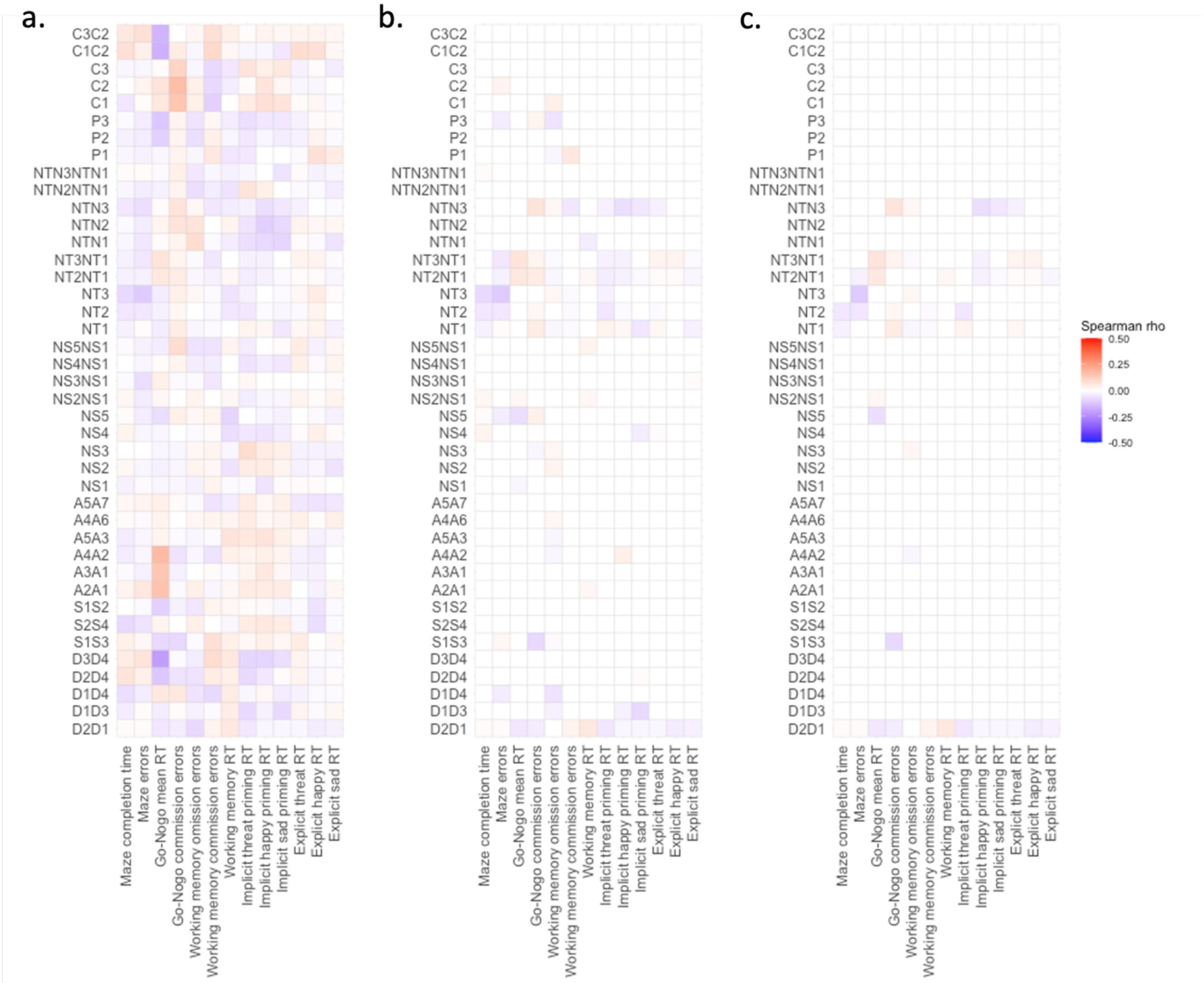

Across all clinical participants, we calculated a Spearman correlation between regional circuit scores and behavioral performance and show the resulting Spearman rho values as a heatmap, unthresholded (a), thresholded at two-sided p<0.05 (b) and thresholded with FDR correction at two-sided pFDR<0.05 (c). When thresholded at two-sided p<0.05, 131 (20%) correlations were significant and when thresholded with FDR correction, 66 (10%) correlations were significant. Of the correlations significant when thresholded with FDR correction 1 (0.15 %) correlation was of absolute magnitude rho>0.10 and the remaining 65 (10%) were of absolute magnitude rho<0.10. *Abbreviation:* FDR=false discovery rate.

Supplementary Figure 9: Correlation between treatment response and regional circuit scores.

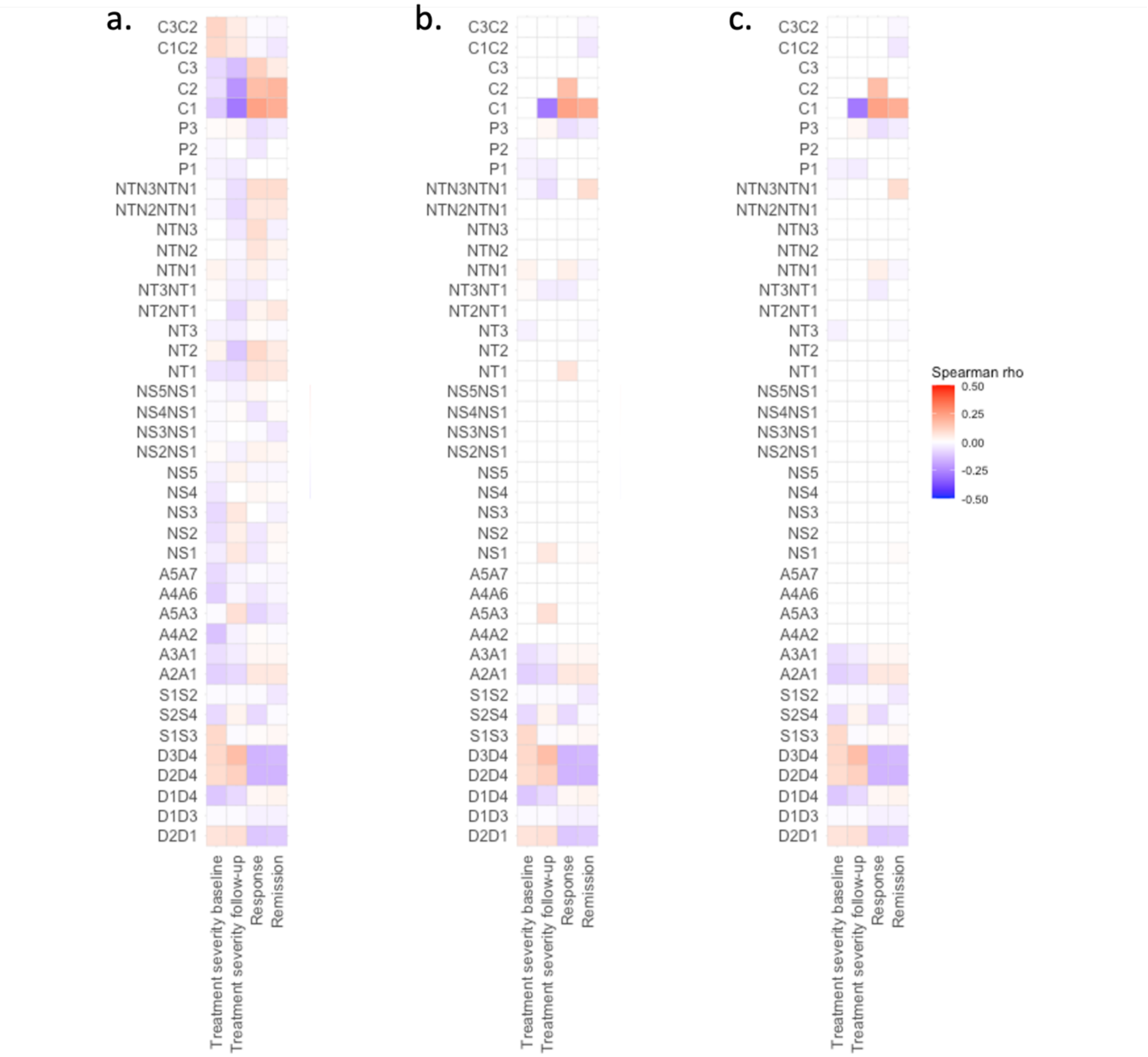

Across all clinical participants, we calculated a Spearman correlation between between regional circuit scores and treatment response and show the resulting Spearman rho values as a heatmap, unthresholded (a), thresholded at two-sided p<0.05 (b) and thresholded with FDR correction at two-sided pFDR<0.05 (c). When thresholded at two-sided p<0.05, 79 (39%) correlations were significant and when thresholded with FDR correction, 63 (31%) correlations were significant. Of the correlations significant when thresholded with FDR correction 15 (7%) correlations were of absolute magnitude rho>0.10 and the remaining 48 (23%) were of absolute magnitude rho<0.10. *Abbreviation:* FDR=false discovery rate.

Supplementary Figure 10: Between biotype comparisons for each symptom domain.

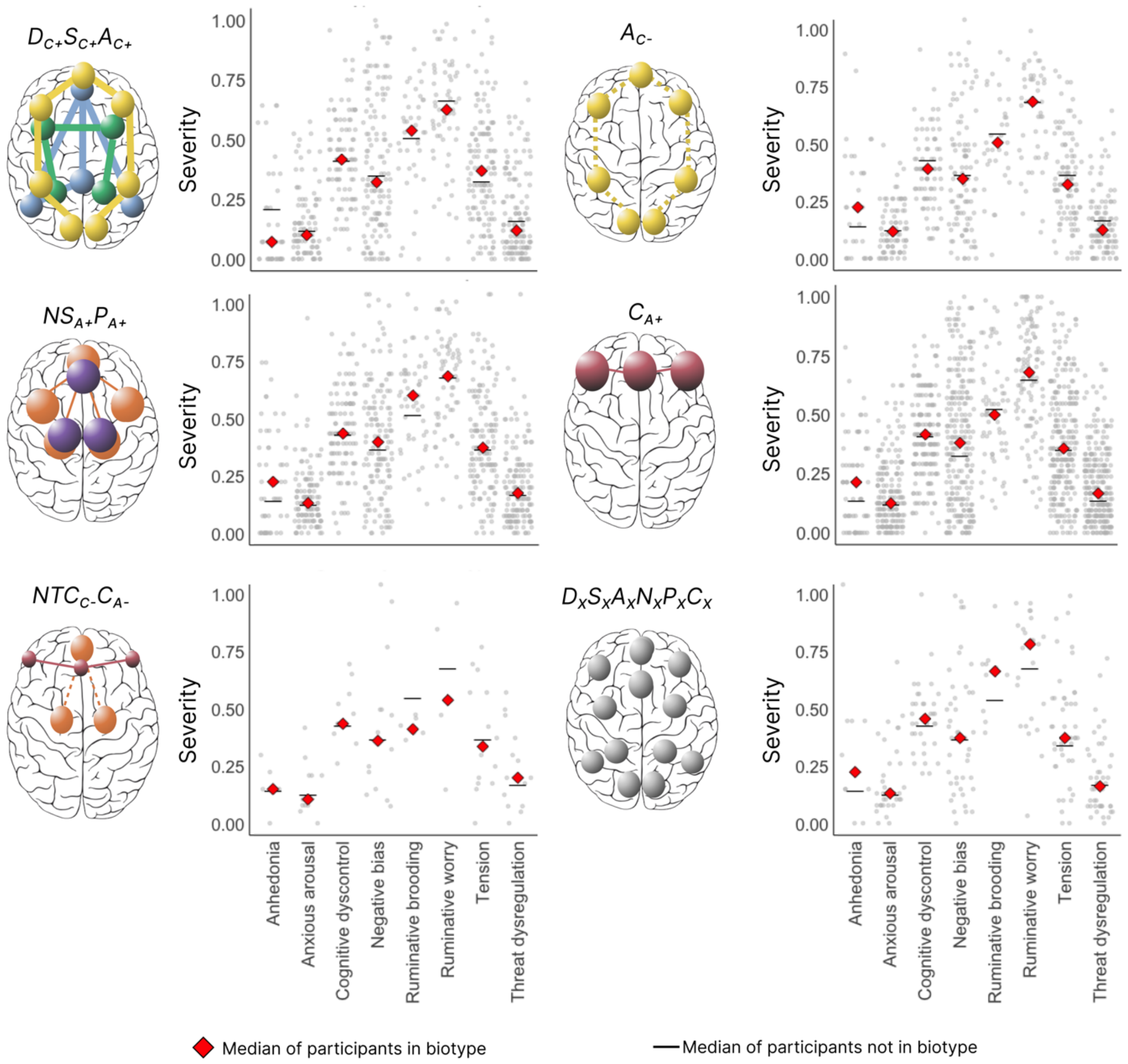

Plots comparing the severity of our symptoms of interest for participants in each biotype to that of participants not in the biotype. The dots show individual data points for individuals in the biotype. The median of participants in the biotype is shown as a red diamond, the median of participants not in the biotype is shown as a black line. To enable comparison across symptoms, all symptoms were scaled between 0 and 1 based on the minimum and maximum of the corresponding scales. We named each biotype according to the circuits and circuit features that specifically differentiated relative to each other biotypes and to the healthy reference. We used the following nomenclature: each circuit is indicated with a letter (D = default mode, S = salience, A = attention, NS = negative affect circuit evoked by sad stimuli, NTC = negative affect circuit evoked by conscious threat stimuli, NTN = negative affect circuit evoked by nonconscious threat stimuli, P = positive circuit, C = cognitive circuit), the distinguishing circuit feature is indicated as a subscript (C = connectivity or A = activity) and the direction of dysfunction is indicated by + or -. The subscript x indicates that the sixth biotype is not differentiated by a prominent circuit dysfunction relative to other biotypes. Besides this nomenclature, we suggest a short plain-English description for each biotype (in quotes), that connects them with our theoretically synthesized biotypes: $D_{C+}S_{C+}A_{C+}$ = 'Default with salience and attention hyperconnectivity' (N=169 participants); A_{C-} = 'Attention hypoconnectivity' (N=161 participants); $NS_{A+}P_{A+}$ = 'Sad-elicited negative affect with positive affect hyperactivation'(N=154 participants); C_{A+} = 'Cognitive control hyperactivation'(N=258 participants); $NTC_{C-}C_{A-}$ = 'Cognitive control hypoactivation with conscious threat-elicited negative affect hypoconnectivity'(N=15 participants); $D_XS_XA_XN_XP_XC_X$ = 'Intact activation and connectivity' (N=44 participants).

Supplementary Figure 11: Between biotype comparisons for insomnia and suicidality.

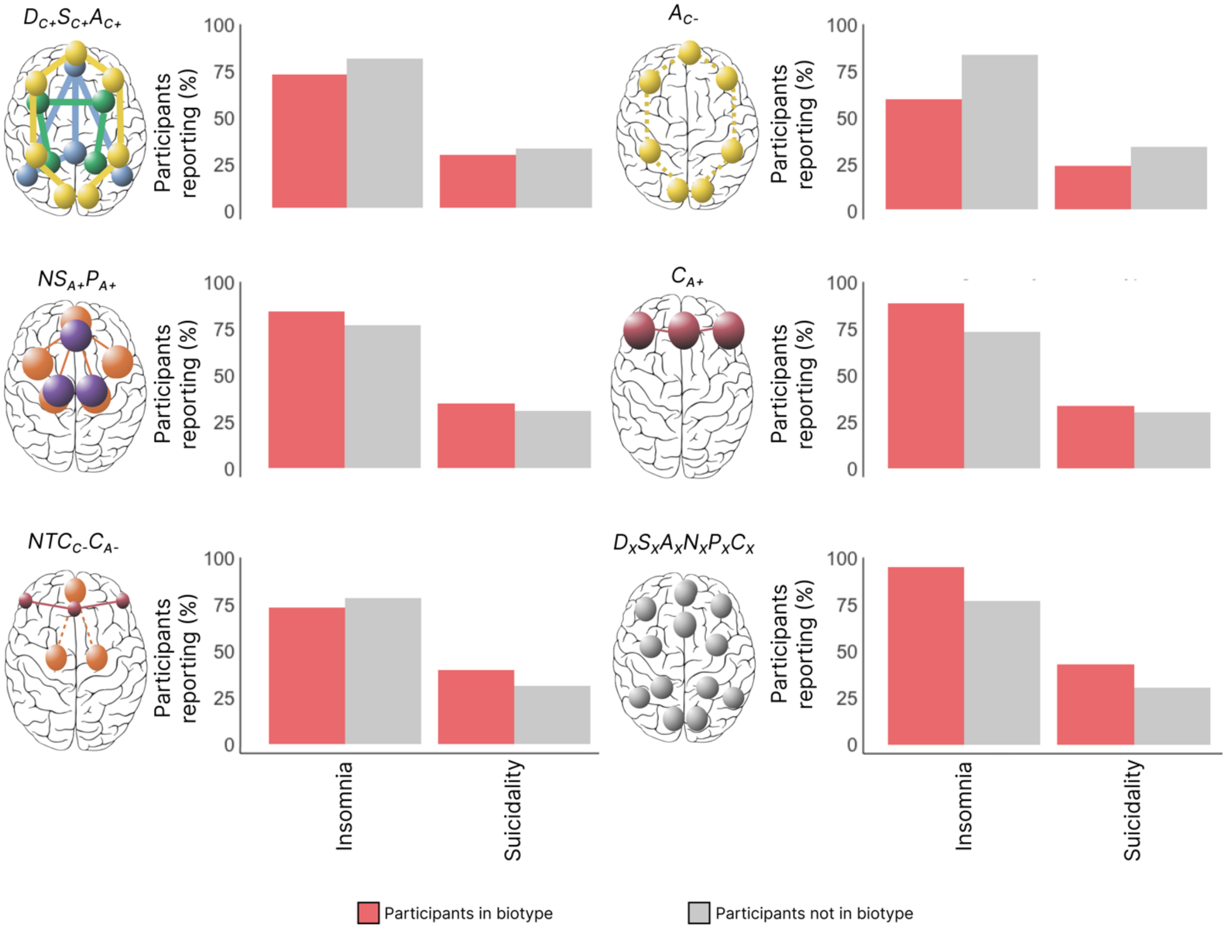

Insomnia was measured by the QIDS-SR sum of items 1-3 and suicidality was measured by the QIDS-SR item 12. A participant was considered as endorsing the symptom if their score was >0. We named each biotype according to the circuits and circuit features that specifically differentiated relative to each other biotypes and to the healthy reference. We used the following nomenclature: each circuit is indicated with a letter (D = default mode, S = salience, A = attention, NS = negative affect circuit evoked by sad stimuli, NTC = negative affect circuit evoked by conscious threat stimuli, NTN = negative affect circuit evoked by nonconscious threat stimuli, P = positive circuit, C = cognitive circuit), the distinguishing circuit feature is indicated as a subscript (C = connectivity or A = activity) and the direction of dysfunction is indicated by + or -. The subscript x indicates that the sixth biotype is not differentiated by a prominent circuit dysfunction relative to other biotypes. Besides this nomenclature, we suggest a short plain-English description for each biotype (in quotes), that connects them with our theoretically synthesized biotypes: $D_{C+}S_{C+}A_{C+}$ = 'Default with salience and attention hyperconnectivity'; A_{C-} = 'Attention hypoconnectivity'; $NS_{A+}P_{A+}$ = 'Sad-elicited negative affect with positive affect hyperactivation'; C_{A+} = 'Cognitive control hyperactivation'; $NTC_{C-}C_{A-}$ = 'Cognitive control hypoactivation with conscious threat-elicited negative affect hypoconnectivity'; $D_XS_XA_XN_XP_XC_X$ = 'Intact activation and connectivity'. *Abbreviation*: QIDS-SR= Quick Inventory of Depressive Symptomatology - Self-Report Revised.

Supplementary Figure 12: Between biotype comparisons of behavioral performance.

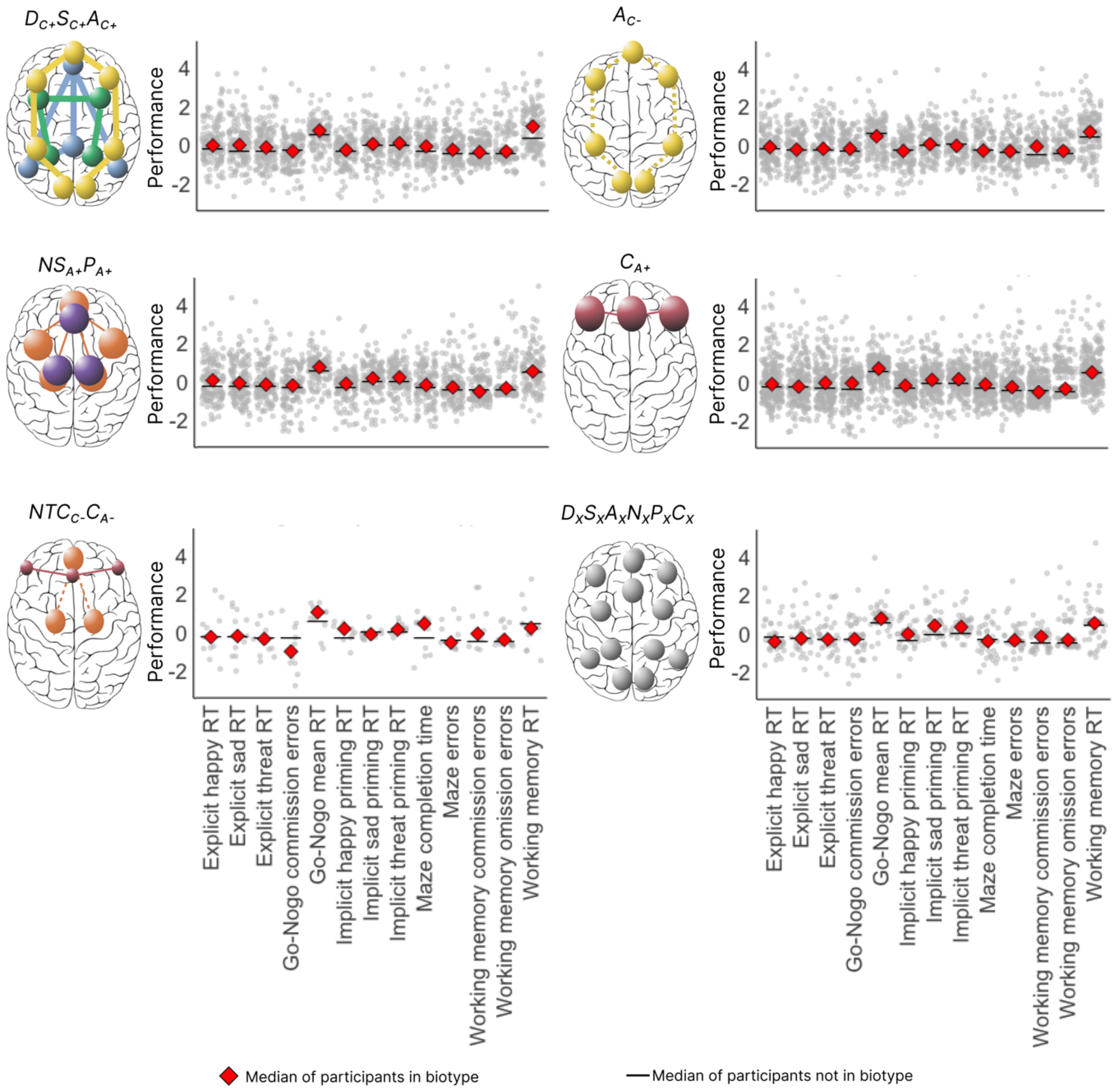

Plots comparing behavioral performance for participants in each biotype to that of participants not in the biotype. The dots show individual data points for individuals in the biotype. The median of participants in the biotype is shown as a red diamond, the median of participants not in the biotype is shown as a black line. Behavioral measures are adjusted for age and sex and are expressed relative to a healthy norm (Webneuro normed scores). We named each biotype according to the circuits and circuit features that specifically differentiated relative to each other biotypes and to the healthy reference. We used the following nomenclature: each circuit is indicated with a letter (D = default mode, S = salience, A = attention, NS = negative affect circuit evoked by sad stimuli, NTC = negative affect circuit evoked by conscious threat stimuli, NTN = negative affect circuit evoked by nonconscious threat stimuli, P = positive circuit, C = cognitive circuit), the distinguishing circuit feature is indicated as a subscript (C = connectivity, A = activity) and the direction of dysfunction is indicated by + or -. The subscript x indicates that the sixth biotype is not differentiated by a prominent circuit dysfunction relative to other biotypes. Besides this nomenclature, we suggest a short plain-English description for each biotype (in quotes), that connects them with our theoretically synthesized biotypes: $D_{C+}S_{C+}A_{C+}$ = 'Default with salience and attention hyperconnectivity' (N=169 participants); A_{C-} = 'Attention hypoconnectivity' (N=161 participants); $NS_{A+}P_{A+}$ = 'Sad-elicited negative affect with positive affect hyperactivation'(N=154 participants); C_{A+} = 'Cognitive control hyperactivation'(N=258 participants); $NTC_{C-}C_{A-}$ = 'Cognitive control hypoactivation with conscious threat-elicited negative affect hypoconnectivity'(N=15 participants); $D_XS_XA_XN_XP_XC_X$ = 'Intact activation and connectivity' (N=44 participants). *Abbreviation*: RT=reaction time.

Supplementary Figure 13: Between biotype comparisons of treatment outcomes.

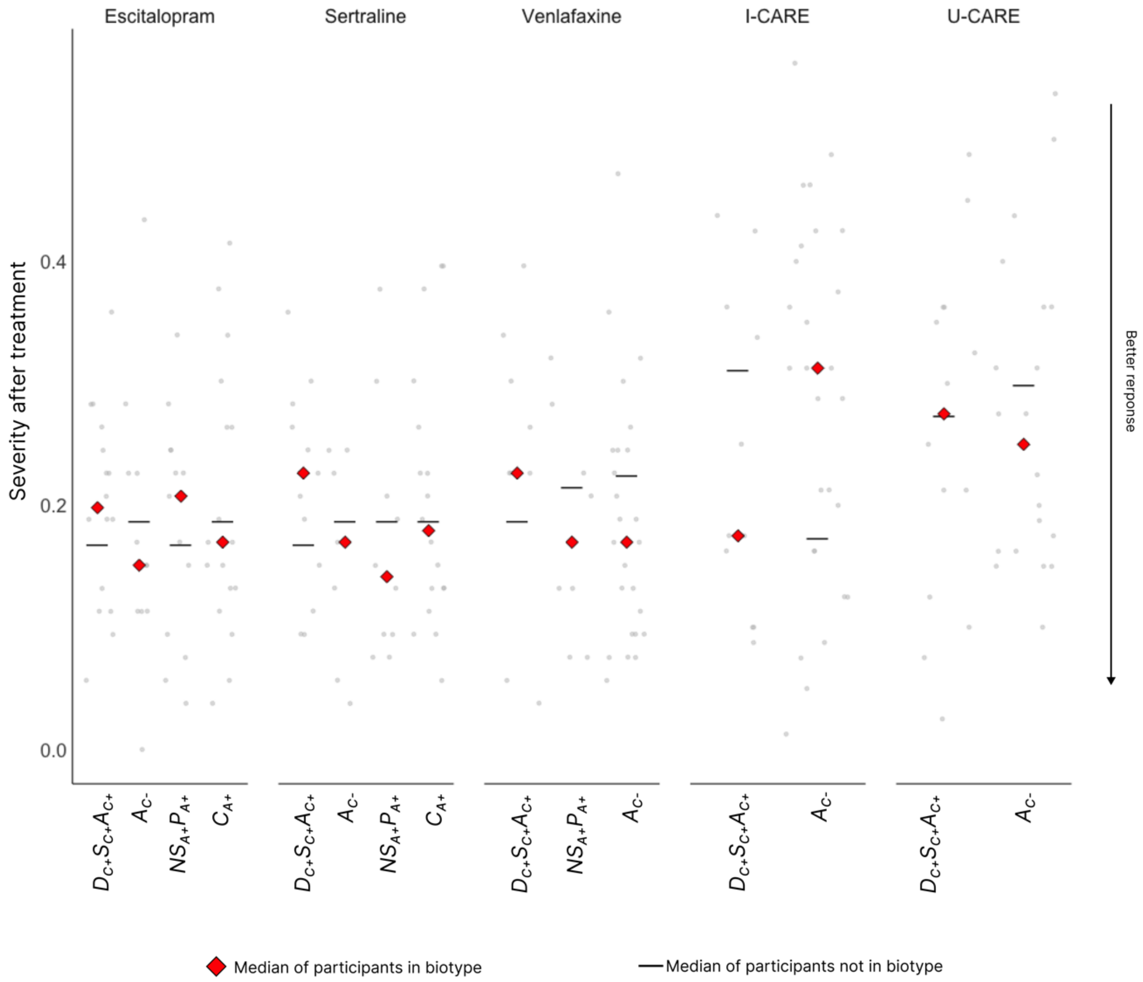

Plots comparing clinical severity after treatment for participants in each biotype to that of participants not in the biotype. The dots show individual data points for individuals in the biotype. The median of participants in the biotype is shown as a red diamond, the median of participants not in the biotype is shown as a black line. Severity is scaled between 0 and 1 based on the minimum and maximum of the symptom scales used. We named each biotype according to the circuits and circuit features that specifically differentiated relative to each other biotypes and to the healthy reference. We used the following nomenclature: each circuit is indicated with a letter (D = default mode, S = salience, A = attention, NS = negative affect circuit evoked by sad stimuli, NTC = negative affect circuit evoked by conscious threat stimuli, NTN = negative affect circuit evoked by non-conscious threat stimuli, P = positive circuit, C = cognitive circuit), the distinguishing circuit feature is indicated as a subscript (C = connectivity, A = activity) and the direction of dysfunction is indicated by + or -. The subscript x indicates that the sixth biotype is not differentiated by a prominent circuit dysfunction relative to other biotypes. Besides this nomenclature, we suggest a short plain-English description for each biotype (in quotes), that connects them with our theoretically synthesized biotypes: $D_{C+}S_{C+}A_{C+}$ = ‘Default with salience and attention hyperconnectivity’ (N=169 participants); A_{C-} = ‘Attention hypoconnectivity’ (N=161 participants); $NS_{A+}P_{A+}$ = ‘Sad-elicited negative affect with positive affect hyperactivation’(N=154 participants); C_{A+} = ‘Cognitive control hyperactivation’(N=258 participants); $NTC_{C-}C_{A-}$ = ‘Cognitive control hypoactivation with conscious threat-elicited negative affect hypoconnectivity’(N=15 participants); $D_XS_XA_XN_XP_XC_X$ = ‘Intact activation and connectivity’ (N=44 participants).

Supplementary Figure 14: Correlation between task-free and task regional circuit scores.

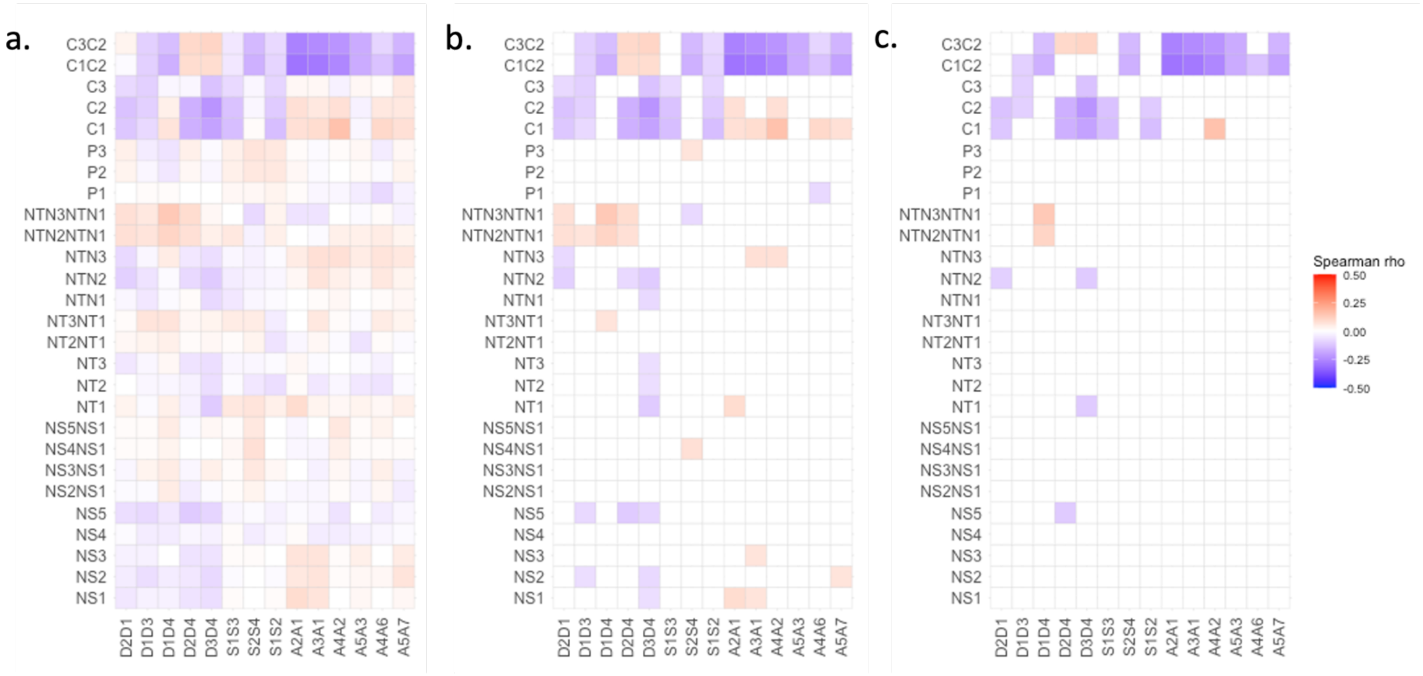

Across all clinical participants, we calculated a Spearman correlation between task-free and task regional circuit scores and show the resulting Spearman rho values as a heatmap, unthresholded (a), thresholded at two-sided p<0.05 (b) and thresholded with FDR correction at two-sided pFDR<0.05 (c). When thresholded at two-sided p<0.05, 110 (21%) correlations were significant and when thresholded with FDR correction, 53 (10%) correlations were significant. Of the correlations significant when thresholded with FDR correction 53 (10%) correlations were of absolute magnitude rho>0.10 and the remaining 0 (0%) were of absolute magnitude rho<0.10. *Abbreviation:* FDR=false discovery rate.

SUPPLEMENTARY TABLES

Supplementary Table 1: Demographics and diagnoses of the sample used in the cross-sectional analyses. DSM-IV-TR (RAD), DSM-5 (HCP-DES), or DSM-IV (iSPOT-D) criteria for major depressive disorder, anxiety disorder, post-traumatic stress disorder or obsessive-compulsive disorder were ascertained by a psychiatrist, general practitioner or research personnel using the structured interview, Mini International Neuropsychiatric Interview (MINI) (Sheehan et al., 1998). In the ENGAGE sample, patients were considered eligible if they scored 10 or greater on PHQ-9, a threshold with 88% specificity for major depressive disorder (Kroenke et al., 2001), and had a qualifying BMI at study screening. Comorbidities were ascertained from electronic health records. *Abbreviations:* ENGAGE=Engaging self-regulation targets to understand the mechanisms of behavior change and improve mood and weight outcome; HCP-DES=Human Connectome Project for Disordered Emotional States; iSPOT-D=International Study to Predict Optimized Treatment in Depression; QIDS-SR=Quick Inventory of Depressive Symptomatology Self-Report Revised; RAD=Research on Anxiety and Depression study; SCL-20=Symptom Checklist 20 depression scale. A dash indicates that the information was not available in the dataset.

Features	Clinical Sample 1	Clinical Sample 2	Clinical Sample 3	Clinical Sample 4	Controls Sample 1	Control Sample 2
Dataset	RAD	HCP-DES	iSPOT-D	ENGAGE	HCP-DES	iSPOT-D
N	309	208	198	86	70	67
Gender						
Female	178 (58%)	126 (61%)	100 (51%)	57 (66%)	33 (47%)	34 (51%)
Male	127 (41%)	75 (36%)	98 (49%)	29 (34%)	37 (53%)	33 (49%)
Other	4 (1%)	7 (3%)	0 (0%)	0 (0%)	0 (0%)	0 (0%)
Age mean (standard deviation)	35.12 (14.04)	26.11 (4.91)	34.21 (11.97)	50.81 (11.98)	33.44 (12.12)	30.71 (12.97)
Race						
American Indian/Alaska native	1	2 (1%)	0 (0%)	0 (0%)	0 (0%)	0 (0%)
Asian	65	75 (36%)	35 (18%)	6 (7%)	20 (29%)	9 (13%)
Black/African American	8	5 (2%)	2 (1%)	1 (1%)	1 (1%)	0 (0%)
Hawaiian/pacific islander	0	0 (0%)	0 (0%)	1 (1%)	0 (0%)	0 (0%)
More than one race	1	23 (11%)	0 (0%)	7 (8%)	4 (6%)	0 (0%)
Other	48	15 (7%)	38 (19%)	2 (2%)	5 (7%)	1 (1%)
White	186	88 (42%)	122 (62%)	66 (80%)	40 (57%)	57 (85%)
Currently taking psychiatric medication	13 (4%)	0 (0%)	0 (0%)	27 (31%)	0 (0%)	0 (0%)
Current diagnoses						
Major depressive disorder/depression	27 (9%)	64 (31%)	198 (100%)	86 (100%)	0 (0%)	0 (0%)
Generalized anxiety disorder	92 (32%)	89 (43%)	11 (6%)	0 (0%)	0 (0%)	0 (0%)
Panic disorder	19 (7%)	26 (12%)	22 (11%)	8 (10%)	0 (0%)	0 (0%)
Social anxiety disorder	35 (12%)	65 (31%)	79 (40%)	0 (0%)	0 (0%)	0 (0%)
Obsessive-compulsive disorder	20 (9%)	22 (8%)	0 (0%)	0 (0%)	0 (0%)	0 (0%)
Post-traumatic stress disorder	19 (9%)	18 (11%)	0 (0%)	0 (0%)	0 (0%)	0 (0%)
Severity						
QIDS-SR mean (standard deviation)	10 (4.81)	10.8 (4.75)	14 (3.72)	-	3.01 (2.44)	2.16 (1.61)
SCL-20 mean (standard deviation)	-	-	-	1.58 (0.54)	-	-
Scanner						
GE Signa HDx 3T	0 (0%)	0 (0%)	198 (100%)	0 (0%)	0 (0%)	67 (100%)
GE MR750 Discovery 3T	309 (100%)	120 (58%)	0 (0%)	86 (100%)	70 (100%)	0 (0%)
GE UHP 3T	0 (0%)	88 (42%)	0 (0%)	0 (0%)	0 (0%)	0 (0%)

Supplementary Table 2: Demographics and diagnoses of the sample used in the treatment analyses.
Abbreviations: ENGAGE=Engaging self-regulation targets to understand the mechanisms of behavior change and improve mood and weight outcome; iSPOT-D=International Study to Predict Optimized Treatment in Depression; QIDS-SR=Quick Inventory of Depressive Symptomatology Self-Report Revised; SCL-20=Symptom Checklist 20 depression scale; I-CARE=active behavioral therapy; U-CARE=usual care. A dash indicates that the information was not available in the dataset.

Features	Escitalopram treatment	Sertraline treatment	Venlafaxine treatment	U-CARE treatment	I-CARE treatment
Dataset	iSPOT-D	iSPOT-D	iSPOT-D	ENGAGE	ENGAGE
N	59	55	50	40	46
Gender					
Female	29 (49%)	23 (42%)	31 (62%)	25 (62%)	32 (70%)
Male	30 (51%)	32 (58%)	19 (38%)	15 (38%)	14 (30%)
Other	0 (0%)	0 (0%)	0 (0%)	0 (0%)	0 (0%)
Age mean (standard deviation)	34.42 (11.67)	32.65 (11.12)	34.9 (12.45)	50.37 (12.44)	51.19 (11.69)
Race					
American Indian/Alaska native	0 (0%)	0 (0%)	0 (0%)	0 (0%)	0 (0%)
Asian	13 (22%)	9 (16%)	7 (14%)	2 (5%)	4 (9%)
Black/African American	2 (3%)	0 (0%)	0 (0%)	0 (0%)	0 (0%)
Hawaiian/pacific islander	0 (0%)	0 (0%)	0 (0%)	0 (0%)	1 (2%)
More than one race	0 (0%)	0 (0%)	0 (0%)	0 (0%)	0 (0%)
Other	13 (22%)	13 (24%)	9 (18%)	1 (3%)	1 (2%)
White	30 (52%)	33 (60%)	34 (68%)	30 (77%)	36 (82%)
Currently taking psychiatric medication	0 (0%)	0 (0%)	0 (0%)	13 (32%)	14 (30%)
Current diagnoses					
Major depressive disorder	59 (100%)	55 (100%)	50 (100%)	40 (100%)	46 (100%)
Generalized anxiety disorder	2 (3%)	1 (2%)	3 (6%)	0 (0%)	0 (0%)
Panic disorder	7 (12%)	5 (9%)	4 (8%)	3 (8%)	5 (11%)
Social anxiety disorder	23 (39%)	21 (38%)	22 (44%)	-	-
Obsessive-compulsive disorder	0 (0%)	0 (0%)	0 (0%)	-	-
Post-traumatic stress disorder	0 (0%)	0 (0%)	0 (0%)	-	-
Severity					
QIDS-SR mean (standard deviation)	14.4 (4.23)	14.1 (3.65)	13.8 (3.34)	-	-
SCL-20 mean (standard deviation)	-	-	-	1.65 (0.52)	1.52 (0.56)
Scanner					
GE Signa HDx 3T	59 (100%)	55 (100%)	50 (100%)	0 (0%)	0 (0%)
GE MR750 Discovery 3T	0 (0%)	0 (0%)	0 (0%)	40 (100%)	46 (100%)
GE UHP 3T	0 (0%)	0 (0%)	0 (0%)	0 (0%)	0 (0%)

Supplementary Table 3: Significant between biotype comparisons for each symptom domain.

For each symptom, the scores of participants in each biotype were compared to the median of participants not in the biotype using a Mann-Whitney U test. Here, we show the direction of the difference (↑ =symptom median was higher in the biotype, ↓ =symptom median was lower in the biotype), number of participants used for this comparison in each biotype, the median, the two-sided p-value of the test, a measure of effect size r, calculated as the Z statistic divided by square root of the sample size and confidence interval (CI). We also show whether the finding replicates in split-half and leave-study-out procedures. We named each biotype according to the circuits and circuit features that specifically differentiated each relative to other biotypes and to the healthy reference. We used the following nomenclature: each circuit is indicated with a letter (D = default mode, S = salience, A = attention, NS = negative affect circuit evoked by sad stimuli, NTC = negative affect circuit evoked by conscious threat stimuli, NTN = negative affect circuit evoked by non-conscious threat stimuli, P = positive circuit, C = cognitive circuit), the distinguishing circuit feature is indicated as a subscript (C = connectivity or A = activity) and the direction of dysfunction is indicated by + or -. The subscript x indicates that the sixth biotype is not differentiated by a prominent circuit dysfunction relative to other biotypes. Besides this nomenclature, we suggest a short plain-English description for each biotype (in quotes), that connects them with our theoretically synthesized biotypes: $D_{C+}S_{C+}A_{C+}$ = 'Default with salience and attention hyperconnectivity'; A_{C-} = 'Attention hypoconnectivity'; $NS_{A+}P_{A+}$ = 'Sad-elicited negative affect with positive affect hyperactivation'; C_{A+} = 'Cognitive control hyperactivation'; $NTC_{C-}C_{A-}$ = 'Cognitive control hypoactivation with conscious threat-elicited negative affect hypoconnectivity'; $D_XS_XA_XN_XP_XC_X$ = 'Intact activation and connectivity'. *Abbreviation*: CI = confidence interval.

Biotype	Symptom	Direction	N	Median	p	Effect size r	CI	Split-half	Leave-study-out
**A_{C-}**	Cognitive dyscontrol	↓	80	17	0.006	-0.305	[15.5; 17.5]	No	No
	Tension	↓	101	13	0.049	-0.196	[11.5; 15.0]	No	No
**$NS_{A+}P_{A+}$**	Anhedonia	↑	51	3	0.014	0.343	[2.0; 4.5]	No	No
	Ruminative brooding	↑	51	60	0.036	0.294	[55.5; 63.0]	No	No
**C_{A+}**	Anhedonia	↑	68	3	0.015	0.295	[2.0; 3.5]	No	No
	Anxious arousal	↑	182	15	0.003	0.218	[15.5; 17.5]	No	No
	Negative bias	↑	250	16	0.003	0.188	[15.0; 18.5]	Yes	No
	Threat dysregulation	↑	250	7	5.07e-07	0.317	[7.5; 9.0]	Yes	Yes
**$NTC_{C-}C_{A-}$**	Ruminative brooding	↓	6	48	0.036	-0.902	[46.0; 51.0]	No	No

Supplementary Table 4: Between biotype comparisons for each symptom domain.

For each symptom, the scores of participants in each biotype were compared to the median of participants not in the biotype using a Mann-Whitney U test. Here, we show the direction of the difference (↑ =symptom median was higher in the biotype, ↓ =symptom median was lower in the biotype), number of participants used for this comparison in each biotype, the median, the two-sided p-value of the test, a measure of effect size r, calculated as the Z statistic divided by square root of the sample size and confidence interval (CI). We named each biotype according to the circuits and circuit features that specifically differentiated each relative to other biotypes and to the healthy reference. We used the following nomenclature: each circuit is indicated with a letter (D = default mode, S = salience, A = attention, NS = negative affect circuit evoked by sad stimuli, NTC = negative affect circuit evoked by conscious threat stimuli, NTN = negative affect circuit evoked by non-conscious threat stimuli, P = positive circuit, C = cognitive circuit), the distinguishing circuit feature is indicated as a subscript (C = connectivity or A = activity) and the direction of dysfunction is indicated by + or -. The subscript x indicates that the sixth biotype is not differentiated by a prominent circuit dysfunction relative to other biotypes. Besides this nomenclature, we suggest a short plain-English description for each biotype (in quotes), that connects them with our theoretically synthesized biotypes: $D_{C+}S_{C+}A_{C+}$ = 'Default with salience and attention hyperconnectivity'; A_{C-} = 'Attention hypoconnectivity'; $NS_{A+}P_{A+}$ = 'Sad-elicited negative affect with positive affect hyperactivation'; C_{A+} = 'Cognitive control hyperactivation'; $NTC_{C-}C_{A-}$ = 'Cognitive control hypoactivation with conscious threat-elicited negative affect hypoconnectivity'; $D_XS_XA_XN_XP_XC_X$ = 'Intact activation and connectivity'. *Abbreviation*: CI = confidence interval.

Biotype	Symptom	Direction	N	Median	p	Effect size r	CI
$D_{C+}S_{C+}A_{C+}$	Anhedonia	↓	40	1	0.058	-0.300	[0.5; 3.0]
	Anxious arousal	↑	95	14	0.404	0.086	[14.5; 17.0]
	Cognitive dyscontrol	↑	95	18	0.672	0.044	[17.0; 19.5]
	Negative bias	↑	134	13.5	0.384	0.075	[13.5; 18.5]
	Ruminative brooding	↑	40	57.5	0.652	0.072	[52.0; 61.0]
	Ruminative worry	↓	55	56	0.132	-0.204	[51.5; 60.0]
	Tension	↑	132	15.5	0.081	0.151	[14.0; 17.5]
	Threat dysregulation	↑	132	5	0.645	0.040	[5.5; 9.0]
**A_{C-}**	Anhedonia	↑	33	3	0.077	0.310	[2.0; 4.5]
	Anxious arousal	↑	80	14.5	0.799	0.029	[14.0; 16.0]
	Cognitive dyscontrol	↓	80	17	0.006	-0.305	[15.5; 17.5]
	Negative bias	↓	101	14	0.734	-0.034	[12.0; 17.0]
	Ruminative brooding	↓	33	54	0.300	-0.183	[50.0; 59.5]
	Ruminative worry	↓	47	58	0.108	-0.235	[51.0; 59.0]
	Tension	↓	101	13	0.049	-0.196	[11.5; 15.0]
	Threat dysregulation	↓	101	5	0.051	-0.195	[4.5; 7.0]
$NS_{A+}P_{A+}$	Anhedonia	↑	51	3	0.014	0.343	[2.0; 4.5]
	Anxious arousal	↑	114	15	0.194	0.122	[14.5; 16.5]
	Cognitive dyscontrol	↑	114	18	0.635	0.045	[17.0; 19.0]
	Negative bias	↑	143	16	0.335	0.081	[14.0; 18.0]
	Ruminative brooding	↑	51	60	0.036	0.294	[55.5; 63.0]
	Ruminative worry	↓	63	58	0.332	-0.123	[52.5; 60.0]
	Tension	↑	142	15	0.317	0.084	[14.0; 17.5]
	Threat dysregulation	↑	145	7	0.311	0.084	[6.5; 9.0]
C_{A+}	Anhedonia	↑	68	3	0.015	0.295	[2.0; 3.5]
	Anxious arousal	↑	182	15	0.003	0.218	[15.5; 17.5]
	Cognitive dyscontrol	↓	182	18	0.751	-0.024	[17.0; 18.5]
	Negative bias	↑	250	16	0.003	0.188	[15.0; 18.5]
	Ruminative brooding	↓	68	55	0.428	-0.096	[52.5; 59.0]
	Ruminative worry	↑	114	59.5	0.631	0.045	[55.5; 61.5]
	Tension	↑	251	15	0.245	0.073	[14.5; 17.0]

	Threat dysregulation	↑	250	7	5.07e-07	0.317	[7.5; 9.0]
$NTC_{C-}C_{A-}$	Anhedonia	↑	6	2	1.000	0.111	[0.0; 4.0]
	Anxious arousal	↑	11	14	0.531	0.202	[13.0; 19.5]
	Cognitive dyscontrol	↑	11	18	0.362	0.296	[16.0; 22.5]
	Negative bias	↑	14	14.5	1.000	0.008	[9.0; 25.0]
	Ruminative brooding	↓	6	48	0.036	-0.902	[46.0; 51.0]
	Ruminative worry	↓	5	49	0.813	-0.181	[25.0; 75.0]
	Tension	↑	14	13.5	0.889	0.047	[8.0; 22.5]
	Threat dysregulation	↑	14	8	0.278	0.299	[5.0; 15.0]
$D_XS_XA_XN_XP_XC_X$	Anhedonia	↑	10	3	0.103	0.541	[2.0; 10.0]
	Anxious arousal	↑	34	15	0.197	0.223	[14.0; 20.0]
	Cognitive dyscontrol	↑	34	18.5	0.536	0.108	[17.0; 20.0]
	Negative bias	↑	44	15	0.856	0.028	[12.0; 20.0]
	Ruminative brooding	↑	10	64	0.101	0.534	[55.0; 67.5]
	Ruminative worry	↑	25	64	1.000	0.003	[52.5; 65.5]
	Tension	↑	44	15	0.126	0.232	[13.5; 20.0]
	Threat dysregulation	↑	44	6.5	0.665	0.066	[5.5; 9.5]

Supplementary Table 5: Between biotype comparisons for insomnia and suicidality.

Since insomnia and suicidality were assessed using only three and one item on the QIDS-SR respectively, we instead used a chi-square test comparing the fraction of participants in the biotype endorsing any of the items (total value >0) compared to those not in the biotype. Here we show number of participants used for this comparison in each biotype, the percentage of participants reporting the symptom in the biotype and in other biotypes, the value and two-sided p-value of the test. We named each biotype according to the circuits and circuit features that specifically differentiated each relative to other biotypes and to the healthy reference. We used the following nomenclature: each circuit is indicated with a letter (D = default mode, S = salience, A = attention, NS = negative affect circuit evoked by sad stimuli, NTC = negative affect circuit evoked by conscious threat stimuli, NTN = negative affect circuit evoked by non-conscious threat stimuli, P = positive circuit, C = cognitive circuit), the distinguishing circuit feature is indicated as a subscript (C = connectivity or A = activity) and the direction of dysfunction is indicated by + or -. The subscript x indicates that the sixth biotype is not differentiated by a prominent circuit dysfunction relative to other biotypes. Besides this nomenclature, we suggest a short plain-English description for each biotype (in quotes), that connects them with our theoretically synthesized biotypes: $D_{C+}S_{C+}A_{C+}$ = 'Default with salience and attention hyperconnectivity'; A_{C-} = 'Attention hypoconnectivity'; $NS_{A+}P_{A+}$ = 'Sad-elicited negative affect with positive affect hyperactivation'; C_{A+} = 'Cognitive control hyperactivation'; $NTC_{C-}C_{A-}$ = 'Cognitive control hypoactivation with conscious threat-elicited negative affect hypoconnectivity'; $D_XS_XA_XN_XP_XC_X$ = 'Intact activation and connectivity'.

Biotype	Symptom	N	Participants reporting (biotype)	Participants reporting (other)	p	Chi-square
$D_{C+}S_{C+}A_{C+}$	Insomnia	169	72%	80%	0.930	0.008
	Suicidality	169	28%	32%	0.979	0.001
A_{C-}	Insomnia	161	59%	83%	0.567	0.327
	Suicidality	161	23%	33%	1.000	0.000
$NS_{A+}P_{A+}$	Insomnia	154	84%	77%	0.492	0.473
	Suicidality	154	34%	30%	1.000	0.000
C_{A+}	Insomnia	258	88%	73%	0.959	0.003
	Suicidality	258	34%	30%	0.590	0.291
$NTC_{C-}C_{A-}$	Insomnia	15	73%	78%	0.306	1.050
	Suicidality	15	40%	31%	0.797	0.066
$D_XS_XA_XN_XP_XC_X$	Insomnia	44	95%	77%	0.343	0.901
	Suicidality	44	43%	31%	0.390	0.739

Supplementary Table 6: Significant between biotype comparisons of behavioral performance.

For each behavioral measure, the scores of participants in each biotype were compared to the median of participants not in the biotype using a Mann-Whitney U test. Here, we show the direction of the difference (↑ =symptom median was higher in the biotype, ↓ =symptom median was lower in the biotype), number of participants used for this comparison in each biotype, the median, the two-sided p-value of the test, a measure of effect size r, calculated as the Z statistic divided by square root of the sample size and confidence interval (CI). We also show whether the finding replicates in split-half and leave-study-out procedures. We named each biotype according to the circuits and circuit features that specifically differentiated each relative to other biotypes and to the healthy reference. We used the following nomenclature: each circuit is indicated with a letter (D = default mode, S = salience, A = attention, NS = negative affect circuit evoked by sad stimuli, NTC = negative affect circuit evoked by conscious threat stimuli, NTN = negative affect circuit evoked by non-conscious threat stimuli, P = positive circuit, C = cognitive circuit), the distinguishing circuit feature is indicated as a subscript (C = connectivity or A = activity) and the direction of dysfunction is indicated by + or -. The subscript x indicates that the sixth biotype is not differentiated by a prominent circuit dysfunction relative to other biotypes. Besides this nomenclature, we suggest a short plain-English description for each biotype (in quotes), that connects them with our theoretically synthesized biotypes: $D_{C+}S_{C+}A_{C+}$ = 'Default with salience and attention hyperconnectivity'; A_{C-} = 'Attention hypoconnectivity'; $NS_{A+}P_{A+}$ = 'Sad-elicited negative affect with positive affect hyperactivation'; C_{A+} = 'Cognitive control hyperactivation'; $NTC_{C-}C_{A-}$ = 'Cognitive control hypoactivation with conscious threat-elicited negative affect hypoconnectivity'; $D_XS_XA_XN_XP_XC_X$ = 'Intact activation and connectivity'. *Abbreviations*: CI = confidence interval, RT = reaction time.

Biotype	Measure	Direction	N	Median	p	Effect size r	CI	Split-half	Leave-study-out
$D_{C+}S_{C+}A_{C+}$	Maze completion time	↑	133	-0.062	0.044	0.175	[-0.182; 0.166]	No	No
	Go-Nogo commission errors	↓	132	-0.312	0.002	-0.275	[-0.505; -0.217]	No	Yes
	Working memory omission errors	↑	134	-0.326	0.051	0.168	[-0.331; 0.129]	No	Yes
	Working memory RT	↑	133	0.968	0.0001	0.336	[0.714; 1.099]	No	No
	Explicit sad RT	↑	134	0.015	0.001	0.289	[-0.072; 0.289]	No	Yes
A_{C-}	Go-Nogo mean RT	↓	139	0.452	6.20e-06	-0.383	[0.180; 0.510]	Yes	No
	Working memory omission errors	↑	138	-0.303	0.020	0.198	[-0.308; -0.010]	No	No
	Working memory commission errors	↑	138	-0.073	0.0004	0.300	[-0.302; -0.019]	No	No
	Implicit threat priming RT	↓	142	-0.036	0.002	-0.256	[-0.111; 0.112]	No	No
C_{A+}	Maze completion time	↑	212	-0.103	0.027	0.152	[-0.164; 0.090]	No	No
	Maze errors	↑	212	-0.240	0.017	0.164	[-0.268; -0.027]	No	No
	Go-Nogo commission errors	↑	212	-0.034	0.022	0.158	[-0.201; 0.035]	No	Yes
	Working memory omission errors	↑	212	-0.333	6.46e-05	0.275	[-0.045; 0.170]	Yes	Yes
$NTC_{C-}C_{A-}$	Explicit sad RT	↑	11	-0.194	0.831	0.081	[-1.016; 0.915]	No	No
$D_XS_XA_XN_XP_XC_X$	Implicit threat priming RT	↑	38	0.353	0.001	0.516	[0.254; 0.611]	No	No

Supplementary Table 7: Between biotype comparisons of behavioral performance.

For each behavioral measure, the scores of participants in each biotype were compared to the median of participants not in the biotype using a Mann-Whitney U test. Here, we show the direction of the difference (↑ =symptom median was higher in the biotype, ↓ =symptom median was lower in the biotype), number of participants used for this comparison in each biotype, the median, the two-sided p-value of the test, a measure of effect size r, calculated as the Z statistic divided by square root of the sample size and confidence interval (CI). We named each biotype according to the circuits and circuit features that specifically differentiated each relative to the healthy reference. We used the following nomenclature: each circuit is indicated with a letter (D = default mode, S = salience, A = attention, NS = negative affect circuit evoked by sad stimuli, NTC = negative affect circuit evoked by conscious threat stimuli, NTN = negative affect circuit evoked by non-conscious threat stimuli, P = positive circuit, C = cognitive circuit), the distinguishing circuit feature is indicated as a subscript (C = connectivity or A = activity) and the direction of dysfunction is indicated by + or -. The subscript x indicates that the sixth biotype is not differentiated by a prominent circuit dysfunction relative to other biotypes. Besides this nomenclature, we suggest a short plain-English description for each biotype (in quotes), that connects them with our theoretically synthesized biotypes: $D_{C+}S_{C+}A_{C+}$ = 'Default with salience and attention hyperconnectivity'; A_{C-} = 'Attention hypoconnectivity'; $NS_{A+}P_{A+}$ = 'Sad-elicited negative affect with positive affect hyperactivation'; C_{A+} = 'Cognitive control hyperactivation'; $NTC_{C-}C_{A-}$ = 'Cognitive control hypoactivation with conscious threat-elicited negative affect hypoconnectivity'; $D_XS_XA_XN_XP_XC_X$ = 'Intact activation and connectivity'. *Abbreviations: CI = confidence interval, RT = reaction time.*

Biotype	Symptom	Direction	N	Median	p	Effect size r	CI
$D_{C+}S_{C+}A_{C+}$	Maze completion time	↑	133	-0.062	0.044	0.175	[-0.182; 0.166]
	Maze errors	↑	133	-0.239	0.089	0.147	[-0.308; 0.006]
	Go-Nogo mean RT	↓	131	0.753	0.730	-0.030	[0.523; 0.785]
	Go-Nogo commission errors	↓	132	-0.312	0.002	-0.275	[-0.505; -0.217]
	Working memory omission errors	↑	134	-0.326	0.051	0.168	[-0.331; 0.129]
	Working memory commission errors	↑	133	-0.363	0.467	0.063	[-0.355; -0.121]
	Working memory RT	↑	133	0.968	0.0001	0.336	[0.714; 1.099]
	Implicit threat priming RT	↓	134	0.103	0.213	-0.108	[-0.093; 0.182]
	Implicit happy RT	↑	134	-0.554	0.263	0.097	[-0.702; -0.345]
	Implicit sad RT	↑	134	-0.264	0.405	0.072	[-0.370; -0.018]
	Explicit threat RT	↑	134	-0.118	0.137	0.129	[-0.207; 0.158]
	Explicit happy RT	↑	134	-0.015	0.079	0.152	[-0.105; 0.263]
	Explicit sad RT	↑	134	0.015	0.001	0.289	[-0.072; 0.289]
A_{C-}	Maze completion time	↑	142	-0.290	0.567	0.048	[-0.263; 0.075]
	Maze errors	↓	142	-0.316	0.943	-0.006	[-0.409; -0.125]
	Go-Nogo mean RT	↓	139	0.452	6.20e-06	-0.383	[0.180; 0.510]
	Go-Nogo commission errors	↑	139	-0.191	0.808	0.021	[-0.279; -0.012]
	Working memory omission errors	↑	138	-0.303	0.020	0.198	[-0.308; -0.010]
	Working memory commission errors	↑	138	-0.073	0.0004	0.300	[-0.302; -0.019]
	Working memory RT	↑	138	0.683	0.437	0.066	[0.453; 0.840]
	Implicit threat priming RT	↓	142	-0.036	0.002	-0.256	[-0.111; 0.112]
	Implicit happy RT	↑	142	-0.604	0.648	0.038	[-0.771; -0.400]
	Implicit sad RT	↑	142	-0.165	0.144	0.123	[-0.343; 0.020]
	Explicit threat RT	↑	142	-0.189	0.645	0.039	[-0.270; 0.111]
	Explicit happy RT	↑	142	-0.101	0.837	0.017	[-0.212; 0.140]
	Explicit sad RT	↓	142	-0.232	0.431	-0.066	[-0.342; -0.007]
$NS_{A+}P_{A+}$	Maze completion time	↓	131	-0.184	0.994	-0.001	[-0.320; 0.032]
	Maze errors	↑	131	-0.305	0.438	0.068	[-0.365; -0.047]
	Go-Nogo mean RT	↓	131	0.742	0.851	-0.016	[0.566; 0.795]
	Go-Nogo commission errors	↓	131	-0.210	0.527	-0.055	[-0.398; -0.063]
	Working memory omission errors	↑	131	-0.347	0.197	0.113	[-0.409; 0.159]
	Working memory commission errors	↓	131	-0.529	0.871	-0.014	[-0.409; -0.143]

	Working memory RT	↓	131	0.522	0.317	-0.087	[0.359; 0.721]
	Implicit threat priming RT	↑	131	0.205	0.261	0.099	[0.074; 0.321]
	Implicit happy RT	↑	131	-0.602	0.381	0.077	[-0.710; -0.403]
	Implicit sad RT	↑	131	-0.237	0.277	0.095	[-0.362; 0.017]
	Explicit threat RT	↑	131	-0.160	0.276	0.095	[-0.220; 0.128]
	Explicit happy RT	↑	131	0.058	0.155	0.124	[-0.168; 0.220]
	Explicit sad RT	↑	131	-0.090	0.100	0.143	[-0.156; 0.220]
C_{A+}	Maze completion time	↑	212	-0.103	0.027	0.152	[-0.164; 0.090]
	Maze errors	↑	212	-0.240	0.017	0.164	[-0.268; -0.027]
	Go-Nogo mean RT	↓	211	0.720	0.849	-0.013	[0.575; 0.765]
	Go-Nogo commission errors	↑	212	-0.034	0.022	0.158	[-0.201; 0.035]
	Working memory omission errors	↑	212	-0.333	6.46e-05	0.275	[-0.045; 0.170]
	Working memory commission errors	↓	212	-0.498	0.553	-0.041	[-0.401; -0.178]
	Working memory RT	↓	212	0.521	0.340	-0.066	[0.425; 0.705]
	Implicit threat priming RT	↑	212	0.165	0.295	0.072	[0.067; 0.239]
	Implicit happy RT	↑	213	-0.650	0.514	0.045	[-0.702; -0.427]
	Implicit sad RT	↑	213	-0.327	0.864	0.012	[-0.400; -0.096]
	Explicit threat RT	↑	213	-0.020	0.138	0.101	[-0.204; 0.100]
	Explicit happy RT	↑	213	-0.085	0.439	0.053	[-0.156; 0.150]
	Explicit sad RT	↓	213	-0.215	0.957	-0.004	[-0.250; 0.066]
$NTC_{C-}C_{A-}$	Maze completion time	↑	11	0.447	0.966	0.027	[-0.812; 0.630]
	Maze errors	↑	11	-0.530	0.898	0.054	[-0.740; 0.335]
	Go-Nogo mean RT	↑	11	1.040	0.520	0.214	[0.112; 1.285]
	Go-Nogo commission errors	↓	11	-0.995	0.083	-0.537	[-1.690; 0.001]
	Working memory omission errors	↑	11	-0.390	0.577	0.188	[-0.623; 0.221]
	Working memory commission errors	↑	11	-0.075	0.197	0.401	[-0.490; 1.126]
	Working memory RT	↓	11	0.220	0.365	-0.295	[-0.531; 1.219]
	Implicit threat priming RT	↑	11	0.140	0.577	0.188	[0.025; 0.405]
	Implicit happy RT	↓	11	-0.550	0.577	-0.188	[-1.256; -0.234]
	Implicit sad RT	↓	11	-0.830	0.024	-0.669	[-1.316; -0.315]
	Explicit threat RT	↓	11	-0.345	0.465	-0.241	[-1.050; 0.332]
	Explicit happy RT	↓	11	-0.248	0.898	-0.054	[-0.840; 0.786]
	Explicit sad RT	↑	11	-0.194	0.831	0.081	[-1.016; 0.915]
$D_XS_XA_XN_XP_XC_X$	Maze completion time	↓	37	-0.385	0.059	-0.311	[-0.738; -0.144]
	Maze errors	↑	37	-0.340	0.916	0.019	[-0.465; -0.028]
	Go-Nogo mean RT	↑	38	0.787	0.098	0.269	[0.640; 1.020]
	Go-Nogo commission errors	↓	38	-0.276	0.353	-0.152	[-0.689; 0.046]
	Working memory omission errors	↑	38	-0.319	0.090	0.276	[-0.365; 0.395]
	Working memory commission errors	↑	38	-0.151	0.612	0.084	[-0.445; -0.040]
	Working memory RT	↓	38	0.549	0.546	-0.099	[0.095; 0.895]
	Implicit threat priming RT	↑	38	0.353	0.001	0.516	[0.254; 0.611]
	Implicit happy RT	↓	38	-0.714	0.172	-0.222	[-1.010; -0.530]
	Implicit sad RT	↑	38	-0.362	0.662	0.072	[-0.569; 0.255]
	Explicit threat RT	↓	38	-0.288	0.785	-0.046	[-0.505; 0.168]
	Explicit happy RT	↓	38	-0.409	0.207	-0.206	[-0.509; 0.035]
	Explicit sad RT	↓	38	-0.245	0.922	-0.017	[-0.386; 0.205]

Supplementary Table 8: Biotype distributions for number of participants receiving each treatment.

We report how many participants of each biotype received each treatment in the randomized clinical trial dataset. For comparisons of post-treatment severity, we chose to exclude combinations of treatment and biotype that had ≤5 participants. We named each biotype according to the circuits and circuit features that specifically differentiated each relative to other biotypes and to the healthy reference. We used the following nomenclature: each circuit is indicated with a letter (D = default mode, S = salience, A = attention, NS = negative affect circuit evoked by sad stimuli, NTC = negative affect circuit evoked by conscious threat stimuli, NTN = negative affect circuit evoked by non-conscious threat stimuli, P = positive circuit, C = cognitive circuit), the distinguishing circuit feature is indicated as a subscript (C = connectivity or A = activity) and the direction of dysfunction is indicated by + or -. The subscript x indicates that the sixth biotype is not differentiated by a prominent circuit dysfunction relative to other biotypes. Besides this nomenclature, we suggest a short plain-English description for each biotype (in quotes), that connects them with our theoretically synthesized biotypes: $D_{C+}S_{C+}A_{C+}$ = 'Default with salience and attention hyperconnectivity'; A_{C-} = 'Attention hypoconnectivity'; $NS_{A+}P_{A+}$ = 'Sad-elicited negative affect with positive affect hyperactivation'; C_{A+} = 'Cognitive control hyperactivation'; $NTC_{C-}C_{A-}$ = 'Cognitive control hypoactivation with conscious threat-elicited negative affect hypoconnectivity'; $D_XS_XA_XN_XP_XC_X$ = 'Intact activation and connectivity'. [1]·Biotype-treatment combinations for which the comparison of post-treatment severity with other biotypes was not conducted because of too small sample size (N≤5).

Biotype	Escitalopram	Sertraline	Venlafaxine	I-CARE	U-CARE
$D_{C+}S_{C+}A_{C+}$	16	13	9	12	14
A_{C-}	11	7	3[1]	27	20
$NS_{A+}P_{A+}$	13	10	8	1[1]	1[1]
C_{A+}	18	18	25	0[1]	0[1]
$NTC_{C-}C_{A-}$	0[1]	2[1]	2[1]	0[1]	0[1]
$D_XS_XA_XN_XP_XC_X$	16	13	9	12	14

Supplementary Table 9: Between biotype comparisons of treatment outcomes.

The severity after treatment of participants in each biotype was compared to the median of participants not in the biotype using a Mann-Whitney U test for each treatment separately. Here, we show the direction of the difference (↑ =symptom median was higher in the biotype, ↓ =symptom median was lower in the biotype), number of participants used for this comparison in each biotype, the median, the two-sided p-value of the test, a measure of effect size r, calculated as the Z statistic divided by square root of the sample size and confidence interval (CI). Comparisons with N<6 were discarded. Significant comparisons (p<0.05) are bolded. We named each biotype according to the circuits and circuit features that specifically differentiated each relative to other biotypes and to the healthy reference. We used the following nomenclature: each circuit is indicated with a letter (D = default mode, S = salience, A = attention, NS = negative affect circuit evoked by sad stimuli, NTC = negative affect circuit evoked by conscious threat stimuli, NTN = negative affect circuit evoked by non-conscious threat stimuli, P = positive circuit, C = cognitive circuit), the distinguishing circuit feature is indicated as a subscript (C = connectivity or A = activity) and the direction of dysfunction is indicated by + or -. The subscript x indicates that the sixth biotype is not differentiated by a prominent circuit dysfunction relative to other biotypes. Besides this nomenclature, we suggest a short plain-English description for each biotype (in quotes), that connects them with our theoretically synthesized biotypes: $D_{C+}S_{C+}A_{C+}$ = ‘Default with salience and attention hyperconnectivity’; A_{C-} = ‘Attention hypoconnectivity’; $NS_{A+}P_{A+}$ = ‘Sad-elicited negative affect with positive affect hyperactivation’; C_{A+} = ‘Cognitive control hyperactivation’; $NTC_{C-}C_{A-}$ = ‘Cognitive control hypoactivation with conscious threat-elicited negative affect hypoconnectivity’; $D_XS_XA_XN_XP_XC_X$ = ‘Intact activation and connectivity’. *Abbreviations*: CI = confidence interval, I-CARE=active behavioral therapy, U-CARE=usual care.

Biotype	Treatment	Direction	N	Median	p	Effect size r	CI
$D_{C+}S_{C+}A_{C+}$	Escitalopram	↑	16	0.198	0.155	0.363	[0.151; 0.245]
	**I-CARE**	↓	**12**	**0.175**	**0.037**	**0.612**	**[0.137; 0.306]**
	Sertraline	↑	13	0.226	0.080	0.494	[0.160; 0.264]
	U-CARE	↓	14	0.275	0.802	0.076	[0.169; 0.350]
	Venlafaxine	↑	9	0.226	0.342	0.337	[0.132; 0.321]
A_{C-}	Escitalopram	↓	11	0.151	0.504	0.215	[0.113; 0.274]
	**I-CARE**	↑	**27**	**0.313**	**0.002**	**0.593**	**[0.219; 0.350]**
	Sertraline	↓	7	0.170	0.446	0.320	[0.085; 0.236]
	U-CARE	↓	20	0.250	0.287	0.241	[0.200; 0.337]
$NS_{A+}P_{A+}$	Escitalopram	↑	13	0.208	0.724	0.109	[0.123; 0.245]
	Sertraline	↓	10	0.142	0.476	0.244	[0.085; 0.255]
	Venlafaxine	↓	8	0.170	0.325	0.371	[0.104; 0.274]
C_{A+}	Escitalopram	↓	18	0.170	1.000	0.006	[0.132; 0.255]
	Sertraline	↓	18	0.179	0.962	0.017	[0.132; 0.264]
	**Venlafaxine**	↓	**25**	**0.170**	**0.034**	**0.426**	**[0.132; 0.226]**

Supplementary Table 10: Biotype distribution of treatment response and remission rates.
Response was defined as a 50% reduction in symptoms at follow-up, and remission was defined as a follow-up Hamilton Depression Rating Scale score ≤7 (collected in iSPOT-D) and Symptom Checklist 20 Depression Scale score ≤0.5 (collected in ENGAGE). We named each biotype according to the circuits and circuit features that specifically differentiated each relative to other biotypes and to the healthy reference. We used the following nomenclature: each circuit is indicated with a letter (D = default mode, S = salience, A = attention, NS = negative affect circuit evoked by sad stimuli, NTC = negative affect circuit evoked by conscious threat stimuli, NTN = negative affect circuit evoked by non-conscious threat stimuli, P = positive circuit, C = cognitive circuit), the distinguishing circuit feature is indicated as a subscript (C = connectivity or A = activity) and the direction of dysfunction is indicated by + or -. The subscript x indicates that the sixth biotype is not differentiated by a prominent circuit dysfunction relative to other biotypes. Besides this nomenclature, we suggest a short plain-English description for each biotype (in quotes), that connects them with our theoretically synthesized biotypes: $D_{C+}S_{C+}A_{C+}$ = 'Default with salience and attention hyperconnectivity'; A_{C-} = 'Attention hypoconnectivity'; $NS_{A+}P_{A+}$ = 'Sad-elicited negative affect with positive affect hyperactivation'; C_{A+} = 'Cognitive control hyperactivation'; $NTC_{C-}C_{A-}$ = 'Cognitive control hypoactivation with conscious threat-elicited negative affect hypoconnectivity'; $D_XS_XA_XN_XP_XC_X$ = 'Intact activation and connectivity'. *Abbreviations:* I-CARE=active behavioral therapy; U-CARE=usual care.

Biotype	Treatment	Responders	Remitters
$D_{C+}S_{C+}A_{C+}$	U-CARE	4 (29%)	4 (29%)
	I-CARE	5 (42%)	3 (25%)
	Escitalopram	9 (56%)	5 (31%)
	Sertraline	6 (46%)	3 (23%)
	Venlafaxine	2 (22%)	2 (22%)
A_{C-}	U-CARE	8 (40%)	1 (5%)
	I-CARE	7 (26%)	6 (22%)
	Escitalopram	7 (64%)	4 (36%)
	Sertraline	4 (57%)	3 (43%)
	Venlafaxine	2 (67%)	2 (67%)
$NS_{A+}P_{A+}$	U-CARE	0 (0%)	0 (0%)
	I-CARE	0 (0%)	0 (0%)
	Escitalopram	6 (46%)	4 (31%)
	Sertraline	6 (60%)	5 (50%)
	Venlafaxine	4 (50%)	4 (50%)
C_{A+}	U-CARE	-	-
	I-CARE	-	-
	Escitalopram	11 (61%)	6 (33%)
	Sertraline	10 (56%)	7 (39%)
	Venlafaxine	16 (64%)	10 (40%)
$NTC_{C-}C_{A-}$	U-CARE	-	-
	I-CARE	-	-
	Escitalopram	-	-
	Sertraline	2 (100%)	1 (50%)
	Venlafaxine	0 (0%)	0 (0%)
$D_XS_XA_XN_XP_XC_X$	U-CARE	-	-
	I-CARE	-	-
	Escitalopram	1 (100%)	1 (100%)
	Sertraline	1 (50%)	1 (100%)
	Venlafaxine	1 (33%)	1 (100%)

Supplementary Table 11: Biotype distribution of number of participants by dataset.

We show how many participants belonged to each biotype in each dataset. Biotypes were represented differently between datasets (chi-square = 161.37, two-sided p=2.2e-16). We named each biotype according to the circuits and circuit features that specifically differentiated each relative to other biotypes and to the healthy reference. We used the following nomenclature: each circuit is indicated with a letter (D = default mode, S = salience, A = attention, NS = negative affect circuit evoked by sad stimuli, NTC = negative affect circuit evoked by conscious threat stimuli, NTN = negative affect circuit evoked by non-conscious threat stimuli, P = positive circuit, C = cognitive circuit), the distinguishing circuit feature is indicated as a subscript (C = connectivity or A = activity) and the direction of dysfunction is indicated by + or -. The subscript x indicates that the sixth biotype is not differentiated by a prominent circuit dysfunction relative to other biotypes. Besides this nomenclature, we suggest a short plain-English description for each biotype (in quotes), that connects them with our theoretically synthesized biotypes: $D_{C+}S_{C+}A_{C+}$ = 'Default with salience and attention hyperconnectivity'; A_{C-} = 'Attention hypoconnectivity'; $NS_{A+}P_{A+}$ = 'Sad-elicited negative affect with positive affect hyperactivation'; C_{A+} = 'Cognitive control hyperactivation'; $NTC_{C-}C_{A-}$ = 'Cognitive control hypoactivation with conscious threat-elicited negative affect hypoconnectivity'; $D_XS_XA_XN_XP_XC_X$ = 'Intact activation and connectivity'. *Abbreviations:* ENGAGE=Engaging self-regulation targets to understand the mechanisms of behavior change and improve mood and weight outcome; HCP-DES=Human Connectome Project for Disordered Emotional States; iSPOT-D=International Study to Predict Optimized Treatment in Depression; RAD=Research on Anxiety and Depression study.

Biotype	ENGAGE	HCP-DES	iSPOT-D	RAD
$D_{C+}S_{C+}A_{C+}$	29 (17%)	40 (24%)	45 (27%)	55 (33%)
A_{C-}	55 (34%)	33 (20%)	26 (16%)	47 (29%)
$NS_{A+}P_{A+}$	2 (1%)	51 (33%)	38 (25%)	63 (41%)
C_{A+}	0 (0%)	68 (26%)	76 (29%)	114 (44%)
$NTC_{C-}C_{A-}$	0 (0%)	6 (40%)	4 (27%)	5 (33%)
$D_XS_XA_XN_XP_XC_X$	0 (0%)	10 (23%)	9 (20%)	25 (57%)

Supplementary Table 12: Biotype overlap with diagnoses.

Number and proportion of participants in each biotype that meet diagnostic criteria for major depressive disorder, generalized anxiety disorder, panic disorder, social anxiety disorder and obsessive-compulsive disorder. We named each biotype according to the circuits and circuit features that specifically differentiated each relative to other biotypes and to the healthy reference. We used the following nomenclature: each circuit is indicated with a letter (D = default mode, S = salience, A = attention, NS = negative affect circuit evoked by sad stimuli, NTC = negative affect circuit evoked by conscious threat stimuli, NTN = negative affect circuit evoked by non-conscious threat stimuli, P = positive circuit, C = cognitive circuit), the distinguishing circuit feature is indicated as a subscript (C = connectivity or A = activity) and the direction of dysfunction is indicated by + or -. The subscript x indicates that the sixth biotype is not differentiated by a prominent circuit dysfunction relative to other biotypes. Besides this nomenclature, we suggest a short plain-English description for each biotype (in quotes), that connects them with our theoretically synthesized biotypes: $D_{C+}S_{C+}A_{C+}$ = 'Default with salience and attention hyperconnectivity'; A_{C-} = 'Attention hypoconnectivity'; $NS_{A+}P_{A+}$ = 'Sad-elicited negative affect with positive affect hyperactivation'; C_{A+} = 'Cognitive control hyperactivation'; $NTC_{C-}C_{A-}$ = 'Cognitive control hypoactivation with conscious threat-elicited negative affect hypoconnectivity'; $D_XS_XA_XN_XP_XC_X$ = 'Intact activation and connectivity'.

Biotype	Major depressive disorder	Generalized anxiety disorder	Panic disorder	Social anxiety disorder	Obsessive-compulsive disorder
**$D_{C+}S_{C+}A_{C+}$**					
Does not meet criteria	72 (44%)	98 (72%)	155 (95%)	101 (74%)	126 (93%)
Meets criteria	93 (56%)	38 (28%)	9 (9%)	35 (26%)	10 (7%)
**A_{C-}**					
Does not meet criteria	65 (41%)	78 (76%)	140 (89%)	78 (76%)	97 (94%)
Meets criteria	93 (59%)	25 (24%)	17 (11%)	25 (24%)	6 (6%)
**$NS_{A+}P_{A+}$**					
Does not meet criteria	85 (57%)	105 (72%)	135 (92%)	107 (73%)	131 (90%)
Meets criteria	63 (43%)	41 (28%)	12 (8%)	39 (27%)	15 (10%)
**C_{A+}**					
Does not meet criteria	143 (57%)	183 (73%)	221 (88%)	187 (74%)	239 (95%)
Meets criteria	109 (43%)	69 (27%)	31 (12%)	65 (26%)	13 (5%)
**$NTC_{C-}C_{A-}$**					
Does not meet criteria	10 (71%)	9 (64%)	12 (86%)	7 (50%)	14 (100%)
Meets criteria	4 (29%)	5 (36%)	2 (14%)	7 (50%)	0 (0%)
**$D_XS_XA_XN_XP_XC_X$**					
Does not meet criteria	31 (70%)	30 (68%)	40 (91%)	36 (82%)	41 (93%)
Meets criteria	13 (30%)	14 (32%)	4 (9%)	8 (18%)	3 (7%)

Supplementary Table 13: Comparison of the performance of our brain circuit features to other features.
We selected three competing alternative feature sets, each used in a recent paper reporting the identification of biotypes of depression using resting state fMRI [12–14]. We did not replicate the entire analysis workflow used in these studies. Rather, we used them to derive alternative imaging feature sets supported by previous evidence that we then entered in our own analysis pipeline, validating the results with the same criteria and procedures we used for our own features. For each of these alternative sets, we tested the number of clusters reported in the original paper. We named each biotype according to the circuits and circuit features that specifically differentiated each relative to other biotypes and to the healthy reference. We used the following nomenclature: each circuit is indicated with a letter (D = default mode, S = salience, A = attention, NS = negative affect circuit evoked by sad stimuli, NTC = negative affect circuit evoked by conscious threat stimuli, NTN = negative affect circuit evoked by non-conscious threat stimuli, P = positive circuit, C = cognitive circuit), the distinguishing circuit feature is indicated as a subscript (C = connectivity or A = activity) and the direction of dysfunction is indicated by + or -. The subscript x indicates that the sixth biotype is not differentiated by a prominent circuit dysfunction relative to other biotypes. Besides this nomenclature, we suggest a short plain-English description for each biotype (in quotes), that connects them with our theoretically synthesized biotypes: $D_{C+}S_{C+}A_{C+}$ = 'Default with salience and attention hyperconnectivity'; A_{C-} = 'Attention hypoconnectivity'; $NS_{A+}P_{A+}$ = 'Sad-elicited negative affect with positive affect hyperactivation'; C_{A+} = 'Cognitive control hyperactivation'; $NTC_{C-}C_{A-}$ = 'Cognitive control hypoactivation with conscious threat-elicited negative affect hypoconnectivity'; $D_XS_XA_XN_XP_XC_X$ = 'Intact activation and connectivity'. We highlight in red tests for which the alternative biotyping strategy did not outperform the strategy presented in the current work and in green tests for which the alternative biotyping strategy outperformed the strategy presented in the current work. [1.] Excluding features with bad coverage for >5% of participants. [2.] Too many features to compute a covariance matrix for the simulation. Abbreviations: AAL = automatic atlas labeling, Clu = cluster, Δ = difference, sil = silhouette index.

**Biotyping strategy**	**Regional brain circuit scores 6 clusters**	**Whole-brain connectome 2 clusters**	**Default mode network 2 clusters**	**Angular gyrus network 3 clusters**
Example of study using the imaging features	Current work	Drysdale et al. 2017	Liang et al. 2020	Tokuda et al. 2018
Feature extraction	Task activation and rest and task connectivity from a-priori regions of interest	Power atlas rest connectome[1]	Power atlas rest connectome default mode network connections[1]	AAL rest connectome connections from Tokuda et al. Fig. 5
Number of features for clustering	41	16	916	12
Silhouette difference compared to current work (resampling test)	-	Δsil=-0.026, p=0.049	Δsil=-0.012, p=0.256	Δsil=0.155, p=1
Silhouette difference compared to current work (permutation test)	-	Δsil=-0.026, p=0	Δsil=-0.012, p=0	Δsil=0.155, p=1
Evaluation criteria:				
1. Solution outperforms null hypothesis of no clusters (simulated data)	sil=0.065, p=0.016	- [2]	sil=0.053, p=1	sil=0.220, p=0.777
2. Solution outperforms null hypothesis of no clusters (permuted data)	sil=0.065, p=0	sil=0.045, p=0	sil=0.053, p=0	sil=0.220, p=0
3. Adjusted Rand Index (leave one out mean)	0.80	0.94	0.77	0.81
4. Adjusted Rand Index (leave 20% out mean)	0.35	0.48	0.38	0.48
5. Generalizable cluster profiles across random split-half	$D_{C+}S_{C+}A_{C}$, A_{C-}, $NS_{A+}P_{A+}$, C_{A+}, $NTC_{C-}C_{A}$, $D_XS_XA_XN_XP_XC_X$	Clu 1, Clu 2, Clu 3, Clu 4	Clu 1, Clu 2	Clu 1, Clu 2, Clu 3
6. Generalizable symptom differences across random split-half	C_{A+} threat dysregulation ↑, C_{A+} negative bias ↑	Clu 1 negative bias ↑	None	None

7. Generalizable behavior differences across random split-half	A_{C-} Go-NoGo RT ↓<br>C_{A+} working memory omission errors ↑	Clu 1 Go-Nogo RT ↓<br>Clu 1 Working memory commission errors ↑<br>Clu 2 Explicit sad RT ↑<br>Clu 4 Go-Nogo RT ↑<br>Clu 4 Working memory omission errors ↑<br>Clu 4 Working memory RT ↑	Clu 2 Working memory omission errors ↑	None
8. Generalizable symptom differences across leave-study-out	C_{A+} threat dysregulation ↑	Clu 1 tension ↑<br>Clu 2 anxious arousal ↑<br>Clu 3 anxious arousal ↑	Clu 1 negative bias ↑<br>Clu 2 anxious arousal ↑	Clu 1 tension ↑
9. Generalizable behavior differences across leave-study-out	$D_{C+}S_{C+}A_{C+}$ Go-Nogo commission errors ↓<br>$D_{C+}S_{C+}A_{C+}$ Working memory omission errors ↑<br>$D_{C+}S_{C+}A_{C+}$ Explicit sad RT ↑<br>C_{A+} Go-Nogo commission errors ↑<br>C_{A+} Working memory omission errors ↑	Clu 1 Working memory commission errors ↑<br>Clu 4 Go-Nogo RT ↑<br>Clu 4 Working memory omission errors ↑	Clu 1 Explicit happy RT ↑<br>Clu 1 Maze completion time ↑<br>Clu 1 Working memory commission errors ↑<br>Clu 1 Working memory omission errors ↓<br>Clu 2 Working memory omission errors ↑	Clu 1 Working memory omission errors ↑<br>Clu 1 Explicit happy RT ↑<br>Clu 2 Working memory omission errors ↑<br>Clu 2 Maze completion time ↑<br>Clu 3 Working memory commission errors ↑
10. Biotypes differ in treatment response	$D_{C+}S_{C+}A_{C+}$ I-CARE ↓<br>A_{C-} I-CARE ↑<br>C_{A+} venlafaxine ↓	Clu 1 sertraline ↑<br>Clu 1 venlafaxine ↑<br>Clu 2 venlafaxine ↓	None	Clu 1 sertraline ↓<br>Clu 3 sertraline ↑<br>Clu 3 venlafaxine ↑

Supplementary Table 14. Comparison of our brain circuit features to other features using six clusters.

We selected three competing alternative feature sets, each used in a recent paper reporting the identification of biotypes of depression using resting state fMRI [12–14]. We did not replicate the entire analysis workflow used in these studies. Rather, we used them to derive alternative imaging feature sets supported by previous evidence that we then entered in our own analysis pipeline, validating the results with the same criteria and procedures we used for our own features. For each of these alternative sets, we tested the number of clusters we chose in our own analysis. We highlight in red tests for which the alternative biotyping strategy did not outperform the strategy presented in the current work and in green tests for which the alternative biotyping strategy outperformed the strategy presented in the current work. For the resampling test of the silhouette index, the p-value is defined as the fraction of mean silhouettes greater than our result obtained by clustering 10,000 synthetic datasets from a multivariate normal distribution. For the permutation test of the silhouette index, we shuffled each brain circuit score across subjects 10,000 times, then repeated the hierarchical clustering procedure and calculated the average silhouette index. Thus, we obtained null distributions for these average silhouette indexes, comprising 10,000 observations. We computed a p-value defined as the fraction of average silhouette indexes in this null distribution greater than our result. [1.]Excluding features with bad coverage for >5% of participants; [2.] Too many features to compute a covariance matrix for the simulation; [3] Too many features to visually assess if cluster profiles were comparable. *Abbreviations:* AAL = automatic atlas labeling, Clu. = cluster, sil = silhouette index.

**Biotyping strategy**	**Regional brain circuit scores 6 clusters**	**Whole-brain connectome 6 clusters**	**Default mode network 6 clusters**	**Angular gyrus network 6 clusters**
Example of study using the imaging features	Current work	Drysdale et al. 2017	Liang et al. 2020	Tokuda et al. 2018
Feature extraction	Task activation and rest and task connectivity from a-priori regions of interest	Power atlas rest connectome[1]	Power atlas rest connectome default mode network connections[1]	AAL rest connectome connections from Tokuda et al. Fig. 5
Number of features for clustering	41	29,161	916	12
Silhouette difference compared to current work (resampling test)	-	Δsil=-0.039, p=0.021	Δsil=-0.032, p=0.037	Δsil=0.139, p=1
Silhouette difference compared to current work (permutation test)	-	Δsil=-0.039, p=0.001	Δsil=-0.032, p=0	Δsil=0.139, p=1
Evaluation criteria:				
1. Solution outperforms null hypothesis of no clusters (simulated data)	sil=0.06, p=0.02	-[2]	sil=0.033, p=0.321	sil=0.204, p=0.079
2. Solution outperforms null hypothesis of no clusters (permuted data)	sil=0.06, p=0	sil.=0.031, p=0	sil=0.033, p=0	sil=0.204, p=0
3. Adjusted Rand Index (leave one out mean)	0.80	0.93	0.80	0.85
4. Adjusted Rand Index (leave 20% out mean)	0.35	0.47	0.35	0.47
5. Generalizable cluster profiles across random split-half	$D_{C+}S_{C+}A_{C}$, A_{C-}, $NS_{A+}P_{A+}$, C_{A+}, NTC_{C}-C_{A}, $D_{X}S_{X}A_{X}N_{X}P_{X}C_{X}$	Clu 1, Clu 2, Clu 3, Clu 4, Clu 5, Clu 6	Clu 1, Clu 2, Clu 3, Clu 4, Clu 5, Clu 6	Clu 1, Clu 2, Clu 3, Clu 4, Clu 5, Clu 6
6. Generalizable symptom differences across random split-half	C_{A+} threat dysregulation ↑, C_{A+} negative bias ↑	Clu 1 negative bias ↑	None	None
7. Generalizable behavior differences across random split-half	A_{C-} Go-NoGo RT ↓ C_{A+} working memory omission errors ↑	Clu 1 working memory commission errors ↑ Clu 4 working memory omission errors ↑	Clu 3 working memory commission errors ↑ Clu 3 working memory omission errors ↑ Clu 3 Go-NoGo RT ↑	Clu 3 Explicit threat RT ↑

			Clu 3 Maze completion time ↑<br>Clu 3 Maze errors ↑	
8. Generalizable symptom differences across leave-study-out	C_{A+} threat dysregulation ↑	Clu 2 anxious arousal ↑	None	None
9. Generalizable behavior differences across leave-study-out	$D_{C+}S_{C+}A_{C+}$ Go-Nogo commission errors ↓<br>$D_{C+}S_{C+}A_{C+}$ Working memory omission errors ↑<br>$D_{C+}S_{C+}A_{C+}$ Explicit sad RT ↑<br>C_{A+} Go-Nogo commission errors ↑<br>C_{A+} Working memory omission errors ↑	None	Clu 3 working memory commission errors ↑<br>Clu 3 working memory omission errors ↑<br>Clu 3 Maze completion time ↑<br>Clu 3 Maze errors ↑<br>Clu 2 Maze completion time ↑	Clu 1 Maze errors ↑<br>Clu 1 working memory omission errors ↑<br>Clu 3 Explicit happy RT ↑<br>Clu 4 Maze completion time ↑
10. Biotypes differ in treatment response	$D_{C+}S_{C+}A_{C+}$ I-CARE ↓<br>A_{C-} I-CARE ↑<br>C_{A+} venlafaxine ↓	Clu 1 sertraline ↑<br>Clu 1 venlafaxine ↑<br>Clu 2 venlafaxine ↓	None	Clu 4 venlafaxine ↑

Supplementary Table 15. Comparison of our brain circuit features to resting state features only.

To assess the impact of including task fMRI measures in addition to task-free measures only, we compared our original results to results obtained using only our task-free brain circuit scores. We validated the results with the same criteria and procedures we used for our own features and chose as number of clusters 6 (the number we chose in our analysis using all features) or 2 (the number of clusters with task-free dysfunction identified in our analyses). We highlight in red tests for which the alternative biotyping strategy did not outperform the strategy presented in the current work and in green tests for which the alternative biotyping strategy outperformed the strategy presented in the current work. For the resampling test of the silhouette index, the p-value is defined as the fraction of mean silhouettes greater than our result obtained by clustering 10,000 synthetic datasets from a multivariate normal distribution. For the permutation test of the silhouette index, we shuffled each brain circuit score across subjects 10,000 times, then repeated the hierarchical clustering procedure and calculated the average silhouette index. Thus, we obtained null distributions for these average silhouette indexes, comprising 10,000 observations. We computed a p-value defined as the fraction of average silhouette indexes in this null distribution greater than our result. *Abbreviations:* Clu = cluster, sil = silhouette index.

**Biotyping strategy**	**Regional brain circuit scores 6 clusters**	**Regional brain circuit scores (rest only) 2 clusters**	**Regional brain circuit scores (rest only) 6 clusters**
Feature extraction	Task activation and rest and task connectivity from a-priori regions of interest	Rest connectivity from a-priori regions of interest	Rest connectivity from a-priori regions of interest
Number of features for clustering	41	14	14
Evaluation criteria			
1. Solution outperforms null hypothesis of no clusters (simulated data)	sil=0.065, p=0.016	sil.=0.189, p=0.485	sil.=0.092, p=0.283
2. Solution outperforms null hypothesis of no clusters (permuted data)	sil=0.065, p=0	sil.=0.189, p=0	sil.=0.092, p=0
3. Adjusted Rand Index (leave one out mean)	0.80	0.82	0.76
4. Adjusted Rand Index (leave 20% out mean)	0.35	0.37	0.34
5. Generalizable cluster profiles across random split-half	$D_{C+}S_{C+}A_C$, A_{C-}, $NS_{A+}P_{A+}$, C_{A+}, $NTC_{C-}C_A$, $D_XS_XA_XN_XP_XC_X$	Clu 1, Clu 2	Clu 1, Clu 2, Clu 3, Clu 4, Clu 5, Clu 6
6. Generalizable symptom differences across random split-half	C_{A+} threat dysregulation ↑, C_{A+} negative bias ↑	None	None
7. Generalizable behavior differences across random split-half	A_{C-} Go-NoGo reaction time ↓<br>C_{A+} working memory omission errors ↑	Clu 1 working memory omission errors ↑	Clu 1 working memory omission errors ↑
8. Generalizable symptom differences across leave-study-out	C_{A+} threat dysregulation ↑	Clu 1 anxious arousal ↑<br>Clu 1 cognitive dyscontrol ↑<br>Clu 1 threat dysregulation ↑<br>Clu 1 tension ↑<br>Clu 2 cognitive dyscontrol ↓	Clu 1 tension ↑
9. Generalizable behavior differences across leave-study-out	$D_{C+}S_{C+}A_{C+}$ Go-Nogo commission errors ↓<br>$D_{C+}S_{C+}A_{C+}$ Working memory omission errors ↑<br>$D_{C+}S_{C+}A_{C+}$ Explicit sad RT ↑<br>C_{A+} Go-Nogo commission errors ↑<br>C_{A+} Working memory omission errors ↑	Clu 1 Go-Nogo commission errors ↓<br>Clu 1 Go-Nogo mean RT ↑<br>Clu 1 Working memory commission ↑<br>Clu 2 Go-Nogo mean RT ↓<br>Clu 2 Implicit threat RT ↓	Clu 1 Working memory commission ↑<br>Clu 3 Working memory omission errors ↑

		Clu 2 Maze errors ↑<br>Clu 2 Working memory omission errors ↑<br>Clu 2 working memory RT ↑	
10. Biotypes differ in treatment response	$D_{C+}S_{C+}A_{C+}$ I-CARE ↓<br>A_{C-} I-CARE ↑<br>C_{A+} venlafaxine ↓	Clu 2 sertraline ↑	Clu 1 venlafaxine ↓

Supplementary Table 16: Cluster-derived biotypes comparison with theoretically synthesized biotypes.

In this table, we provide an interpretive synthesis of the cluster-derived biotypes from the clinical datasets in the present study and the theoretical taxonomy that informed this analysis. Cluster-derived biotypes from the present study are ordered to match the order of biotypes in the theoretical taxonomy based on a synthesis of extant knowledge in case-control studies (Williams, 2017, 2016). We named each biotype according to the circuits and circuit features that specifically differentiated each relative to other biotypes and to the healthy reference. We used the following nomenclature: each circuit is indicated with a letter (D = default mode, S = salience, A = attention, NS = negative affect circuit evoked by sad stimuli, NTC = negative affect circuit evoked by conscious threat stimuli, NTN = negative affect circuit evoked by non-conscious threat stimuli, P = positive circuit, C = cognitive circuit), the distinguishing circuit feature is indicated as a subscript (C = connectivity or A = activity) and the direction of dysfunction is indicated by + or -. The subscript x indicates that the sixth biotype is not differentiated by a prominent circuit dysfunction relative to other biotypes. Besides this nomenclature, we suggest a short plain-English description for each biotype (in quotes), that connects them with our theoretically synthesized biotypes. *Abbreviations*: ACC=anterior cingulate cortex; AG=angular gyrus; aI=anterior insula; aIPL=anterior inferior parietal lobule; amPFC=anterior medial prefrontal cortex; dACC=dorsal anterior cingulate cortex; DLPFC=dorsolateral prefrontal cortex; DPC=dorsal parietal cortex; LPFC=lateral prefrontal cortex; MPFC=medial prefrontal cortex; msPFC=medial superior prefrontal cortex; OFC=orbitofrontal cortex; PCC=posterior cingulate cortex; SLEA=sublenticular extended amygdala; TP=temporal pole; vmPFC=ventromedial prefrontal cortex.

Cluster-derived biotypes	Theoretically synthesized biotypes (Williams, 2016)
**A1.**<br>$D_{C+}S_{C+}A_{C+}$<br>'Default with salience and attention hyperconnectivity'<br>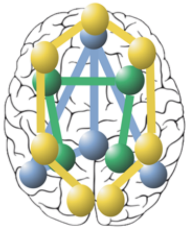<br>Distinguished by a relative intrinsic hyperconnectivity within the default mode circuit in particular and also within salience and attention circuits (Figure 3). Clinically, $D_{C+}S_{C+}A_{C+}$ was distinguished by slowed behavioral responses in identifying sad faces and target stimuli in a sustained attention task, as well as less errors in a cognitive control task, relevant to the respective involvement of both salience and attention circuits (Figure 4). $D_{C+}S_{C+}A_{C+}$ was associated with better response to behavioral treatment.	**A2.**<br>Biotypes #1and #2<br>'Default mode hyper-connectivity and altered salience and attention connectivity'<br>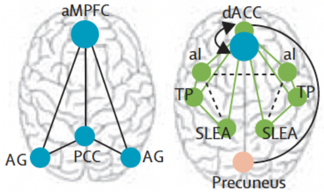<br><br>$D_{C+}S_{C+}A_{C+}$ shows equivalence to Biotype 1, combined with Biotype 2 in the theoretical taxonomy. Biotype 1 represents pronounced default mode hyperconnectivity. Biotype 2 includes a specific profile of hyperconnectivity of both the default mode and salience circuit, particularly the anterior default mode and salience circuit insula. In Biotype 2, salience and attention circuits also show hyper-connectivity, consistent with $D_{C+}S_{C+}A_{C+}$, although hypo-connectivity has also been observed and the direction may fluctuate with the nature of internal versus external events, that reflect the role of the salience circuit in guiding attention according to the salience of these events.
**B1.**<br>$NS_{A+}P_{A+}$<br>'Sad-elicited negative affect with positive affect hyperactivation'<br>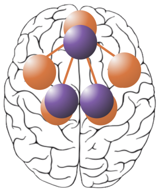<br>Distinguished by heightened activation within the negative affect circuit evoked by sad stimuli and in the positive affect circuit evoked by happy stimuli (Figures 3-4).	**B2.**<br>Biotypes #3 and #6<br>'Sad-elicited negative affect hyperactivation and positive affect frontal hyperactivation'<br>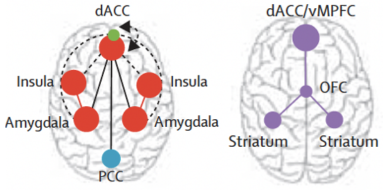<br><br>$NS_{A+}P_{A+}$ shows equivalence to Biotype 3 defined by heightened insula and amygdala activation to sad stimuli indicative of a negative bias (left) in combination with Biotype 6 defined by heightened activation of the positive affect circuit in response to primary rewards such as happy stimuli (right) in the theoretical taxonomy.

<table>
<tr>
<td>C1.
. $NTC_{C-}C_{A-}$
‘Cognitive control hypoactivation with conscious threat-elicited negative affect hypoconnectivity’:
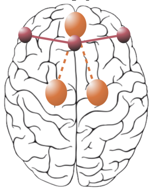
Distinguished by low functional connectivity within the negative affect circuit during nonconscious threat processing as well as reduced cognitive control circuit activity (Figures 3-4).</td>
<td>C2.
Biotypes #8 and #4
‘Cognitive control hypoactivation and conscious threat-elicited negative affect hypoconnectivity’
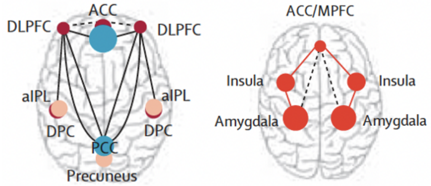
$NTC_{C-}C_{A-}$ shows equivalence to Biotype #4 combined with Biotype #8 in the theoretical taxonomy, regarding reduced amygdala-subgenual cingulate connectivity during nonconscious threat processing (left) and reduced activity within the cognitive control circuit (right), respectively.</td>
</tr>
<tr>
<td>D1.
A_{C-}
‘Attention hypoconnectivity’
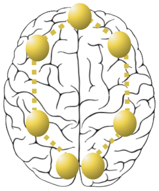
Distinguished by relatively reduced task-free intrinsic connectivity within the attention circuit (Fig. 2; Supp. Fig. 4) along with more false alarm errors on the sustained attention task and faster responses to target Go stimuli on the Go-NoGo task, which suggests reduced concentration with impulsivity (Figures 3-4).</td>
<td>D2.
Biotype #7
‘Attention hypoconnectivity’
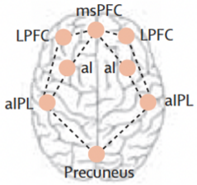
A_{C-} is equivalent to Biotype 7 in the theoretical taxonomy, which is characterized by a loss of intrinsic connectivity within the fronto-parietal attention circuit. Like A_{C-}, Biotype 7 is also characterized by a specific behavioral profile of false alarm errors consistent with disruption to sustained attention.</td>
</tr>
<tr>
<td>E1.
C_{A+}
‘Cognitive control hyperactivation’
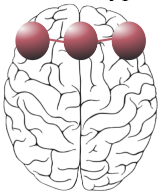
Distinguished specifically by heightened activation within the Cognitive Control circuit. Clinically, CA_{+} was characterized by more severe ruminative brooding and anhedonia and deficits in cognitive performance (Figures 3-4).</td>
<td>E2.
Compensatory Biotype #8
‘Cognitive control hyperactivation as compensation’
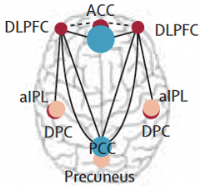
C_{A+} is equivalent to a putative compensatory form of Biotype #8 in the theoretical taxonomy. Hyperactivation (rather than hypoactivation) of the cognitive control circuit might reflect compensation associated with cognitive overdrive or a distinct cognitive dysfunction dependent on task demands.</td>
</tr>
<tr>
<td>F1.
$D_XS_XA_XN_XP_XC_X$
‘Intact activation and connectivity’
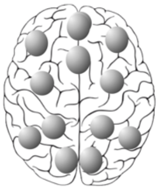
‘Intact activation and connectivity’: Although this biotype was not distinguished by marked circuit dysfunction in the present analyses, it was characterized clinically by more pronounced implicit threat priming (Figures 3-4).</td>
<td>F2.
-
‘Intact activation and connectivity’
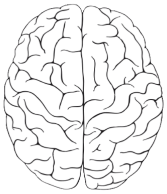
$D_XS_XA_XN_XP_XC_X$ does not have a specific equivalent biotype in the theoretical taxonomy. This is an area that warrants more study given that implicit behavioral biases to negative and mood-congruent threat and sad stimuli may characterize risk for depression and anxiety disorders and persist in the remitted state.</td>
</tr>
</table>

Supplementary Table 17: Number of scans passing quality check and motion criteria before imputation.
Number of scans available by dataset passing quality check and motion criteria before multiple imputation. We show how many participants had scans available in each of the studies. *Abbreviations*: ENGAGE=Engaging self-regulation targets to understand the mechanisms of behavior change and improve mood and weight outcome; HCP-DES=Human Connectome Project for Disordered Emotional States; iSPOT-D=International Study to Predict Optimized Treatment in Depression; RAD=Research on Anxiety and Depression study.

**Features**	**Clinical Sample 1**	**Clinical Sample 2**	**Clinical Sample 3**	**Clinical Sample 4**	**Controls Sample 1**	**Control Sample 2**
Dataset	RAD	HCP-DES	iSPOT-D	ENGAGE	HCP-DES	iSPOT-D
Task-free	288	183	188	82	62	64
Go-NoGo	296	195	195	83	65	66
Facial Expressions of Emotion conscious	281	185	182	79	64	59
Facial Expressions of Emotion nonconscious	291	190	180	80	69	65

Supplementary Table 18: Imaging features and brain regions.

We derived 41 measures of activation, task-based functional connectivity, and task-free connectivity from regions belonging to six brain circuits for which we have established relevance to depression and anxiety based on prior meta-analyses and rigorous empirical studies: a default mode circuit, a salience circuit, an attention circuit, a negative affect circuit elicited by sad and by threat, a positive affect circuit, and a cognitive control circuit. A source study established the image processing method for quantifying these circuit features and reported on the psychometric properties and reproducibility for 41 circuit features ([6]; Supplementary Tables S5A and S5B). Using this method, cortical regions of interest were defined from the meta-analytic database Neurosynth (search conducted on 06/04/2017) by identifying peak coordinates of a term search with a p_{FDR} threshold of .01 and identifying voxels at maximum 10 mm from the peak. Subcortical regions were derived from the Harvard-Oxford or AAL atlases. Regions were refined by removing those that did not pass quality control or for which circuit quantification did not meet a set of psychometric criteria, such as construct validity, internal consistency, and independence. Of the remaining regions, we only retained those which were also implicated in our theoretical synthesis of dysfunctions in depression and anxiety. From these regions we then extracted 41 features by computing intrinsic functional connectivity, task activation or task-based functional connectivity. *Abbreviations*: FDR=false discovery rate, MNI=Montreal neurological Institute, AAL=automatic labeling atlas, R=right, L=left.

**Default mode circuit**				
**Region label**	**Region anatomy**	**Atlas/MNI coordinates**	**Voxels**	**Neurosynth search**
D1	Anterior medial prefrontal cortex	-2, 50, -6	498	Term="default mode"; "resting state", Number of studies=1341;
D2	Angular gyrus L	-46, -70, 32	468	Term="default mode"; "resting state", Number of studies=1341;
D3	Angular gyrus R	50, -62, 26	462	Term="default mode"; "resting state", Number of studies=1341;
D4	Posterior cingulate	0, -50, 28	503	Term="default mode"; "resting state", Number of studies=1341;
**Computed features**	**Anatomical combinations**	**Input metrics**	**Condition**	**Task contrast**
D2D1	Anterior medial prefrontal cortex with angular gyrus L	Connectivity	Task-free	None
D1D3	Anterior medial prefrontal cortex with angular gyrus R	Connectivity	Task-free	None
D1D4	Anterior medial prefrontal cortex with posterior cingulate	Connectivity	Task-free	None
D2D4	Angular gyrus L with posterior cingulate	Connectivity	Task-free	None
D3D4	Angular gyrus R with posterior cingulate	Connectivity	Task-free	None
**Salience circuit**				
**Region label**	**Region anatomy**	**Atlas/MNI coordinates**	**Voxels**	**Neurosynth search**
S1	Anterior insula L	-38, 14, -6	345	Terms="salience network"; "salience", Number of studies=329,
S2	Anterior insula R	38, 18, 2	373	Terms="salience network"; "salience", Number of studies=329,
S3	Amygdala L	AAL	75	Terms="salience network"; "salience", Number of studies=329,
S4	Amygdala R	AAL	17	Terms="salience network"; "salience", Number of studies=329,
**Computed features**	**Anatomical combinations**	**Input metrics**	**Condition**	**Task contrast**
S1S3	Anterior insula L with amygdala L	Connectivity	Task-free	None
S2S4	Anterior insula R with amygdala R	Connectivity	Task-free	None
S1S2	Anterior insula R with anterior insula R	Connectivity	Task-free	None
**Attention circuit**				
**Region label**	**Region anatomy**	**Atlas/MNI coordinates**	**Voxels**	**Neurosynth search**

A1	Medial superior prefrontal cortex	-2, 14, 52	346	Terms="frontoparietal network"; "attention", Number of studies=1526
A2	Lateral prefrontal cortex L	-44, 6, 32	287	Terms="frontoparietal network"; "attention", Number of studies=1526
A3	Lateral prefrontal cortex R	50, 10, 28	255	Terms="frontoparietal network"; "attention", Number of studies=1526
A4	Anterior inferior parietal lobule L	-30, -54, 40	249	Terms="frontoparietal network"; "attention", Number of studies=1526
A5	Anterior inferior parietal lobule R	38, -56, 48	312	Terms="frontoparietal network"; "attention", Number of studies=1526,
A6	Precuneus L	-14, -66, 52	302	Terms="frontoparietal network"; "attention", Number of studies=1526,
A7	Precuneus R	18, -68, 52	380	Terms="frontoparietal network"; "attention", Number of studies=1526,
**Computed features**	**Anatomical combinations**	**Input metrics**	**Condition**	**Task contrast**
A2A1	Lateral prefrontal cortex L with medial superior prefrontal cortex	Connectivity	Task-free	None
A3A1	Lateral prefrontal cortex R with medial superior prefrontal cortex	Connectivity	Task-free	None
A4A2	Anterior inferior parietal lobule L with lateral prefrontal cortex L	Connectivity	Task-free	None
A5A3	Anterior inferior parietal lobule R with lateral prefrontal cortex R	Connectivity	Task-free	None
A4A6	Anterior inferior parietal lobule L with precuneus L	Connectivity	Task-free	None
A5A7	Anterior inferior parietal lobule R with precuneus R	Connectivity	Task-free	None
**Negative affect circuit (sad)**				
**Region label**	**Region anatomy**	**Atlas/MNI coordinates**	**Voxels**	**Neurosynth search**
N1	Pregenual anterior cingulate	6, 42, 4	136	Terms="threat", "sad", Number of studies=170,
N2	Anterior insula L	-36, 20, -4	481	Terms="threat", "sad", Number of studies=170,
N3	Anterior insula R	38, 22, -4	463	Terms="threat", "sad", Number of studies=170,
N4	Amygdala L	AAL	217	Terms="threat", "sad", Number of studies=170,
N5	Amygdala R	AAL	225	Terms="threat", "sad", Number of studies=170,
**Computed features**	**Anatomical combinations**	**Input metrics**	**Condition**	**Task contrast**
NS1	Pregenual anterior cingulate	Activation	Conscious Facial Emotion Viewing	Sad vs Neutral evoked by facial emotion stimuli
NS2	Anterior insula L	Activation	Conscious Facial Emotion Viewing	Sad vs Neutral evoked by facial emotion stimuli

NS3	Anterior insula R	Activation	Conscious Facial Emotion Viewing	Sad vs Neutral evoked by facial emotion stimuli
NS4	Amygdala L	Activation	Conscious Facial Emotion Viewing	Sad vs Neutral evoked by facial emotion stimuli
NS5	Amygdala R	Activation	Conscious Facial Emotion Viewing	Sad vs Neutral evoked by facial emotion stimuli
NS2NS1	(anterior insula L to pregenual anterior cingulate + pregenual anterior cingulate to anterior insula L)/2	Connectivity	Conscious Facial Emotion Viewing	Sad vs Neutral evoked by facial emotion stimuli
NS3NS1	(anterior insula R to pregenual anterior cingulate + pregenual anterior cingulate to anterior insula R)/2	Connectivity	Conscious Facial Emotion Viewing	Sad vs Neutral evoked by facial emotion stimuli
NS4NS1	(amygdala L to pregenual anterior cingulate + pregenual anterior cingulate to amygdala L)/2	Connectivity	Conscious Facial Emotion Viewing	Sad vs Neutral evoked by facial emotion stimuli
NS5NS1	(amygdala R to pregenual anterior cingulate + pregenual anterior cingulate to amygdala R)/2	Connectivity	Conscious Facial Emotion Viewing	Sad vs Neutral evoked by facial emotion stimuli
**Negative affect circuit (threat)**				
**Region label**	**Region anatomy**	**Atlas/MNI coordinates**	**Voxels**	**Neurosynth search**
NT1	Dorsal anterior cingulate	6, 22, 32	279	Terms="threat", "sad", Number of studies=170,
NT2	Amygdala L	AAL	217	Terms="threat", "sad", Number of studies=170,
NT3	Amygdala R	AAL	225	Terms="threat", "sad", Number of studies=170,
**Computed features**	**Anatomical combinations**	**Input metrics**	**Condition**	**Task contrast**
NT1	Dorsal anterior cingulate	Activation	Conscious Facial Emotion Viewing	Fear/Anger vs Neutral evoked by facial emotion stimuli
NT2	Amygdala L	Activation	Conscious Facial Emotion Viewing	Fear/Anger vs Neutral evoked by facial emotion stimuli
NT3	Amygdala R	Activation	Conscious Facial Emotion Viewing	Fear/Anger vs Neutral evoked by facial emotion stimuli
NT2NT1	(amygdala L to dorsal anterior cingulate + dorsal anterior cingulate to amygdala L)/2	Connectivity	Conscious Facial Emotion Viewing	Fear/Anger vs Neutral evoked by facial emotion stimuli
NT3NT1	(amygdala R to dorsal anterior cingulate + dorsal anterior cingulate to amygdala R)/2	Connectivity	Conscious Facial Emotion Viewing	Fear/Anger vs Neutral evoked by facial emotion stimuli
NTN1	Dorsal anterior cingulate	Activation	Nonconscious Facial Emotion Viewing	Fear/Anger vs Neutral evoked by facial emotion stimuli
NTN2	Amygdala L	Activation	Nonconscious Facial Emotion Viewing	Fear/Anger vs Neutral evoked by facial emotion stimuli
NTN3	Amygdala R	Activation	Nonconscious Facial Emotion Viewing	Fear/Anger vs Neutral evoked by facial emotion stimuli

NTN2NTN1	(amygdala L to dorsal anterior cingulate + dorsal anterior cingulate to amygdala L)/2	Connectivity	Nonconscious Facial Emotion Viewing	Fear/Anger vs Neutral evoked by facial emotion stimuli
NTN3NTN1	(amygdala R to dorsal anterior cingulate + dorsal anterior cingulate to amygdala R)/2	Connectivity	Nonconscious Facial Emotion Viewing	Fear/Anger vs Neutral evoked by facial emotion stimuli
**Positive affect circuit**				
**Region label**	**Region anatomy**	**Atlas/MNI coordinates**	**Voxels**	**Neurosynth search**
P1	Ventral medial prefrontal cortex	-2, 56, -8	408	Terms="monetary reward"; "reward", Number of studies=755, Search date = 6.4.17
P2	Ventral striatum L	Harvard-Oxford	179	Terms="monetary reward"; "reward", Number of studies=755, Search date = 6.4.17
P3	Ventral striatum R	Harvard-Oxford	174	Terms="monetary reward"; "reward", Number of studies=755, Search date = 6.4.17
**Computed features**	**Anatomical combinations**	**Input metrics**	**Condition**	**Task contrast**
P1	Ventral medial prefrontal cortex	Activation	Conscious Facial Emotion Viewing	Happy vs Neutral evoked by facial emotion stimuli
P2	Ventral striatum L	Activation	Conscious Facial Emotion Viewing	Happy vs Neutral evoked by facial emotion stimuli
P3	Ventral striatum R	Activation	Conscious Facial Emotion Viewing	Happy vs Neutral evoked by facial emotion stimuli
**Cognitive control circuit**				
**Region label**	**Region anatomy**	**Atlas/MNI coordinates**	**Voxels**	**Neurosynth search**
C1	Dorsal anterior cingulate	0, 18, 46	514	Terms="cognitive control", Number of studies=428, Search date = 6.4.17
C2	Dorsal lateral prefrontal cortex/inferior frontal gyrus L	-44, 6, 32	501	Terms="cognitive control", Number of studies=428, Search date = 6.4.17
C3	Dorsal lateral prefrontal cortex/ inferior frontal gyrus R	44, 34, 22	412	Terms="cognitive control", Number of studies=428, Search date = 6.4.17
**Computed features**	**Anatomical combinations**	**Input metrics**	**Condition**	**Task contrast**
C1	Dorsal anterior cingulate	Activation	Go-NoGo task	No-Go vs Go
C2	Dorsal lateral prefrontal cortex/inferior frontal gyrus L	Activation	Go-NoGo task	No-Go vs Go
C3	Dorsal lateral prefrontal cortex/ inferior frontal gyrus R	Activation	Go-NoGo task	No-Go vs Go
C1C2	(dorsal anterior cingulate to dorsal lateral prefrontal cortex/inferior frontal gyrus L + dorsal lateral prefrontal cortex/inferior frontal gyrus L to dorsal anterior cingulate)/2	Connectivity	Go-NoGo task	No-Go vs Go
C3C2	(dorsal anterior cingulate to dorsal lateral prefrontal cortex/inferior frontal gyrus R + dorsal lateral prefrontal cortex/inferior frontal gyrus R to dorsal anterior cingulate)/2	Connectivity	Go-NoGo task	No-Go vs Go

Supplementary Table 19: Number of symptom and behavioral measures in the cross-sectional analyses. We show how many participants had data available for each measure in each of the studies. The instrument from which each measure was derived is indicated in parentheses. Equivalent behavioral measures were derived from WebNeuro (RAD, HCP-DES, ENGAGE) and IntegNeuro (iSPOT-D). *Abbreviations*: SHAPS=Snaith-Hamilton Pleasure Scale, MASQ=Mood and Anxiety Questionnaire, BIS= Barratt Impulsiveness Scale, DASS=Depression Anxiety and Stress Scale, RRS=Ruminative Response Scale, PSWQ= Penn State Worry Questionnaire-Abbreviated, QIDS=Quick Inventory of Depressive Symptomatology Self-Report Revised, WN=WebNeuro, IN=IntegNeuro.

**Features**	**Clinical Sample 1**	**Clinical Sample 2**	**Clinical Sample 3**	**Clinical Sample** 4
Dataset	RAD	HCP-DES	iSPOT-D	ENGAGE
Symptoms				
Anhedonia (SHAPS)	0	208	0	0
Anxious arousal (MASQ)	308	208	0	0
Cognitive dyscontrol (BIS)	308	208	0	0
Negative bias (DASS)	289	208	0	189
Ruminative brooding (RRS)	0	208	0	0
Ruminative worry (PSWQ)	309	0	0	0
Sleep (QIDS)	289	208	0	197
Tension (DASS)	289	208	0	187
Threat dysregulation (DASS)	289	208	0	189
Behavioral measures				
Explicit happy RT (WN/IN)	308	188	80	93
Explicit sad RT (WN/IN)	308	188	80	93
Explicit threat RT (WN/IN)	308	188	80	93
Explicit happy RT (WN/IN)	308	188	80	93
Explicit sad RT (WN/IN)	308	188	80	93
Explicit threat RT (WN/IN)	308	188	80	93
Go-NoGo commission errors (WN/IN)	302	188	80	93
Go-NoGo mean RT (WN/IN)	301	188	79	93
Implicit happy RT (WN/IN)	308	188	80	93
Implicit sad RT (WN/IN)	308	188	80	93
Implicit threat RT (WN/IN)	307	188	80	93
Maze completion time (WN/IN)	306	188	79	93
Maze errors (WN/IN)	306	188	79	93
Working memory commission errors (WN/IN)	304	187	79	93
Working memory omission errors (WN/IN)	304	187	80	93
Working memory RT (WN/IN)	304	187	79	93

References

1. Korgaonkar, M. S., Ram, K., Williams, L. M., Gatt, J. M. & Grieve, S. M. Establishing the resting state default mode network derived from functional magnetic resonance imaging tasks as an endophenotype: A twins study. *Hum Brain Mapp* **35**, 3893–3902 (2014).
2. Korgaonkar, M. S., Grieve, S. M., Etkin, A., Koslow, S. H. & Williams, L. M. Using standardized fMRI protocols to identify patterns of prefrontal circuit dysregulation that are common and specific to cognitive and emotional tasks in major depressive disorder: first wave results from the iSPOT-D study. *Neuropsychopharmacology* **38**, 863–871 (2013).
3. Williams, L. M. *et al.* Amygdala-prefrontal dissociation of subliminal and supraliminal fear. *Hum Brain Mapp* **27**, 652–661 (2006).
4. Grieve, S. M. *et al.* Brain imaging predictors and the international study to predict optimized treatment for depression: study protocol for a randomized controlled trial. *Trials* **14**, 224 (2013).
5. Esteban, O. *et al.* fMRIPrep: a robust preprocessing pipeline for functional MRI. *Nat. Methods* **16**, 111–116 (2019).
6. Goldstein-Piekarski, A. N. *et al.* Mapping Neural Circuit Biotypes to Symptoms and Behavioral Dimensions of Depression and Anxiety. *Biological Psychiatry* **91**, 561–571 (2022).
7. Yarkoni, T., Poldrack, R. A., Nichols, T. E., Van Essen, D. C. & Wager, T. D. Large-scale automated synthesis of human functional neuroimaging data. *Nat Methods* **8**, 665–670 (2011).
8. Tzourio-Mazoyer, N. *et al.* Automated anatomical labeling of activations in SPM using a macroscopic anatomical parcellation of the MNI MRI single-subject brain. *Neuroimage* **15**, 273–289 (2002).
9. Tziortzi, A. C. *et al.* Imaging dopamine receptors in humans with [11C]-(+)-PHNO: dissection of D3 signal and anatomy. *Neuroimage* **54**, 264–277 (2011).
10. Williams, L. M. Precision psychiatry: a neural circuit taxonomy for depression and anxiety. *Lancet Psychiatry* **3**, 472–480 (2016).
11. Jenkinson, M., Beckmann, C. F., Behrens, T. E. J., Woolrich, M. W. & Smith, S. M. FSL. *Neuroimage* **62**, 782–790 (2012).
12. Drysdale, A. T. *et al.* Resting-state connectivity biomarkers define neurophysiological subtypes of depression. *Nat Med* **23**, 28–38 (2017).
13. Liang, S. *et al.* Biotypes of major depressive disorder: Neuroimaging evidence from resting-state default mode network patterns. *NeuroImage: Clinical* **28**, 102514 (2020).
14. Tokuda, T. *et al.* Identification of depression subtypes and relevant brain regions using a data-driven approach. *Sci Rep* **8**, 14082 (2018).

iSPOT-D FMRIPREP PROCESSING DETAILS

Results included in this manuscript come from preprocessing performed using *fMRIPrep* 20.2.1 (Esteban, Markiewicz, et al. (2018); Esteban, Blair, et al. (2018); RRID:SCR_016216), which is based on *Nipype* 1.5.1 (Gorgolewski et al. (2011); Gorgolewski et al. (2018); RRID:SCR_002502).

Anatomical data preprocessing
A total of 1 T1-weighted (T1w) images were found within the input BIDS dataset.The T1-weighted (T1w) image was corrected for intensity non-uniformity (INU) with N4BiasFieldCorrection (Tustison et al. 2010), distributed with ANTs 2.3.3 (Avants et al. 2008, RRID:SCR_004757), and used as T1w-reference throughout the workflow. The T1w-reference was then skull-stripped with a *Nipype* implementation of the antsBrainExtraction.sh workflow (from ANTs), using OASIS30ANTs as target template. Brain tissue segmentation of cerebrospinal fluid (CSF), white-matter (WM) and gray-matter (GM) was performed on the brain-extracted T1w using fast (FSL 5.0.9, RRID:SCR_002823, Zhang, Brady, and Smith 2001). Brain surfaces were reconstructed using recon-all (FreeSurfer 6.0.1, RRID:SCR_001847, Dale, Fischl, and Sereno 1999), and the brain mask estimated previously was refined with a custom variation of the method to reconcile ANTs-derived and FreeSurfer-derived segmentations of the cortical gray-matter of Mindboggle (RRID:SCR_002438, Klein et al. 2017). Volume-based spatial normalization to two standard spaces (MNI152NLin2009cAsym, MNI152NLin6Asym) was performed through nonlinear registration with antsRegistration (ANTs 2.3.3), using brain-extracted versions of both T1w reference and the T1w template. The following templates were selected for spatial normalization: *ICBM 152 Nonlinear Asymmetrical template version 2009c* [Fonov et al. (2009), RRID:SCR_008796; TemplateFlow ID: MNI152NLin2009cAsym], *FSL's MNI ICBM 152 non-linear 6th Generation Asymmetric Average Brain Stereotaxic Registration Model* [Evans et al. (2012), RRID:SCR_002823; TemplateFlow ID: MNI152NLin6Asym],

Functional data preprocessing
For each of the 10 BOLD runs found per subject (across all tasks and sessions), the following preprocessing was performed. First, a reference volume and its skull-stripped version were generated using a custom methodology of *fMRIPrep* . Susceptibility distortion correction (SDC) was omitted. The BOLD reference was then co-registered to the T1w reference using bbregister (FreeSurfer) which implements boundary-based registration (Greve and Fischl 2009). Co-registration was configured with six degrees of freedom. Head-motion parameters with respect to the BOLD reference (transformation matrices, and six corresponding rotation and translation parameters) are estimated before any spatiotemporal filtering using mcflirt (FSL 5.0.9, Jenkinson et al. 2002). BOLD runs were slice-time corrected using 3dTshift from AFNI 20160207 (Cox and Hyde 1997, RRID:SCR_005927). The BOLD time-series were resampled onto the following surfaces (FreeSurfer reconstruction nomenclature): *fsnative*, *fsaverage*. The BOLD time-series (including slice-timing correction when applied) were resampled onto their original, native space by applying the transforms to correct for head-motion. These resampled BOLD time-series will be referred to as *preprocessed BOLD in original space*, or just *preprocessed BOLD*. The BOLD time-series were resampled into standard space, generating a *preprocessed BOLD run in MNI152NLin2009cAsym space*. First, a reference volume and its skull-stripped version were generated using a custom methodology of *fMRIPrep* . *Grayordinates* files (Glasser et al. 2013) containing 91k samples were also generated using the highest-resolution fsaverage as intermediate standardized surface space. Automatic removal of motion artifacts using independent component analysis (ICA-AROMA, Pruim et al. 2015) was performed on the *preprocessed BOLD on MNI space* time-series after removal of non-steady state volumes and spatial smoothing with an isotropic, Gaussian kernel of 6mm FWHM (full-width half-maximum). Corresponding "non-aggresively" denoised runs were produced after such smoothing. Additionally, the "aggressive" noise-regressors were collected and placed in the corresponding confounds file. Several confounding time-series were calculated based on the *preprocessed BOLD*: framewise displacement (FD), DVARS and three region-wise global signals. FD was computed using two formulations following Power

(absolute sum of relative motions, Power et al. (2014)) and Jenkinson (relative root mean square displacement between affines, Jenkinson et al. (2002)). FD and DVARS are calculated for each functional run, both using their implementations in *Nipype* (following the definitions by Power et al. 2014). The three global signals are extracted within the CSF, the WM, and the whole-brain masks. Additionally, a set of physiological regressors were extracted to allow for component-based noise correction (*CompCor*, Behzadi et al. 2007). Principal components are estimated after high-pass filtering the *preprocessed BOLD* time-series (using a discrete cosine filter with 128s cut-off) for the two *CompCor* variants: temporal (tCompCor) and anatomical (aCompCor). tCompCor components are then calculated from the top 2% variable voxels within the brain mask. For aCompCor, three probabilistic masks (CSF, WM and combined CSF+WM) are generated in anatomical space. The implementation differs from that of Behzadi et al. in that instead of eroding the masks by 2 pixels on BOLD space, the aCompCor masks are subtracted a mask of pixels that likely contain a volume fraction of GM. This mask is obtained by dilating a GM mask extracted from the FreeSurfer's *aseg* segmentation, and it ensures components are not extracted from voxels containing a minimal fraction of GM. Finally, these masks are resampled into BOLD space and binarized by thresholding at 0.99 (as in the original implementation). Components are also calculated separately within the WM and CSF masks. For each CompCor decomposition, the *k* components with the largest singular values are retained, such that the retained components' time series are sufficient to explain 50 percent of variance across the nuisance mask (CSF, WM, combined, or temporal). The remaining components are dropped from consideration. The head-motion estimates calculated in the correction step were also placed within the corresponding confounds file. The confound time series derived from head motion estimates and global signals were expanded with the inclusion of temporal derivatives and quadratic terms for each (Satterthwaite et al. 2013). Frames that exceeded a threshold of 0.5 mm FD or 1.5 standardised DVARS were annotated as motion outliers. All resamplings can be performed with *a single interpolation step* by composing all the pertinent transformations (i.e. head-motion transform matrices, susceptibility distortion correction when available, and co-registrations to anatomical and output spaces). Gridded (volumetric) resamplings were performed using antsApplyTransforms (ANTs), configured with Lanczos interpolation to minimize the smoothing effects of other kernels (Lanczos 1964). Non-gridded (surface) resamplings were performed using mri_vol2surf (FreeSurfer).

Many internal operations of *fMRIPrep* use *Nilearn* 0.6.2 (Abraham et al. 2014, RRID:SCR_001362), mostly within the functional processing workflow. For more details of the pipeline, see the section corresponding to workflows in *fMRIPrep* 's documentation.

Copyright Waiver

The above boilerplate text was automatically generated by fMRIPrep with the express intention that users should copy and paste this text into their manuscripts *unchanged*. It is released under the CC0 license.

References

Abraham, Alexandre, Fabian Pedregosa, Michael Eickenberg, Philippe Gervais, Andreas Mueller, Jean Kossaifi, Alexandre Gramfort, Bertrand Thirion, and Gael Varoquaux. 2014. "Machine Learning for Neuroimaging with Scikit-Learn." *Frontiers in Neuroinformatics* 8. https://doi.org/10.3389/fninf.2014.00014.

Avants, B.B., C.L. Epstein, M. Grossman, and J.C. Gee. 2008. "Symmetric Diffeomorphic Image Registration with Cross-Correlation: Evaluating Automated Labeling of Elderly and Neurodegenerative Brain." *Medical Image Analysis* 12 (1): 26–41. https://doi.org/10.1016/j.media.2007.06.004.

Behzadi, Yashar, Khaled Restom, Joy Liau, and Thomas T. Liu. 2007. "A Component Based Noise Correction Method (CompCor) for BOLD and Perfusion Based fMRI." *NeuroImage* 37 (1): 90–101. https://doi.org/10.1016/j.neuroimage.2007.04.042.

Cox, Robert W., and James S. Hyde. 1997. “Software Tools for Analysis and Visualization of fMRI Data.” *NMR in Biomedicine* 10 (4-5): 171–78. https://doi.org/10.1002/(SICI)1099-1492(199706/08)10:4/5<171::AID-NBM453>3.0.CO;2-L.

Dale, Anders M., Bruce Fischl, and Martin I. Sereno. 1999. “Cortical Surface-Based Analysis: I. Segmentation and Surface Reconstruction.” *NeuroImage* 9 (2): 179–94. https://doi.org/10.1006/nimg.1998.0395.

Esteban, Oscar, Ross Blair, Christopher J. Markiewicz, Shoshana L. Berleant, Craig Moodie, Feilong Ma, Ayse Ilkay Isik, et al. 2018. “FMRIPrep .” *Software*. Zenodo. https://doi.org/10.5281/zenodo.852659.

Esteban, Oscar, Christopher Markiewicz, Ross W Blair, Craig Moodie, Ayse Ilkay Isik, Asier Erramuzpe Aliaga, James Kent, et al. 2018. “fMRIPrep : A Robust Preprocessing Pipeline for Functional MRI.” *Nature Methods*. https://doi.org/10.1038/s41592-018-0235-4.

Evans, AC, AL Janke, DL Collins, and S Baillet. 2012. “Brain Templates and Atlases.” *NeuroImage* 62 (2): 911–22. https://doi.org/10.1016/j.neuroimage.2012.01.024.

Fonov, VS, AC Evans, RC McKinstry, CR Almli, and DL Collins. 2009. “Unbiased Nonlinear Average Age-Appropriate Brain Templates from Birth to Adulthood.” *NeuroImage* 47, Supplement 1: S102. https://doi.org/10.1016/S1053-8119(09)70884-5.

Glasser, Matthew F., Stamatios N. Sotiropoulos, J. Anthony Wilson, Timothy S. Coalson, Bruce Fischl, Jesper L. Andersson, Junqian Xu, et al. 2013. “The Minimal Preprocessing Pipelines for the Human Connectome Project.” *NeuroImage*, Mapping the connectome, 80: 105–24. https://doi.org/10.1016/j.neuroimage.2013.04.127.

Gorgolewski, K., C. D. Burns, C. Madison, D. Clark, Y. O. Halchenko, M. L. Waskom, and S. Ghosh. 2011. “Nipype: A Flexible, Lightweight and Extensible Neuroimaging Data Processing Framework in Python.” *Frontiers in Neuroinformatics* 5: 13. https://doi.org/10.3389/fninf.2011.00013.

Gorgolewski, Krzysztof J., Oscar Esteban, Christopher J. Markiewicz, Erik Ziegler, David Gage Ellis, Michael Philipp Notter, Dorota Jarecka, et al. 2018. “Nipype.” *Software*. Zenodo. https://doi.org/10.5281/zenodo.596855.

Greve, Douglas N, and Bruce Fischl. 2009. “Accurate and Robust Brain Image Alignment Using Boundary-Based Registration.” *NeuroImage* 48 (1): 63–72. https://doi.org/10.1016/j.neuroimage.2009.06.060.

Jenkinson, Mark, Peter Bannister, Michael Brady, and Stephen Smith. 2002. “Improved Optimization for the Robust and Accurate Linear Registration and Motion Correction of Brain Images.” *NeuroImage* 17 (2): 825–41. https://doi.org/10.1006/nimg.2002.1132.

Klein, Arno, Satrajit S. Ghosh, Forrest S. Bao, Joachim Giard, Yrjö Häme, Eliezer Stavsky, Noah Lee, et al. 2017. “Mindboggling Morphometry of Human Brains.” *PLOS Computational Biology* 13 (2): e1005350. https://doi.org/10.1371/journal.pcbi.1005350.

Lanczos, C. 1964. “Evaluation of Noisy Data.” *Journal of the Society for Industrial and Applied Mathematics Series B Numerical Analysis* 1 (1): 76–85. https://doi.org/10.1137/0701007.

Power, Jonathan D., Anish Mitra, Timothy O. Laumann, Abraham Z. Snyder, Bradley L. Schlaggar, and Steven E. Petersen. 2014. "Methods to Detect, Characterize, and Remove Motion Artifact in Resting State fMRI." *NeuroImage* 84 (Supplement C): 320–41. https://doi.org/10.1016/j.neuroimage.2013.08.048.

Pruim, Raimon H. R., Maarten Mennes, Daan van Rooij, Alberto Llera, Jan K. Buitelaar, and Christian F. Beckmann. 2015. "ICA-AROMA: A Robust ICA-Based Strategy for Removing Motion Artifacts from fMRI Data." *NeuroImage* 112 (Supplement C): 267–77. https://doi.org/10.1016/j.neuroimage.2015.02.064.

Satterthwaite, Theodore D., Mark A. Elliott, Raphael T. Gerraty, Kosha Ruparel, James Loughead, Monica E. Calkins, Simon B. Eickhoff, et al. 2013. "An improved framework for confound regression and filtering for control of motion artifact in the preprocessing of resting-state functional connectivity data." *NeuroImage* 64 (1): 240–56. https://doi.org/10.1016/j.neuroimage.2012.08.052.

Tustison, N. J., B. B. Avants, P. A. Cook, Y. Zheng, A. Egan, P. A. Yushkevich, and J. C. Gee. 2010. "N4ITK: Improved N3 Bias Correction." *IEEE Transactions on Medical Imaging* 29 (6): 1310–20. https://doi.org/10.1109/TMI.2010.2046908.

Zhang, Y., M. Brady, and S. Smith. 2001. "Segmentation of Brain MR Images Through a Hidden Markov Random Field Model and the Expectation-Maximization Algorithm." *IEEE Transactions on Medical Imaging* 20 (1): 45–57. https://doi.org/10.1109/42.906424.

RAD fMRIPREP PROCESSING DETAILS

Results included in this manuscript come from preprocessing performed using *fMRIPrep* 20.2.3 (Esteban, Markiewicz, et al. (2018); Esteban, Blair, et al. (2018); RRID:SCR_016216), which is based on *Nipype* 1.6.1 (Gorgolewski et al. (2011); Gorgolewski et al. (2018); RRID:SCR_002502).

Anatomical data preprocessing

A total of 1 T1-weighted (T1w) images were found within the input BIDS dataset.The T1-weighted (T1w) image was corrected for intensity non-uniformity (INU) with N4BiasFieldCorrection (Tustison et al. 2010), distributed with ANTs 2.3.3 (Avants et al. 2008, RRID:SCR_004757), and used as T1w-reference throughout the workflow. The T1w-reference was then skull-stripped with a *Nipype* implementation of the antsBrainExtraction.sh workflow (from ANTs), using OASIS30ANTs as target template. Brain tissue segmentation of cerebrospinal fluid (CSF), white-matter (WM) and gray-matter (GM) was performed on the brain-extracted T1w using fast (FSL 5.0.9, RRID:SCR_002823, Zhang, Brady, and Smith 2001). Brain surfaces were reconstructed using recon-all (FreeSurfer 6.0.1, RRID:SCR_001847, Dale, Fischl, and Sereno 1999), and the brain mask estimated previously was refined with a custom variation of the method to reconcile ANTs-derived and FreeSurfer-derived segmentations of the cortical gray-matter of Mindboggle (RRID:SCR_002438, Klein et al. 2017). Volume-based spatial normalization to two standard spaces (MNI152NLin6Asym, MNI152NLin2009cAsym) was performed through nonlinear registration with antsRegistration (ANTs 2.3.3), using brain-extracted versions of both T1w reference and the T1w template. The following templates were selected for spatial normalization: *FSL's MNI ICBM 152 non-linear 6th Generation Asymmetric Average Brain Stereotaxic Registration Model* [Evans et al. (2012), RRID:SCR_002823; TemplateFlow ID: MNI152NLin6Asym], *ICBM 152 Nonlinear Asymmetrical template version 2009c* [Fonov et al. (2009), RRID:SCR_008796; TemplateFlow ID: MNI152NLin2009cAsym],

Functional data preprocessing

For each of the 4 BOLD runs found per subject (across all tasks and sessions), the following preprocessing was performed. First, a reference volume and its skull-stripped version were generated using a custom methodology of *fMRIPrep* . Susceptibility distortion correction (SDC) was omitted. The BOLD reference was then co-registered to the T1w reference using bbregister (FreeSurfer) which implements boundary-based registration (Greve and Fischl 2009). Co-registration was configured with six degrees of freedom. Head-motion parameters with respect to the BOLD reference (transformation matrices, and six corresponding rotation and translation parameters) are estimated before any spatiotemporal filtering using mcflirt (FSL 5.0.9, Jenkinson et al. 2002). BOLD runs were slice-time corrected using 3dTshift from AFNI 20160207 (Cox and Hyde 1997, RRID:SCR_005927). The BOLD time-series were resampled onto the following surfaces (FreeSurfer reconstruction nomenclature): *fsnative*, *fsaverage*. The BOLD time-series (including slice-timing correction when applied) were resampled onto their original, native space by applying the transforms to correct for head-motion. These resampled BOLD time-series will be referred to as *preprocessed BOLD in original space*, or just *preprocessed BOLD*. The BOLD time-series were resampled into standard space, generating a *preprocessed BOLD run in MNI152NLin6Asym space*. First, a reference volume and its skull-stripped version were generated using a custom methodology of *fMRIPrep* . *Grayordinates* files (Glasser et al. 2013) containing 91k samples were also generated using the highest-resolution fsaverage as intermediate standardized surface space. Automatic removal of motion artifacts using independent component analysis (ICA-AROMA, Pruim et al. 2015) was performed on the *preprocessed BOLD on MNI space* time-series after removal of non-steady state volumes and spatial smoothing with an isotropic, Gaussian kernel of 6mm FWHM (full-width half-maximum). Corresponding "non-aggresively" denoised runs were produced after such smoothing. Additionally, the "aggressive" noise-regressors were collected and placed in the corresponding confounds file. Several confounding time-series were calculated based on the *preprocessed BOLD*: framewise displacement (FD), DVARS and three region-wise global signals. FD was computed using two formulations following Power

(absolute sum of relative motions, Power et al. (2014)) and Jenkinson (relative root mean square displacement between affines, Jenkinson et al. (2002)). FD and DVARS are calculated for each functional run, both using their implementations in *Nipype* (following the definitions by Power et al. 2014). The three global signals are extracted within the CSF, the WM, and the whole-brain masks. Additionally, a set of physiological regressors were extracted to allow for component-based noise correction (*CompCor*, Behzadi et al. 2007). Principal components are estimated after high-pass filtering the *preprocessed BOLD* time-series (using a discrete cosine filter with 128s cut-off) for the two *CompCor* variants: temporal (tCompCor) and anatomical (aCompCor). tCompCor components are then calculated from the top 2% variable voxels within the brain mask. For aCompCor, three probabilistic masks (CSF, WM and combined CSF+WM) are generated in anatomical space. The implementation differs from that of Behzadi et al. in that instead of eroding the masks by 2 pixels on BOLD space, the aCompCor masks are subtracted a mask of pixels that likely contain a volume fraction of GM. This mask is obtained by dilating a GM mask extracted from the FreeSurfer's *aseg* segmentation, and it ensures components are not extracted from voxels containing a minimal fraction of GM. Finally, these masks are resampled into BOLD space and binarized by thresholding at 0.99 (as in the original implementation). Components are also calculated separately within the WM and CSF masks. For each CompCor decomposition, the *k* components with the largest singular values are retained, such that the retained components' time series are sufficient to explain 50 percent of variance across the nuisance mask (CSF, WM, combined, or temporal). The remaining components are dropped from consideration. The head-motion estimates calculated in the correction step were also placed within the corresponding confounds file. The confound time series derived from head motion estimates and global signals were expanded with the inclusion of temporal derivatives and quadratic terms for each (Satterthwaite et al. 2013). Frames that exceeded a threshold of 0.5 mm FD or 1.5 standardised DVARS were annotated as motion outliers. All resamplings can be performed with *a single interpolation step* by composing all the pertinent transformations (i.e. head-motion transform matrices, susceptibility distortion correction when available, and co-registrations to anatomical and output spaces). Gridded (volumetric) resamplings were performed using antsApplyTransforms (ANTs), configured with Lanczos interpolation to minimize the smoothing effects of other kernels (Lanczos 1964). Non-gridded (surface) resamplings were performed using mri_vol2surf (FreeSurfer).

Many internal operations of *fMRIPrep* use *Nilearn* 0.6.2 (Abraham et al. 2014, RRID:SCR_001362), mostly within the functional processing workflow. For more details of the pipeline, see the section corresponding to workflows in *fMRIPrep* 's documentation.

Copyright Waiver

The above boilerplate text was automatically generated by fMRIPrep with the express intention that users should copy and paste this text into their manuscripts *unchanged*. It is released under the CC0 license.

References

Abraham, Alexandre, Fabian Pedregosa, Michael Eickenberg, Philippe Gervais, Andreas Mueller, Jean Kossaifi, Alexandre Gramfort, Bertrand Thirion, and Gael Varoquaux. 2014. "Machine Learning for Neuroimaging with Scikit-Learn." *Frontiers in Neuroinformatics* 8. https://doi.org/10.3389/fninf.2014.00014.

Avants, B.B., C.L. Epstein, M. Grossman, and J.C. Gee. 2008. "Symmetric Diffeomorphic Image Registration with Cross-Correlation: Evaluating Automated Labeling of Elderly and Neurodegenerative Brain." *Medical Image Analysis* 12 (1): 26–41. https://doi.org/10.1016/j.media.2007.06.004.

Behzadi, Yashar, Khaled Restom, Joy Liau, and Thomas T. Liu. 2007. "A Component Based Noise Correction Method (CompCor) for BOLD and Perfusion Based fMRI." *NeuroImage* 37 (1): 90–101. https://doi.org/10.1016/j.neuroimage.2007.04.042.

Cox, Robert W., and James S. Hyde. 1997. "Software Tools for Analysis and Visualization of fMRI Data." *NMR in Biomedicine* 10 (4-5): 171–78. https://doi.org/10.1002/(SICI)1099-1492(199706/08)10:4/5<171::AID-NBM453>3.0.CO;2-L.

Dale, Anders M., Bruce Fischl, and Martin I. Sereno. 1999. "Cortical Surface-Based Analysis: I. Segmentation and Surface Reconstruction." *NeuroImage* 9 (2): 179–94. https://doi.org/10.1006/nimg.1998.0395.

Esteban, Oscar, Ross Blair, Christopher J. Markiewicz, Shoshana L. Berleant, Craig Moodie, Feilong Ma, Ayse Ilkay Isik, et al. 2018. "FMRIPrep ." *Software*. Zenodo. https://doi.org/10.5281/zenodo.852659.

Esteban, Oscar, Christopher Markiewicz, Ross W Blair, Craig Moodie, Ayse Ilkay Isik, Asier Erramuzpe Aliaga, James Kent, et al. 2018. "fMRIPrep : A Robust Preprocessing Pipeline for Functional MRI." *Nature Methods*. https://doi.org/10.1038/s41592-018-0235-4.

Evans, AC, AL Janke, DL Collins, and S Baillet. 2012. "Brain Templates and Atlases." *NeuroImage* 62 (2): 911–22. https://doi.org/10.1016/j.neuroimage.2012.01.024.

Fonov, VS, AC Evans, RC McKinstry, CR Almli, and DL Collins. 2009. "Unbiased Nonlinear Average Age-Appropriate Brain Templates from Birth to Adulthood." *NeuroImage* 47, Supplement 1: S102. https://doi.org/10.1016/S1053-8119(09)70884-5.

Glasser, Matthew F., Stamatios N. Sotiropoulos, J. Anthony Wilson, Timothy S. Coalson, Bruce Fischl, Jesper L. Andersson, Junqian Xu, et al. 2013. "The Minimal Preprocessing Pipelines for the Human Connectome Project." *NeuroImage*, Mapping the connectome, 80: 105–24. https://doi.org/10.1016/j.neuroimage.2013.04.127.

Gorgolewski, K., C. D. Burns, C. Madison, D. Clark, Y. O. Halchenko, M. L. Waskom, and S. Ghosh. 2011. "Nipype: A Flexible, Lightweight and Extensible Neuroimaging Data Processing Framework in Python." *Frontiers in Neuroinformatics* 5: 13. https://doi.org/10.3389/fninf.2011.00013.

Gorgolewski, Krzysztof J., Oscar Esteban, Christopher J. Markiewicz, Erik Ziegler, David Gage Ellis, Michael Philipp Notter, Dorota Jarecka, et al. 2018. "Nipype." *Software*. Zenodo. https://doi.org/10.5281/zenodo.596855.

Greve, Douglas N, and Bruce Fischl. 2009. "Accurate and Robust Brain Image Alignment Using Boundary-Based Registration." *NeuroImage* 48 (1): 63–72. https://doi.org/10.1016/j.neuroimage.2009.06.060.

Jenkinson, Mark, Peter Bannister, Michael Brady, and Stephen Smith. 2002. "Improved Optimization for the Robust and Accurate Linear Registration and Motion Correction of Brain Images." *NeuroImage* 17 (2): 825–41. https://doi.org/10.1006/nimg.2002.1132.

Klein, Arno, Satrajit S. Ghosh, Forrest S. Bao, Joachim Giard, Yrjö Häme, Eliezer Stavsky, Noah Lee, et al. 2017. "Mindboggling Morphometry of Human Brains." *PLOS Computational Biology* 13 (2): e1005350. https://doi.org/10.1371/journal.pcbi.1005350.

Lanczos, C. 1964. "Evaluation of Noisy Data." *Journal of the Society for Industrial and Applied Mathematics Series B Numerical Analysis* 1 (1): 76–85. https://doi.org/10.1137/0701007.

Power, Jonathan D., Anish Mitra, Timothy O. Laumann, Abraham Z. Snyder, Bradley L. Schlaggar, and Steven E. Petersen. 2014. "Methods to Detect, Characterize, and Remove Motion Artifact in Resting State fMRI." *NeuroImage* 84 (Supplement C): 320–41. https://doi.org/10.1016/j.neuroimage.2013.08.048.

Pruim, Raimon H. R., Maarten Mennes, Daan van Rooij, Alberto Llera, Jan K. Buitelaar, and Christian F. Beckmann. 2015. "ICA-AROMA: A Robust ICA-Based Strategy for Removing Motion Artifacts from fMRI Data." *NeuroImage* 112 (Supplement C): 267–77. https://doi.org/10.1016/j.neuroimage.2015.02.064.

Satterthwaite, Theodore D., Mark A. Elliott, Raphael T. Gerraty, Kosha Ruparel, James Loughead, Monica E. Calkins, Simon B. Eickhoff, et al. 2013. "An improved framework for confound regression and filtering for control of motion artifact in the preprocessing of resting-state functional connectivity data." *NeuroImage* 64 (1): 240–56. https://doi.org/10.1016/j.neuroimage.2012.08.052.

Tustison, N. J., B. B. Avants, P. A. Cook, Y. Zheng, A. Egan, P. A. Yushkevich, and J. C. Gee. 2010. "N4ITK: Improved N3 Bias Correction." *IEEE Transactions on Medical Imaging* 29 (6): 1310–20. https://doi.org/10.1109/TMI.2010.2046908.

Zhang, Y., M. Brady, and S. Smith. 2001. "Segmentation of Brain MR Images Through a Hidden Markov Random Field Model and the Expectation-Maximization Algorithm." *IEEE Transactions on Medical Imaging* 20 (1): 45–57. https://doi.org/10.1109/42.906424.

ENGAGE fMRIPREP PROCESSING DETAILS

Results included in this manuscript come from preprocessing performed using *fMRIPrep* 20.2.3 (Esteban, Markiewicz, et al. (2018); Esteban, Blair, et al. (2018); RRID:SCR_016216), which is based on *Nipype* 1.6.1 (Gorgolewski et al. (2011); Gorgolewski et al. (2018); RRID:SCR_002502).

Anatomical data preprocessing

A total of 4 T1-weighted (T1w) images were found within the input BIDS dataset. All of them were corrected for intensity non-uniformity (INU) with N4BiasFieldCorrection (Tustison et al. 2010), distributed with ANTs 2.3.3 (Avants et al. 2008, RRID:SCR_004757). The T1w-reference was then skull-stripped with a *Nipype* implementation of the antsBrainExtraction.sh workflow (from ANTs), using OASIS30ANTs as target template. Brain tissue segmentation of cerebrospinal fluid (CSF), white-matter (WM) and gray-matter (GM) was performed on the brain-extracted T1w using fast (FSL 5.0.9, RRID:SCR_002823, Zhang, Brady, and Smith 2001). A T1w-reference map was computed after registration of 4 T1w images (after INU-correction) using mri_robust_template (FreeSurfer 6.0.1, Reuter, Rosas, and Fischl 2010). Brain surfaces were reconstructed using recon-all (FreeSurfer 6.0.1, RRID:SCR_001847, Dale, Fischl, and Sereno 1999), and the brain mask estimated previously was refined with a custom variation of the method to reconcile ANTs-derived and FreeSurfer-derived segmentations of the cortical gray-matter of Mindboggle (RRID:SCR_002438, Klein et al. 2017). Volume-based spatial normalization to two standard spaces (MNI152NLin2009cAsym, MNI152NLin6Asym) was performed through nonlinear registration with antsRegistration (ANTs 2.3.3), using brain-extracted versions of both T1w reference and the T1w template. The following templates were selected for spatial normalization: *ICBM 152 Nonlinear Asymmetrical template version 2009c* [Fonov et al. (2009), RRID:SCR_008796; TemplateFlow ID: MNI152NLin2009cAsym], *FSL's MNI ICBM 152 non-linear 6th Generation Asymmetric Average Brain Stereotaxic Registration Model* [Evans et al. (2012), RRID:SCR_002823; TemplateFlow ID: MNI152NLin6Asym],

Functional data preprocessing

For each of the 21 BOLD runs found per subject (across all tasks and sessions), the following preprocessing was performed. First, a reference volume and its skull-stripped version were generated using a custom methodology of *fMRIPrep* . Susceptibility distortion correction (SDC) was omitted. The BOLD reference was then co-registered to the T1w reference using bbregister (FreeSurfer) which implements boundary-based registration (Greve and Fischl 2009). Co-registration was configured with six degrees of freedom. Head-motion parameters with respect to the BOLD reference (transformation matrices, and six corresponding rotation and translation parameters) are estimated before any spatiotemporal filtering using mcflirt (FSL 5.0.9, Jenkinson et al. 2002). BOLD runs were slice-time corrected using 3dTshift from AFNI 20160207 (Cox and Hyde 1997, RRID:SCR_005927). The BOLD time-series were resampled onto the following surfaces (FreeSurfer reconstruction nomenclature): *fsnative*, *fsaverage*. The BOLD time-series (including slice-timing correction when applied) were resampled onto their original, native space by applying the transforms to correct for head-motion. These resampled BOLD time-series will be referred to as *preprocessed BOLD in original space*, or just *preprocessed BOLD*. The BOLD time-series were resampled into standard space, generating a *preprocessed BOLD run in MNI152NLin2009cAsym space*. First, a reference volume and its skull-stripped version were generated using a custom methodology of *fMRIPrep* . *Grayordinates* files (Glasser et al. 2013) containing 91k samples were also generated using the highest-resolution fsaverage as intermediate standardized surface space. Automatic removal of motion artifacts using independent component analysis (ICA-AROMA, Pruim et al. 2015) was performed on the *preprocessed BOLD on MNI space* time-series after removal of non-steady state volumes and spatial smoothing with an isotropic, Gaussian kernel of 6mm FWHM (full-width half-maximum). Corresponding “non-aggresively” denoised runs were produced after such smoothing. Additionally, the “aggressive” noise-regressors were collected and placed in the corresponding confounds file. Several confounding time-series were calculated based on the *preprocessed BOLD*: framewise displacement

(FD), DVARS and three region-wise global signals. FD was computed using two formulations following Power (absolute sum of relative motions, Power et al. (2014)) and Jenkinson (relative root mean square displacement between affines, Jenkinson et al. (2002)). FD and DVARS are calculated for each functional run, both using their implementations in *Nipype* (following the definitions by Power et al. 2014). The three global signals are extracted within the CSF, the WM, and the whole-brain masks. Additionally, a set of physiological regressors were extracted to allow for component-based noise correction (*CompCor*, Behzadi et al. 2007). Principal components are estimated after high-pass filtering the *preprocessed BOLD* time-series (using a discrete cosine filter with 128s cut-off) for the two *CompCor* variants: temporal (tCompCor) and anatomical (aCompCor). tCompCor components are then calculated from the top 2% variable voxels within the brain mask. For aCompCor, three probabilistic masks (CSF, WM and combined CSF+WM) are generated in anatomical space. The implementation differs from that of Behzadi et al. in that instead of eroding the masks by 2 pixels on BOLD space, the aCompCor masks are subtracted a mask of pixels that likely contain a volume fraction of GM. This mask is obtained by dilating a GM mask extracted from the FreeSurfer's *aseg* segmentation, and it ensures components are not extracted from voxels containing a minimal fraction of GM. Finally, these masks are resampled into BOLD space and binarized by thresholding at 0.99 (as in the original implementation). Components are also calculated separately within the WM and CSF masks. For each CompCor decomposition, the *k* components with the largest singular values are retained, such that the retained components' time series are sufficient to explain 50 percent of variance across the nuisance mask (CSF, WM, combined, or temporal). The remaining components are dropped from consideration. The head-motion estimates calculated in the correction step were also placed within the corresponding confounds file. The confound time series derived from head motion estimates and global signals were expanded with the inclusion of temporal derivatives and quadratic terms for each (Satterthwaite et al. 2013). Frames that exceeded a threshold of 0.5 mm FD or 1.5 standardised DVARS were annotated as motion outliers. All resamplings can be performed with *a single interpolation step* by composing all the pertinent transformations (i.e. head-motion transform matrices, susceptibility distortion correction when available, and co-registrations to anatomical and output spaces). Gridded (volumetric) resamplings were performed using antsApplyTransforms (ANTs), configured with Lanczos interpolation to minimize the smoothing effects of other kernels (Lanczos 1964). Non-gridded (surface) resamplings were performed using mri_vol2surf (FreeSurfer).

Many internal operations of *fMRIPrep* use *Nilearn* 0.6.2 (Abraham et al. 2014, RRID:SCR_001362), mostly within the functional processing workflow. For more details of the pipeline, see the section corresponding to workflows in *fMRIPrep* 's documentation.

Copyright Waiver

The above boilerplate text was automatically generated by fMRIPrep with the express intention that users should copy and paste this text into their manuscripts *unchanged*. It is released under the CC0 license.

References

Abraham, Alexandre, Fabian Pedregosa, Michael Eickenberg, Philippe Gervais, Andreas Mueller, Jean Kossaifi, Alexandre Gramfort, Bertrand Thirion, and Gael Varoquaux. 2014. "Machine Learning for Neuroimaging with Scikit-Learn." *Frontiers in Neuroinformatics* 8. https://doi.org/10.3389/fninf.2014.00014.

Avants, B.B., C.L. Epstein, M. Grossman, and J.C. Gee. 2008. "Symmetric Diffeomorphic Image Registration with Cross-Correlation: Evaluating Automated Labeling of Elderly and Neurodegenerative Brain." *Medical Image Analysis* 12 (1): 26–41. https://doi.org/10.1016/j.media.2007.06.004.

Behzadi, Yashar, Khaled Restom, Joy Liau, and Thomas T. Liu. 2007. "A Component Based Noise Correction Method (CompCor) for BOLD and Perfusion Based fMRI." *NeuroImage* 37 (1): 90–101. https://doi.org/10.1016/j.neuroimage.2007.04.042.

Cox, Robert W., and James S. Hyde. 1997. "Software Tools for Analysis and Visualization of fMRI Data." *NMR in Biomedicine* 10 (4-5): 171–78. https://doi.org/10.1002/(SICI)1099-1492(199706/08)10:4/5<171::AID-NBM453>3.0.CO;2-L.

Dale, Anders M., Bruce Fischl, and Martin I. Sereno. 1999. "Cortical Surface-Based Analysis: I. Segmentation and Surface Reconstruction." *NeuroImage* 9 (2): 179–94. https://doi.org/10.1006/nimg.1998.0395.

Esteban, Oscar, Ross Blair, Christopher J. Markiewicz, Shoshana L. Berleant, Craig Moodie, Feilong Ma, Ayse Ilkay Isik, et al. 2018. "FMRIPrep ." *Software*. Zenodo. https://doi.org/10.5281/zenodo.852659.

Esteban, Oscar, Christopher Markiewicz, Ross W Blair, Craig Moodie, Ayse Ilkay Isik, Asier Erramuzpe Aliaga, James Kent, et al. 2018. "fMRIPrep : A Robust Preprocessing Pipeline for Functional MRI." *Nature Methods*. https://doi.org/10.1038/s41592-018-0235-4.

Evans, AC, AL Janke, DL Collins, and S Baillet. 2012. "Brain Templates and Atlases." *NeuroImage* 62 (2): 911–22. https://doi.org/10.1016/j.neuroimage.2012.01.024.

Fonov, VS, AC Evans, RC McKinstry, CR Almli, and DL Collins. 2009. "Unbiased Nonlinear Average Age-Appropriate Brain Templates from Birth to Adulthood." *NeuroImage* 47, Supplement 1: S102. https://doi.org/10.1016/S1053-8119(09)70884-5.

Glasser, Matthew F., Stamatios N. Sotiropoulos, J. Anthony Wilson, Timothy S. Coalson, Bruce Fischl, Jesper L. Andersson, Junqian Xu, et al. 2013. "The Minimal Preprocessing Pipelines for the Human Connectome Project." *NeuroImage*, Mapping the connectome, 80: 105–24. https://doi.org/10.1016/j.neuroimage.2013.04.127.

Gorgolewski, K., C. D. Burns, C. Madison, D. Clark, Y. O. Halchenko, M. L. Waskom, and S. Ghosh. 2011. "Nipype: A Flexible, Lightweight and Extensible Neuroimaging Data Processing Framework in Python." *Frontiers in Neuroinformatics* 5: 13. https://doi.org/10.3389/fninf.2011.00013.

Gorgolewski, Krzysztof J., Oscar Esteban, Christopher J. Markiewicz, Erik Ziegler, David Gage Ellis, Michael Philipp Notter, Dorota Jarecka, et al. 2018. "Nipype." *Software*. Zenodo. https://doi.org/10.5281/zenodo.596855.

Greve, Douglas N, and Bruce Fischl. 2009. "Accurate and Robust Brain Image Alignment Using Boundary-Based Registration." *NeuroImage* 48 (1): 63–72. https://doi.org/10.1016/j.neuroimage.2009.06.060.

Jenkinson, Mark, Peter Bannister, Michael Brady, and Stephen Smith. 2002. "Improved Optimization for the Robust and Accurate Linear Registration and Motion Correction of Brain Images." *NeuroImage* 17 (2): 825–41. https://doi.org/10.1006/nimg.2002.1132.

Klein, Arno, Satrajit S. Ghosh, Forrest S. Bao, Joachim Giard, Yrjö Häme, Eliezer Stavsky, Noah Lee, et al. 2017. "Mindboggling Morphometry of Human Brains." *PLOS Computational Biology* 13 (2): e1005350. https://doi.org/10.1371/journal.pcbi.1005350.

Lanczos, C. 1964. “Evaluation of Noisy Data.” *Journal of the Society for Industrial and Applied Mathematics Series B Numerical Analysis* 1 (1): 76–85. https://doi.org/10.1137/0701007.

Power, Jonathan D., Anish Mitra, Timothy O. Laumann, Abraham Z. Snyder, Bradley L. Schlaggar, and Steven E. Petersen. 2014. “Methods to Detect, Characterize, and Remove Motion Artifact in Resting State fMRI.” *NeuroImage* 84 (Supplement C): 320–41. https://doi.org/10.1016/j.neuroimage.2013.08.048.

Pruim, Raimon H. R., Maarten Mennes, Daan van Rooij, Alberto Llera, Jan K. Buitelaar, and Christian F. Beckmann. 2015. “ICA-AROMA: A Robust ICA-Based Strategy for Removing Motion Artifacts from fMRI Data.” *NeuroImage* 112 (Supplement C): 267–77. https://doi.org/10.1016/j.neuroimage.2015.02.064.

Reuter, Martin, Herminia Diana Rosas, and Bruce Fischl. 2010. “Highly Accurate Inverse Consistent Registration: A Robust Approach.” *NeuroImage* 53 (4): 1181–96. https://doi.org/10.1016/j.neuroimage.2010.07.020.

Satterthwaite, Theodore D., Mark A. Elliott, Raphael T. Gerraty, Kosha Ruparel, James Loughead, Monica E. Calkins, Simon B. Eickhoff, et al. 2013. “An improved framework for confound regression and filtering for control of motion artifact in the preprocessing of resting-state functional connectivity data.” *NeuroImage* 64 (1): 240–56. https://doi.org/10.1016/j.neuroimage.2012.08.052.

Tustison, N. J., B. B. Avants, P. A. Cook, Y. Zheng, A. Egan, P. A. Yushkevich, and J. C. Gee. 2010. “N4ITK: Improved N3 Bias Correction.” *IEEE Transactions on Medical Imaging* 29 (6): 1310–20. https://doi.org/10.1109/TMI.2010.2046908.

Zhang, Y., M. Brady, and S. Smith. 2001. “Segmentation of Brain MR Images Through a Hidden Markov Random Field Model and the Expectation-Maximization Algorithm.” *IEEE Transactions on Medical Imaging* 20 (1): 45–57. https://doi.org/10.1109/42.906424.

HCP-DES fMRIPREP PROCESSING DETAILS

Results included in this manuscript come from preprocessing performed using *fMRIPrep* 20.2.3 (Esteban, Markiewicz, et al. (2018); Esteban, Blair, et al. (2018); RRID:SCR_016216), which is based on *Nipype* 1.6.1 (Gorgolewski et al. (2011); Gorgolewski et al. (2018); RRID:SCR_002502).

Anatomical data preprocessing
A total of 1 T1-weighted (T1w) images were found within the input BIDS dataset.The T1-weighted (T1w) image was corrected for intensity non-uniformity (INU) with N4BiasFieldCorrection (Tustison et al. 2010), distributed with ANTs 2.3.3 (Avants et al. 2008, RRID:SCR_004757), and used as T1w-reference throughout the workflow. The T1w-reference was then skull-stripped with a *Nipype* implementation of the antsBrainExtraction.sh workflow (from ANTs), using OASIS30ANTs as target template. Brain tissue segmentation of cerebrospinal fluid (CSF), white-matter (WM) and gray-matter (GM) was performed on the brain-extracted T1w using fast (FSL 5.0.9, RRID:SCR_002823, Zhang, Brady, and Smith 2001). Brain surfaces were reconstructed using recon-all (FreeSurfer 6.0.1, RRID:SCR_001847, Dale, Fischl, and Sereno 1999), and the brain mask estimated previously was refined with a custom variation of the method to reconcile ANTs-derived and FreeSurfer-derived segmentations of the cortical gray-matter of Mindboggle (RRID:SCR_002438, Klein et al. 2017). Volume-based spatial normalization to two standard spaces (MNI152NLin6Asym, MNI152NLin2009cAsym) was performed through nonlinear registration with antsRegistration (ANTs 2.3.3), using brain-extracted versions of both T1w reference and the T1w template. The following templates were selected for spatial normalization: *FSL's MNI ICBM 152 non-linear 6th Generation Asymmetric Average Brain Stereotaxic Registration Model* [Evans et al. (2012), RRID:SCR_002823; TemplateFlow ID: MNI152NLin6Asym], *ICBM 152 Nonlinear Asymmetrical template version 2009c* [Fonov et al. (2009), RRID:SCR_008796; TemplateFlow ID: MNI152NLin2009cAsym],

Functional data preprocessing
For each of the 6 BOLD runs found per subject (across all tasks and sessions), the following preprocessing was performed. First, a reference volume and its skull-stripped version were generated using a custom methodology of *fMRIPrep* . Susceptibility distortion correction (SDC) was omitted. The BOLD reference was then co-registered to the T1w reference using bbregister (FreeSurfer) which implements boundary-based registration (Greve and Fischl 2009). Co-registration was configured with six degrees of freedom. Head-motion parameters with respect to the BOLD reference (transformation matrices, and six corresponding rotation and translation parameters) are estimated before any spatiotemporal filtering using mcflirt (FSL 5.0.9, Jenkinson et al. 2002). BOLD runs were slice-time corrected using 3dTshift from AFNI 20160207 (Cox and Hyde 1997, RRID:SCR_005927). The BOLD time-series were resampled onto the following surfaces (FreeSurfer reconstruction nomenclature): *fsnative*, *fsaverage*. The BOLD time-series (including slice-timing correction when applied) were resampled onto their original, native space by applying the transforms to correct for head-motion. These resampled BOLD time-series will be referred to as *preprocessed BOLD in original space*, or just *preprocessed BOLD*. The BOLD time-series were resampled into standard space, generating a *preprocessed BOLD run in MNI152NLin6Asym space*. First, a reference volume and its skull-stripped version were generated using a custom methodology of *fMRIPrep* . *Grayordinates* files (Glasser et al. 2013) containing 91k samples were also generated using the highest-resolution fsaverage as intermediate standardized surface space. Automatic removal of motion artifacts using independent component analysis (ICA-AROMA, Pruim et al. 2015) was performed on the *preprocessed BOLD on MNI space* time-series after removal of non-steady state volumes and spatial smoothing with an isotropic, Gaussian kernel of 6mm FWHM (full-width half-maximum). Corresponding "non-aggresively" denoised runs were produced after such smoothing. Additionally, the "aggressive" noise-regressors were collected and placed in the corresponding confounds file. Several confounding time-series were calculated based on the *preprocessed BOLD*: framewise displacement (FD), DVARS and three region-wise global signals. FD was computed using two formulations following Power

(absolute sum of relative motions, Power et al. (2014)) and Jenkinson (relative root mean square displacement between affines, Jenkinson et al. (2002)). FD and DVARS are calculated for each functional run, both using their implementations in *Nipype* (following the definitions by Power et al. 2014). The three global signals are extracted within the CSF, the WM, and the whole-brain masks. Additionally, a set of physiological regressors were extracted to allow for component-based noise correction (*CompCor*, Behzadi et al. 2007). Principal components are estimated after high-pass filtering the *preprocessed BOLD* time-series (using a discrete cosine filter with 128s cut-off) for the two *CompCor* variants: temporal (tCompCor) and anatomical (aCompCor). tCompCor components are then calculated from the top 2% variable voxels within the brain mask. For aCompCor, three probabilistic masks (CSF, WM and combined CSF+WM) are generated in anatomical space. The implementation differs from that of Behzadi et al. in that instead of eroding the masks by 2 pixels on BOLD space, the aCompCor masks are subtracted a mask of pixels that likely contain a volume fraction of GM. This mask is obtained by dilating a GM mask extracted from the FreeSurfer's *aseg* segmentation, and it ensures components are not extracted from voxels containing a minimal fraction of GM. Finally, these masks are resampled into BOLD space and binarized by thresholding at 0.99 (as in the original implementation). Components are also calculated separately within the WM and CSF masks. For each CompCor decomposition, the *k* components with the largest singular values are retained, such that the retained components' time series are sufficient to explain 50 percent of variance across the nuisance mask (CSF, WM, combined, or temporal). The remaining components are dropped from consideration. The head-motion estimates calculated in the correction step were also placed within the corresponding confounds file. The confound time series derived from head motion estimates and global signals were expanded with the inclusion of temporal derivatives and quadratic terms for each (Satterthwaite et al. 2013). Frames that exceeded a threshold of 0.5 mm FD or 1.5 standardised DVARS were annotated as motion outliers. All resamplings can be performed with *a single interpolation step* by composing all the pertinent transformations (i.e. head-motion transform matrices, susceptibility distortion correction when available, and co-registrations to anatomical and output spaces). Gridded (volumetric) resamplings were performed using antsApplyTransforms (ANTs), configured with Lanczos interpolation to minimize the smoothing effects of other kernels (Lanczos 1964). Non-gridded (surface) resamplings were performed using mri_vol2surf (FreeSurfer). First, a reference volume and its skull-stripped version were generated using a custom methodology of *fMRIPrep* . A B0-nonuniformity map (or *fieldmap*) was estimated based on two (or more) echo-planar imaging (EPI) references with opposing phase-encoding directions, with 3dQwarp Cox and Hyde (1997) (AFNI 20160207). Based on the estimated susceptibility distortion, a corrected EPI (echo-planar imaging) reference was calculated for a more accurate co-registration with the anatomical reference. The BOLD reference was then co-registered to the T1w reference using bbregister (FreeSurfer) which implements boundary-based registration (Greve and Fischl 2009). Co-registration was configured with six degrees of freedom. Head-motion parameters with respect to the BOLD reference (transformation matrices, and six corresponding rotation and translation parameters) are estimated before any spatiotemporal filtering using mcflirt (FSL 5.0.9, Jenkinson et al. 2002). BOLD runs were slice-time corrected using 3dTshift from AFNI 20160207 (Cox and Hyde 1997, RRID:SCR_005927). The BOLD time-series were resampled onto the following surfaces (FreeSurfer reconstruction nomenclature): *fsnative*, *fsaverage*. The BOLD time-series (including slice-timing correction when applied) were resampled onto their original, native space by applying a single, composite transform to correct for head-motion and susceptibility distortions. These resampled BOLD time-series will be referred to as *preprocessed BOLD in original space*, or just *preprocessed BOLD*. The BOLD time-series were resampled into standard space, generating a *preprocessed BOLD run in MNI152NLin6Asym space*. First, a reference volume and its skull-stripped version were generated using a custom methodology of *fMRIPrep* . *Grayordinates* files (Glasser et al. 2013) containing 91k samples were also generated using the highest-resolution fsaverage as intermediate standardized surface space. Automatic removal of motion artifacts using independent component analysis (ICA-AROMA, Pruim et al. 2015) was performed on the *preprocessed BOLD on MNI space* time-series after removal of non-steady state volumes and spatial smoothing with an isotropic, Gaussian kernel of 6mm FWHM (full-width half-maximum). Corresponding "non-aggresively" denoised runs were produced after such smoothing. Additionally, the "aggressive" noise-regressors were collected and placed in the corresponding confounds file.

Several confounding time-series were calculated based on the *preprocessed BOLD*: framewise displacement (FD), DVARS and three region-wise global signals. FD was computed using two formulations following Power (absolute sum of relative motions, Power et al. (2014)) and Jenkinson (relative root mean square displacement between affines, Jenkinson et al. (2002)). FD and DVARS are calculated for each functional run, both using their implementations in *Nipype* (following the definitions by Power et al. 2014). The three global signals are extracted within the CSF, the WM, and the whole-brain masks. Additionally, a set of physiological regressors were extracted to allow for component-based noise correction (*CompCor*, Behzadi et al. 2007). Principal components are estimated after high-pass filtering the *preprocessed BOLD* time-series (using a discrete cosine filter with 128s cut-off) for the two *CompCor* variants: temporal (tCompCor) and anatomical (aCompCor). tCompCor components are then calculated from the top 2% variable voxels within the brain mask. For aCompCor, three probabilistic masks (CSF, WM and combined CSF+WM) are generated in anatomical space. The implementation differs from that of Behzadi et al. in that instead of eroding the masks by 2 pixels on BOLD space, the aCompCor masks are subtracted a mask of pixels that likely contain a volume fraction of GM. This mask is obtained by dilating a GM mask extracted from the FreeSurfer's *aseg* segmentation, and it ensures components are not extracted from voxels containing a minimal fraction of GM. Finally, these masks are resampled into BOLD space and binarized by thresholding at 0.99 (as in the original implementation). Components are also calculated separately within the WM and CSF masks. For each CompCor decomposition, the *k* components with the largest singular values are retained, such that the retained components' time series are sufficient to explain 50 percent of variance across the nuisance mask (CSF, WM, combined, or temporal). The remaining components are dropped from consideration. The head-motion estimates calculated in the correction step were also placed within the corresponding confounds file. The confound time series derived from head motion estimates and global signals were expanded with the inclusion of temporal derivatives and quadratic terms for each (Satterthwaite et al. 2013). Frames that exceeded a threshold of 0.5 mm FD or 1.5 standardised DVARS were annotated as motion outliers. All resamplings can be performed with *a single interpolation step* by composing all the pertinent transformations (i.e. head-motion transform matrices, susceptibility distortion correction when available, and co-registrations to anatomical and output spaces). Gridded (volumetric) resamplings were performed using antsApplyTransforms (ANTs), configured with Lanczos interpolation to minimize the smoothing effects of other kernels (Lanczos 1964). Non-gridded (surface) resamplings were performed using mri_vol2surf (FreeSurfer).

Many internal operations of *fMRIPrep* use *Nilearn* 0.6.2 (Abraham et al. 2014, RRID:SCR_001362), mostly within the functional processing workflow. For more details of the pipeline, see the section corresponding to workflows in *fMRIPrep* 's documentation.

Copyright Waiver

The above boilerplate text was automatically generated by fMRIPrep with the express intention that users should copy and paste this text into their manuscripts *unchanged*. It is released under the CC0 license.

References

Abraham, Alexandre, Fabian Pedregosa, Michael Eickenberg, Philippe Gervais, Andreas Mueller, Jean Kossaifi, Alexandre Gramfort, Bertrand Thirion, and Gael Varoquaux. 2014. "Machine Learning for Neuroimaging with Scikit-Learn." *Frontiers in Neuroinformatics* 8. https://doi.org/10.3389/fninf.2014.00014.

Avants, B.B., C.L. Epstein, M. Grossman, and J.C. Gee. 2008. "Symmetric Diffeomorphic Image Registration with Cross-Correlation: Evaluating Automated Labeling of Elderly and Neurodegenerative Brain." *Medical Image Analysis* 12 (1): 26–41. https://doi.org/10.1016/j.media.2007.06.004.

Behzadi, Yashar, Khaled Restom, Joy Liau, and Thomas T. Liu. 2007. "A Component Based Noise Correction Method (CompCor) for BOLD and Perfusion Based fMRI." *NeuroImage* 37 (1): 90–101. https://doi.org/10.1016/j.neuroimage.2007.04.042.

Cox, Robert W., and James S. Hyde. 1997. "Software Tools for Analysis and Visualization of fMRI Data." *NMR in Biomedicine* 10 (4-5): 171–78. https://doi.org/10.1002/(SICI)1099-1492(199706/08)10:4/5<171::AID-NBM453>3.0.CO;2-L.

Dale, Anders M., Bruce Fischl, and Martin I. Sereno. 1999. "Cortical Surface-Based Analysis: I. Segmentation and Surface Reconstruction." *NeuroImage* 9 (2): 179–94. https://doi.org/10.1006/nimg.1998.0395.

Esteban, Oscar, Ross Blair, Christopher J. Markiewicz, Shoshana L. Berleant, Craig Moodie, Feilong Ma, Ayse Ilkay Isik, et al. 2018. "FMRIPrep ." *Software*. Zenodo. https://doi.org/10.5281/zenodo.852659.

Esteban, Oscar, Christopher Markiewicz, Ross W Blair, Craig Moodie, Ayse Ilkay Isik, Asier Erramuzpe Aliaga, James Kent, et al. 2018. "fMRIPrep : A Robust Preprocessing Pipeline for Functional MRI." *Nature Methods*. https://doi.org/10.1038/s41592-018-0235-4.

Evans, AC, AL Janke, DL Collins, and S Baillet. 2012. "Brain Templates and Atlases." *NeuroImage* 62 (2): 911–22. https://doi.org/10.1016/j.neuroimage.2012.01.024.

Fonov, VS, AC Evans, RC McKinstry, CR Almli, and DL Collins. 2009. "Unbiased Nonlinear Average Age-Appropriate Brain Templates from Birth to Adulthood." *NeuroImage* 47, Supplement 1: S102. https://doi.org/10.1016/S1053-8119(09)70884-5.

Glasser, Matthew F., Stamatios N. Sotiropoulos, J. Anthony Wilson, Timothy S. Coalson, Bruce Fischl, Jesper L. Andersson, Junqian Xu, et al. 2013. "The Minimal Preprocessing Pipelines for the Human Connectome Project." *NeuroImage*, Mapping the connectome, 80: 105–24. https://doi.org/10.1016/j.neuroimage.2013.04.127.

Gorgolewski, K., C. D. Burns, C. Madison, D. Clark, Y. O. Halchenko, M. L. Waskom, and S. Ghosh. 2011. "Nipype: A Flexible, Lightweight and Extensible Neuroimaging Data Processing Framework in Python." *Frontiers in Neuroinformatics* 5: 13. https://doi.org/10.3389/fninf.2011.00013.

Gorgolewski, Krzysztof J., Oscar Esteban, Christopher J. Markiewicz, Erik Ziegler, David Gage Ellis, Michael Philipp Notter, Dorota Jarecka, et al. 2018. "Nipype." *Software*. Zenodo. https://doi.org/10.5281/zenodo.596855.

Greve, Douglas N, and Bruce Fischl. 2009. "Accurate and Robust Brain Image Alignment Using Boundary-Based Registration." *NeuroImage* 48 (1): 63–72. https://doi.org/10.1016/j.neuroimage.2009.06.060.

Jenkinson, Mark, Peter Bannister, Michael Brady, and Stephen Smith. 2002. "Improved Optimization for the Robust and Accurate Linear Registration and Motion Correction of Brain Images." *NeuroImage* 17 (2): 825–41. https://doi.org/10.1006/nimg.2002.1132.

Klein, Arno, Satrajit S. Ghosh, Forrest S. Bao, Joachim Giard, Yrjö Häme, Eliezer Stavsky, Noah Lee, et al. 2017. "Mindboggling Morphometry of Human Brains." *PLOS Computational Biology* 13 (2): e1005350. https://doi.org/10.1371/journal.pcbi.1005350.

Lanczos, C. 1964. “Evaluation of Noisy Data.” *Journal of the Society for Industrial and Applied Mathematics Series B Numerical Analysis* 1 (1): 76–85. https://doi.org/10.1137/0701007.

Power, Jonathan D., Anish Mitra, Timothy O. Laumann, Abraham Z. Snyder, Bradley L. Schlaggar, and Steven E. Petersen. 2014. “Methods to Detect, Characterize, and Remove Motion Artifact in Resting State fMRI.” *NeuroImage* 84 (Supplement C): 320–41. https://doi.org/10.1016/j.neuroimage.2013.08.048.

Pruim, Raimon H. R., Maarten Mennes, Daan van Rooij, Alberto Llera, Jan K. Buitelaar, and Christian F. Beckmann. 2015. “ICA-AROMA: A Robust ICA-Based Strategy for Removing Motion Artifacts from fMRI Data.” *NeuroImage* 112 (Supplement C): 267–77. https://doi.org/10.1016/j.neuroimage.2015.02.064.

Satterthwaite, Theodore D., Mark A. Elliott, Raphael T. Gerraty, Kosha Ruparel, James Loughead, Monica E. Calkins, Simon B. Eickhoff, et al. 2013. “An improved framework for confound regression and filtering for control of motion artifact in the preprocessing of resting-state functional connectivity data.” *NeuroImage* 64 (1): 240–56. https://doi.org/10.1016/j.neuroimage.2012.08.052.

Tustison, N. J., B. B. Avants, P. A. Cook, Y. Zheng, A. Egan, P. A. Yushkevich, and J. C. Gee. 2010. “N4ITK: Improved N3 Bias Correction.” *IEEE Transactions on Medical Imaging* 29 (6): 1310–20. https://doi.org/10.1109/TMI.2010.2046908.

Zhang, Y., M. Brady, and S. Smith. 2001. “Segmentation of Brain MR Images Through a Hidden Markov Random Field Model and the Expectation-Maximization Algorithm.” *IEEE Transactions on Medical Imaging* 20 (1): 45–57. https://doi.org/10.1109/42.906424.
