# Supplementary material for: Personalized brain circuit scores identify clinically distinct biotypes in depression and anxiety
Source: Nat Med. 2024 Jun 17;30(7):2076–87. doi: 10.1038/s41591-024-03057-9 (PMC11271415; doi:10.1038/s41591-024-03057-9)
Supplement: Supplementary file 2 — Reporting Summary [file 41591_2024_3057_MOESM2_ESM.pdf]

Corresponding author(s): Leanne M. Williams

Last updated by author(s): May 3, 2024

Reporting Summary

Nature Portfolio wishes to improve the reproducibility of the work that we publish. This form provides structure for consistency and transparency in reporting. For further information on Nature Portfolio policies, see our Editorial Policies and the Editorial Policy Checklist.

Statistics

For all statistical analyses, confirm that the following items are present in the figure legend, table legend, main text, or Methods section.

n/a	Confirmed	
☐	☒	The exact sample size (n) for each experimental group/condition, given as a discrete number and unit of measurement
☐	☒	A statement on whether measurements were taken from distinct samples or whether the same sample was measured repeatedly
☐	☒	The statistical test(s) used AND whether they are one- or two-sided<br>*Only common tests should be described solely by name; describe more complex techniques in the Methods section.*
☐	☒	A description of all covariates tested
☐	☒	A description of any assumptions or corrections, such as tests of normality and adjustment for multiple comparisons
☐	☒	A full description of the statistical parameters including central tendency (e.g. means) or other basic estimates (e.g. regression coefficient) AND variation (e.g. standard deviation) or associated estimates of uncertainty (e.g. confidence intervals)
☐	☒	For null hypothesis testing, the test statistic (e.g. F, t, r) with confidence intervals, effect sizes, degrees of freedom and P value noted<br>*Give P values as exact values whenever suitable.*
☒	☐	For Bayesian analysis, information on the choice of priors and Markov chain Monte Carlo settings
☐	☒	For hierarchical and complex designs, identification of the appropriate level for tests and full reporting of outcomes
☐	☒	Estimates of effect sizes (e.g. Cohen's d, Pearson's r), indicating how they were calculated

Our web collection on statistics for biologists contains articles on many of the points above.

Software and code

Policy information about availability of computer code

Data collection	No software was used.
Data analysis	The following open source software was used: SPM version 8, FSL version 6, MATLAB version 2018b, fMRIprep version 20.2.1, R version 4.1.3, R Studio version 2023.06.2+561, R package miceRanger version 1.5.0. Custom software was also used and is available on Github at https://github.com/leotozzi88/cluster_study_2023.

For manuscripts utilizing custom algorithms or software that are central to the research but not yet described in published literature, software must be made available to editors and reviewers. We strongly encourage code deposition in a community repository (e.g. GitHub). See the Nature Portfolio guidelines for submitting code & software for further information.

Data

Policy information about availability of data

All manuscripts must include a data availability statement. This statement should provide the following information, where applicable:

- Accession codes, unique identifiers, or web links for publicly available datasets
- A description of any restrictions on data availability
- For clinical datasets or third party data, please ensure that the statement adheres to our policy

The datasets used in this analysis were collected as part of the iSPOT-D, RAD, HCP-DES and ENGAGE studies. These datasets are available upon request from Stanford BrainNet at https://www.stanfordpmhw.com/datasets. The BRAINnet repository meets the requirements for being public but also aligns with the

procedures of other official public and scientific repositories like HCP, ABCD, and NDA. This choice is in line with the FAIRness guidelines, and it respects the original funding requirements, allowing for appropriate source contributions and citations. Our approach is specifically designed for scientific use, which includes limiting access to for-profit entities to comply with the original funding stipulations and participant consent. Therefore, total open access is not feasible. Our intention is to provide public access that is consistent with the consent agreements and the original funding intentions, similar to the data shared through NIH repositories. On Stanford BRAINnet, we established a data access request form that screens users, similar to other public repositories.

Research involving human participants, their data, or biological material

Policy information about studies with human participants or human data. See also policy information about sex, gender (identity/presentation), and sexual orientation and race, ethnicity and racism.

Reporting on sex and gender	Self-reported gender was collected and used for the analyses.
Reporting on race, ethnicity, or other socially relevant groupings	Self-reported race and tehnicity was collected but not used for the analyses.
Population characteristics	The data used in this paper were obtained from four studies: “International Study to Predict Optimized Treatment in Depression” (iSPOT-D, (Williams et al., 2011)), “Research on Anxiety and Depression study” (RAD, (Williams et al., 2016)), “Human Connectome Project for Disordered Emotional States” (HCP-DES, (Tozzi et al., 2020c)), and “Engaging self-regulation targets to understand the mechanisms of behavior change and improve mood and weight outcome” (ENGAGE, (Williams et al., 2018)).<br><br>Clinical participants:<br>Gender:<br>Female: 461 (58%)<br>Male: 329 (41%)<br>Other: 11 (1%) 0 (0%)<br>Age mean (standard deviation): 34.24 (13.40)<br><br>Controls<br>Gender:<br>Female: 67 (49%)<br>Male: 70 (51%)<br>Other: 0 (0%)<br>Age mean (standard deviation): 32.10 (12.57)
Recruitment	In iSPOT-D, participants were adult outpatients with nonpsychotic major depressive disorder under 65 years of age.<br>In RAD, participants were patients from a community mental health training clinic, and individuals from the immediate surrounding community currently reporting distress from anxiety and related mood symptoms.<br>In HCP-DES, participants were individuals from the community experiencing significant symptoms of depression and anxiety (clinical participants) or individuals not experiencing any symptoms of emotional distress (healthy controls).<br>In ENGAGE, participants were adult patients receiving primary care at Palo Alto Medical Foundation, who had comorbid depression and obesity.
Ethics oversight	All participants provided written informed consent. Procedures were approved by the Stanford University Institutional Review Board (IRB 27937 and 41837) or the Western Sydney Area Health Service Human Research Ethics Committee.

Note that full information on the approval of the study protocol must also be provided in the manuscript.

Field-specific reporting

Please select the one below that is the best fit for your research. If you are not sure, read the appropriate sections before making your selection.

☒ Life sciences ☐ Behavioural & social sciences ☐ Ecological, evolutionary & environmental sciences

For a reference copy of the document with all sections, see nature.com/documents/nr-reporting-summary-flat.pdf

Life sciences study design

All studies must disclose on these points even when the disclosure is negative.

Sample size	The data used in this paper were aggregated from four completed studies: “International Study to Predict Optimized Treatment in Depression” (iSPOT-D, (Williams et al., 2011)), “Research on Anxiety and Depression study” (RAD, (Williams et al., 2016)), “Human Connectome Project for Disordered Emotional States” (HCP-DES, (Tozzi et al., 2020c)), and “Engaging self-regulation targets to understand the mechanisms of behavior change and improve mood and weight outcome” (ENGAGE, (Williams et al., 2018)).<br><br>The sample size was all the patients who had received fMRI as part of those studies. No sample size calculation was performed.

Data exclusions	Brain scans were discarded if they contained incidental findings, major scanner artifacts, signal dropouts, or had more than 25% of volumes containing significant frame-wise displacement as defined by fMRIprep.
Replication	Assessment of cluster stability using cross-validation<br>We adapted the procedure proposed by Dinga et al. (ref. 14) to our application to evaluate whether the clustering assignment was stable under small perturbations to the data. This enabled us to assess whether repeating the same procedure using a similar dataset would identify similar clusters, and whether we would we assign the same participants to the same clusters. In this analysis, we repeated the clustering procedure 801 times, each time with one participant left out. For each run and for each solution between 2 and 15 clusters, we calculated the similarity of the new cluster assignments to those from the original analysis using the adjusted Rand index (ARI), which is the corrected-for-chance version of the Rand index (ARI=0 corresponds to chance, ARI=1 is a perfect match, ARI<0 is a result worse than chance) (Figure 2d). We then repeated this procedure while holding out 20% of the sample instead of one participant (Figure 2e).<br><br>Split-half replication of cluster profiles<br>To verify if our final clustering solution was robust, we performed a split-half procedure as follows. First, we split our dataset into two random samples of equal size. Then, we ran our clustering procedure on the first half-split. Then, we assigned each participant in the second split to one of the clusters obtained in the first half-split. To do so, we computed the mean circuit scores across all participants belonging to each cluster in the first half-split. Then, we calculated the Pearson correlation coefficient between each participant's entire brain circuit score profile and these cluster-averaged scores. Each out-of-sample participant was assigned to the cluster for which this correlation was highest. Finally, we identified the primary circuit dysfunctions of each cluster in each split as described above (>0.5 SD absolute mean difference compared to the healthy reference data) and examined whether they replicated the circuit profiles found in the whole sample visually and by computing the Pearson correlation coefficient of the mean profile dysfunction profile of each cluster between splits (Figure 2f).<br><br>Split-half replication of associations between biotypes and clinical measures<br>We replicated the significant comparisons of behavior and symptoms between biotypes found in the complete sample by splitting the sample into two random halves, repeating the clustering procedure on the first half, and then using the circuit profile correlations described above to assign participants in the second half to the clusters obtained in the first half. We then conducted Wilcoxon tests as described above in each split and considered a result replicable if it was significant both in the original sample and in each of the split-half samples (for the second split we conducted a confirmatory one-sided test). We also calculated the clinical meaningfulness of results in both splits based on the effect size r, calculated as the Z statistic divided by square root of the sample size.<br><br>Leave-study-out replication of associations between biotypes and clinical measures<br>For each of the four studies included in our original dataset, we replicated the significant comparisons of behavior and symptoms between biotypes by splitting the sample into two subsets: one containing all the participants who were not from that study and one containing all participants from that study. Then, we repeated the clustering procedure on the first subset and then assigned participants in the second subset to the clusters obtained in the first subset using circuit profile correlations as described above. We then conducted Wilcoxon tests as described above and considered a result replicable if it was significant in each of the subsets for at least one held out study. For the leave-study-out replication, we conducted a confirmatory one-sided test. We also calculated the clinical meaningfulness of results in both splits based on the effect size r, calculated as the Z statistic divided by square root of the sample size.
Randomization	In iSPOT-D, participants were randomly allocated to receive escitalopram, sertraline or venlafaxine.<br>In ENGAGE, participants were randomly allocated to receive I-CARE behavioral treatment or treatment as usual.<br>In the current study, no allocation into experimental groups was performed.
Blinding	In iSPOT-D, the personnel doing the data acquisition was blind to the randomization.<br>In the current study, the analyst was not blinded to the randomization in the clinical trial data. Blinding was not relevant for this study, since the analysis was retrospective and compared clinical variables of groups of patients defined based on their brain characteristics. The analysis was not aimed at demonstrating the efficacy of a treatment.

Reporting for specific materials, systems and methods

We require information from authors about some types of materials, experimental systems and methods used in many studies. Here, indicate whether each material, system or method listed is relevant to your study. If you are not sure if a list item applies to your research, read the appropriate section before selecting a response.

Materials & experimental systems

n/a	Involved in the study
☒	☐ Antibodies
☒	☐ Eukaryotic cell lines
☒	☐ Palaeontology and archaeology
☒	☐ Animals and other organisms
☐	☒ Clinical data
☒	☐ Dual use research of concern
☒	☐ Plants

Methods

n/a	Involved in the study
☒	☐ ChIP-seq
☒	☐ Flow cytometry
☐	☒ MRI-based neuroimaging

Clinical data

Policy information about clinical studies

All manuscripts should comply with the ICMJE guidelines for publication of clinical research and a completed CONSORT checklist must be included with all submissions.

Clinical trial registration	iSPOT-D: https://clinicaltrials.gov/ct2/show/NCT00693849<br>ENGAGE: https://clinicaltrials.gov/ct2/show/NCT02246413
Study protocol	iSPOT-D: https://www.ncbi.nlm.nih.gov/pmc/articles/PMC3036635/<br>RAD: https://pubmed.ncbi.nlm.nih.gov/26980207/<br>HCP-DES: https://pubmed.ncbi.nlm.nih.gov/32147367/<br>ENGAGE: https://www.ncbi.nlm.nih.gov/pmc/articles/PMC8109191/
Data collection	Data previously collected was re-analyzed for the current study.
Outcomes	For clinical trial data, the outcomes were the ones defined in the protocols and the registration specified above.<br>For the current study, the analysis was exploratory, no primary or secondary outcomes were pre-defined.

Plants

Seed stocks	n/a
Novel plant genotypes	n/a
Authentication	n/a

Magnetic resonance imaging

Experimental design

Design type	Task (block and event related) and resting state
Design specifications	Facial Expressions of Emotion Task<br>A standardized set of 3D-evoked facial expression stimuli were presented in pseudorandom order, with five repeated blocks of eight stimuli per block for sad, threat evoked by fear, threat evoked by anger, and happy, relative to neutral blocks; duration of stimulus was 500ms and the interstimulus interval was 750ms (Korgaonkar et al., 2013). Participants were instructed to actively attend in order to answer post-scan questions about these faces, and we monitored alertness using an eye tracking system. We also presented the same stimuli nonconsciously in a backward-masking design to prevent awareness; face stimuli were presented for 10 ms followed immediately by a neutral face mask stimulus for 150 ms, and with a stimulus onset asynchrony of 1250 ms to match that of the conscious condition (Williams et al., 2006).<br><br>Go-NoGo Task<br>'Go' trials (the word "press" in GREEN) required participants to respond as quickly as possible, while the 'NoGo' trials ("press" in RED) required participants to withhold responses. 180 Go and 60 NoGo stimuli were presented in pseudorandom order; stimulus duration was 500 ms each with an interstimulus interval of 750 ms (Grieve et al., 2013).
Behavioral performance measures	Correct button presses and response times were not used in the analysis.

Acquisition

Imaging type(s)	Structural, functional
Field strength	3 T
Sequence & imaging parameters	MRI data was collected using a 3.0 Tesla GE Signa HDx (Sydney), a 3.0 Tesla GE MR750 Discovery (Stanford) and a 3.0 Tesla GE UHP (Stanford) (GE Healthcare, Milwaukee, Wisconsin) using an 8-channel head coil (Sydney) and 32-channel head coil (Stanford). The two Stanford scanners used identical sequences. Head motion was restricted with foam pads and participant alertness was monitored using an eye-tracking system. Head motion was also recorded, which was later subject to quality control and potential data exclusion on the premise of excess motion.<br><br>Stanford Sequences (RAD, HCP-DES, ENGAGE)<br>In RAD and ENGAGE, a T1-weighted structural scan was acquired using a 3D spoiled gradient echo (SPGR) sequence

normalization into standard space: TR=0.008; TE=0.003; voxel size=1x1x1mm; number of slices=176; FOV=256x256; flip angle=11o. In HCP-DES, the T1 parameters were TE = 3.548 ms; MPRAGE TR = 2.84s; FA = 8, acquisition time = 8 min and 33 sec; field of view = 256 × 256 mm; 3D matrix size = 320 × 320 × 230; slice orientation = sagittal; angulation to AC-PC line; receiver bandwidth = 31.25 kHz; fat suppression = no; motion correction = PROMO; voxel size = 0.8 mm isotropic. Blood oxygenation level-dependent contrast functional images were acquired using echo-planar T2*-weighted imaging. Each whole brain volume consisted of 45 interleaved 3mm thick axial/oblique slices (74 x 74 matrix; TR=2000ms; TE=27.5ms; voxel size=3x3x3mm; FOV=222mm; flip angle=77°). Each of the three tasks acquired 154 volumes over 5 minutes and 8 seconds.

Sydney Sequences (iSPOT-D)
The T1-weighted structural scan was acquired in the sagittal plane using a 3D spoiled gradient echo (SPGR) sequence (TR = 8.3 ms; TE = 3.2 ms; flip angle = 11 degrees; TI = 500 ms; NEX = 1; ASSSET = 1.5; matrix = 256 x 256). A total of 180 contiguous slices, each 1 mm thick, covered the whole brain with an in-plane resolution of 1 mm x 1 mm. The functional images for each task were acquired using echo planar imaging (TR = 2500 ms; TE = 27.5 ms; matrix = 64 x 64; FOV = 24 cm; flip angle = 90 degrees). Forty slices, each 3.5 mm thick, covered the whole brain in each volume. Each of the three tasks acquired 123 volumes over 5 minutes and 8 seconds.

Area of acquisition: Whole brain

Diffusion MRI ☐ Used ☒ Not used

Preprocessing

Preprocessing software: For functional images, the first three volumes were removed to account for magnetization transfer artifacts before pre-processing. Pre-processing was performed using fMRIPrep 20.2.1 (iSPOTD) and fMRIPrep 20.2.3 (HCP-DES, ENGAGE, RAD) (Esteban et al., 2019). For details, the standardized methodology outputs from fMRIPrep for each study can be found at the end of the Supplementary Material.

Normalization:

Anatomical data preprocessing
A total of 1 T1-weighted (T1w) images were found within the input BIDS dataset.The T1-weighted (T1w) image was corrected for intensity non-uniformity (INU) with N4BiasFieldCorrection (Tustison et al. 2010), distributed with ANTs 2.3.3 (Avants et al. 2008, RRID:SCR_004757), and used as T1w-reference throughout the workflow. The T1w-reference was then skull-stripped with a Nipype implementation of the antsBrainExtraction.sh workflow (from ANTs), using OASIS30ANTs as target template. Brain tissue segmentation of cerebrospinal fluid (CSF), white-matter (WM) and gray-matter (GM) was performed on the brain-extracted T1w using fast (FSL 5.0.9, RRID:SCR_002823, Zhang, Brady, and Smith 2001). Brain surfaces were reconstructed using recon-all (FreeSurfer 6.0.1, RRID:SCR_001847, Dale, Fischl, and Sereno 1999), and the brain mask estimated previously was refined with a custom variation of the method to reconcile ANTs-derived and FreeSurfer-derived segmentations of the cortical gray-matter of Mindboggle (RRID:SCR_002438, Klein et al. 2017). Volume-based spatial normalization to two standard spaces (MNI152NLin6Asym, MNI152NLin2009cAsym) was performed through nonlinear registration with antsRegistration (ANTs 2.3.3), using brain-extracted versions of both T1w reference and the T1w template. The following templates were selected for spatial normalization: FSL's MNI ICBM 152 non-linear 6th Generation Asymmetric Average Brain Stereotaxic Registration Model [Evans et al. (2012), RRID:SCR_002823; TemplateFlow ID: MNI152NLin6Asym], ICBM 152 Nonlinear Asymmetrical template version 2009c [Fonov et al. (2009), RRID:SCR_008796; TemplateFlow ID: MNI152NLin2009cAsym],

Functional data preprocessing
For each of the BOLD runs found per subject (across all tasks and sessions), the following preprocessing was performed. First, a reference volume and its skull-stripped version were generated using a custom methodology of fMRIPrep . Susceptibility distortion correction (SDC) was omitted. The BOLD reference was then co-registered to the T1w reference using bbregister (FreeSurfer) which implements boundary-based registration (Greve and Fischl 2009). Co-registration was configured with six degrees of freedom. Head-motion parameters with respect to the BOLD reference (transformation matrices, and six corresponding rotation and translation parameters) are estimated before any spatiotemporal filtering using mcflirt (FSL 5.0.9, Jenkinson et al. 2002). BOLD runs were slice-time corrected using 3dTshift from AFNI 20160207 (Cox and Hyde 1997, RRID:SCR_005927). The BOLD time-series were resampled onto the following surfaces (FreeSurfer reconstruction nomenclature): fsnative, fsaverage. The BOLD time-series (including slice-timing correction when applied) were resampled onto their original, native space by applying the transforms to correct for head-motion. These resampled BOLD time-series will be referred to as preprocessed BOLD in original space, or just preprocessed BOLD. The BOLD time-series were resampled into standard space, generating a preprocessed BOLD run in MNI152NLin6Asym space. First, a reference volume and its skull-stripped version were generated using a custom methodology of fMRIPrep .

Normalization template: MNI152NLin6Asym

Noise and artifact removal: In this analysis, a 128s high pass filter was applied to the data, and six realignment parameters as well as white matter and cerebrospinal fluid signals derived by fMRIPrep were added to the design matrix as confounds.

Volume censoring: None

Statistical modeling & inference

Model type and settings: Task-evoked activation was quantified using a generalized linear model (GLM) in which task events were convolved with a canonical hemodynamic response function as implemented in SPM8. In this analysis, a 128s high pass filter was applied to the data, and six realignment parameters as well as white matter and cerebrospinal fluid signals derived by fMRIPrep were added to the design matrix as confounds.

To quantify task-based functional connectivity, we computed psychophysiological interactions (PPI) between pairs of regions belonging to the same circuit. For each region in each circuit (PPI seed), we calculated the first eigenvariate of that region's time series and fit a whole-brain first-level GLM as described above, which consisted of the psychological variable (task contrast of interest), physiological variable (region time course), and the interaction between psychological and physiological variables (PPI effect of interest). Then, we computed the average PPI effect of interest in specific regions belonging to the same circuit in accordance with our hypothesized model of circuit dysfunction (PPI targets). To account for the fact that regions were used once as PPI targets and once as PPI seeds in this calculation, we averaged these results, yielding a single PPI value for each connection.

Effect(s) tested

Specific contrasts of interest were computed for each task and circuit as follows: 1) negative affect circuit: sad > neutral conscious faces; 2) negative affect circuit: threat > neutral conscious faces; 3) negative affect circuit: threat > neutral non-conscious faces; 4) positive affect circuit: happy > neutral conscious faces; 5) cognitive control circuit: NoGo > Go trials. Measures of activation for each region of each circuit were obtained by extracting the average value of the contrast of interest.

Specify type of analysis: ☐ Whole brain ☒ ROI-based ☐ Both

Anatomical location(s)

The regions of interest that comprise each circuit were defined from the meta-analytic database Neurosynth (Yarkoni et al., 2011) and then refined by removing regions that did not pass quality control or for which circuit quantification did not meet a set of psychometric criteria, such as construct validity, internal consistency, and independence. Of the remaining regions, we only retained those which were also implicated in our theoretical synthesis of dysfunctions in depression and anxiety, for a final set of 29 regions of interest (Goldstein-Piekarski et al., 2021; Williams, 2016b).

Statistic type for inference

(See Eklund et al. 2016)

None.

Correction

None.

Models & analysis

n/a	Involved in the study
☐	☒ Functional and/or effective connectivity
☒	☐ Graph analysis
☒	☐ Multivariate modeling or predictive analysis

Functional and/or effective connectivity

To quantify task-based functional connectivity, we computed psychophysiological interactions (PPI) between pairs of regions belonging to the same circuit. For each region in each circuit (PPI seed), we calculated the first eigenvariate of that region's time series and fit a whole-brain first-level GLM as described above, which consisted of the psychological variable (task contrast of interest), physiological variable (region time course), and the interaction between psychological and physiological variables (PPI effect of interest). Then, we computed the average PPI effect of interest in specific regions belonging to the same circuit in accordance with our hypothesized model of circuit dysfunction (PPI targets) (Figure 1). To account for the fact that regions were used once as PPI targets and once as PPI seeds in this calculation, we averaged these results, yielding a single PPI value for each connection.
